# Partitioning of herbivore hosts across time and food plants promotes diversification in the *Megastigmus dorsalis* oak gall parasitoid complex

**DOI:** 10.1002/ece3.3712

**Published:** 2017-12-25

**Authors:** James A. Nicholls, Karsten Schönrogge, Sonja Preuss, Graham N. Stone

**Affiliations:** ^1^ Ashworth Labs Institute of Evolutionary Biology University of Edinburgh Edinburgh UK; ^2^ Centre for Ecology and Hydrology Wallingford, Oxfordshire UK; ^3^ Present address: Uppsala County Administrative Board Uppsala Sweden

**Keywords:** community evolution, Cynipidae, ecological speciation, host race, *Megastigmus dorsalis*, specialization

## Abstract

Communities of insect herbivores and their natural enemies are rich and ecologically crucial components of terrestrial biodiversity. Understanding the processes that promote their origin and maintenance is thus of considerable interest. One major proposed mechanism is ecological speciation through host‐associated differentiation (HAD), the divergence of a polyphagous species first into ecological host races and eventually into more specialized daughter species. The rich chalcid parasitoid communities attacking cynipid oak gall wasp hosts are structured by multiple host traits, including food plant taxon, host gall phenology, and gall structure. Here, we ask whether the same traits structure genetic diversity within supposedly generalist parasitoid morphospecies. We use mitochondrial DNA sequences and microsatellite genotypes to quantify HAD for *Megastigmus* (*Bootanomyia*) *dorsalis*, a complex of two apparently generalist cryptic parasitoid species attacking oak galls. Ancient Balkan refugial populations showed phenological separation between the cryptic species, one primarily attacking spring galls, and the other mainly attacking autumn galls. The spring species also contained host races specializing on galls developing on different host‐plant lineages (sections *Cerris* vs. *Quercus*) within the oak genus *Quercus*. These results indicate more significant host‐associated structuring within oak gall parasitoid communities than previously thought and support ecological theory predicting the evolution of specialist lineages within generalist parasitoids. In contrast, UK populations of the autumn cryptic species associated with both native and recently invading oak gall wasps showed no evidence of population differentiation, implying rapid recruitment of native parasitoid populations onto invading hosts, and hence potential for natural biological control. This is of significance given recent rapid range expansion of the economically damaging chestnut gall wasp, *Dryocosmus kuriphilus*, in Europe.

## 1 INTRODUCTION

Insect communities comprising insect herbivores and their parasitoid natural enemies dominate terrestrial animal biodiversity and fulfill multiple ecological roles (Feder & Forbes, 2010; Futuyma & Agrawal, 2009; May, 1990; Santos & Quicke, 2011). There is a general consensus that much of herbivorous insect diversity has arisen through specialization to particular host‐plant lineages, with ongoing arms race coevolution between plant defenses and herbivore countermeasures (Endara et al., 2017; Janz, 2011; Marquis et al., 2016; Matsubayashi, Ohshima, & Nosil, 2009). Work in a growing number of systems shows that insect herbivore morphospecies often harbor genetically diverging lineages, termed ecological host races or biotypes, that specialize on a subset of the full host‐plant range (Abrahamson, Blair, Eubanks, & Morehead, 2003; Drès & Mallet, 2002; Linn et al., 2003; Lozier, Roderick, & Mills, 2007; Powell, Forbes, Hood, & Feder, 2014).

In addition to herbivores, host race formation (also termed host‐associated differentiation, HAD) can have cascading effects across higher trophic levels (Abrahamson Blair, Eubanks, & Morehead, 2003; Feder & Forbes, 2010; Price et al., 1980; Stireman, Nason, Heard, & Seehawer, 2006). Host‐associated differentiation is also thought to be important in diversification of the parasitoid natural enemies that impose high mortality on many insect herbivores (Bird, Fernandez‐Silva, Skillings, & Toonen, 2012; Loxdale, Lushai, & Harvey, 2011). Host‐associated differentiation within parasitoid taxa is of particular interest because it could resolve an apparent paradox present in many communities comprising specific guilds of insect herbivores (such as leaf miners or gall inducers) and their parasitoid natural enemies. Such communities often combine both high species richness of herbivore hosts, and large numbers of generalist parasitoids (those attacking a wide host range). The paradox is that we expect generalist parasitoids to reduce host species richness because, in the absence of other structuring processes, parasitoid populations that result from attack of one host can have negative impacts on other hosts and potentially drive these to extinction, a process termed apparent competition (Holt & Lawton, 1993; Morris, Lewis, & Godfray, 2004; Stone & Schönrogge, 2003). High herbivore host diversity and shared generalist natural enemies can be reconciled if apparent generalist enemies in fact comprise genetically divergent cryptic lineages that each attack only a subset of the species’ recorded host range. Such structuring has been revealed in a range of systems (Forbes, Powell, Stelinski, Smith, & Feder, 2009; Hood et al., 2015; Smith, Wood, Janzen, Hallwachs, & Hebert, 2007; Smith et al., 2008; Stireman et al., 2006). More broadly, understanding how insect herbivores and parasitoid communities are structured has implications for many aspects of ecosystem management, including biological control of herbivorous pests (Carvalheiro, Buckley, Ventim, Fowler, & Memmott, 2008; Henneman & Memmott, 2001), and predicting the impacts of range expansions associated with anthropogenic introductions and climate change (Nicholls, Fuentes‐Utrilla, et al., 2010; Sax et al., 2007).

Here, we explore the potential for HAD in an apparently generalist chalcid parasitoid associated with a speciose guild of insect herbivores, oak cynipid gall wasps (Hymenoptera: Cynipidae) in the Western Palaearctic. Host galls vary in several traits that are known to determine parasitoid community composition (Bailey et al., 2009), including gall phenology, food plant taxon, and gall location on the plant (explained below). The same host traits could thus potentially also structure parasitoid populations. Each of the approximately 150 oak gall wasp species in the Western Palaearctic has one spring generation and one autumn generation per year (Stone et al., 2008), providing the potential for parasitoids to evolve seasonal phenologies that allow attack of one or both generations. Gall wasp generations also show high host‐plant specificity, and in the Western Palaearctic, almost all oak cynipid galls develop only on oak species from one (but not both) of the native oak sections *Quercus* or *Cerris* (Stone et al., 2009). Finally, gall wasps also differ in the oak organ they induce their galls on (e.g., leaves, buds, acorns, roots, stems, or catkins; Cook, Rokas, Pagel, & Stone, 2002). Western Palaearctic oak cynipid galls are attacked by over 100 morphologically recognizable parasitoid species, all of which attack multiple cynipid host species (Askew et al., 2013). The host gall traits listed above have been shown to predict which parasitoids attack which galls, with food plant taxon playing a dominant role (Askew, 1961; Bailey et al., 2009). However, rather than strict specialization of particular parasitoid species to specific host galls, this structuring reflects the relative preferences of generalist parasitoids for sets of host galls showing particular phenotypic trait combinations. Herbivorous gall wasp niches have probably been shaped both by bottom‐up interactions with their plant hosts, and top‐down interactions with their parasitoid natural enemies, a situation encapsulated in the tri‐trophic niche concept (Singer & Stireman, 2005) that could help maintain the high species‐level diversity in this community (Askew, 1980). Here, we extend this work by asking whether the same gall traits that structure parasitoid communities (phenology, plant organ galled, and the section‐level identity of the food plant) also structure genetic variation within an apparently generalist parasitoid.

Interest in host‐associated differentiation in cynipid galls has grown in response to the detection of morphologically cryptic parasitoid species using molecular approaches (Kaartinen, Stone, Hearn, Lohse, & Roslin, 2010; Nicholls, Preuss, et al., 2010). Several of the parasitoid morphospecies studied by Bailey et al. (2009) and Askew (1961) are now known to contain cryptic species, indicating that the richness and complexity of the oak gall wasp system are higher than previously thought. Only one study has so far examined the host associations of cryptic parasitoid species, finding them to be more specialized than their corresponding morphospecies (Kaartinen et al., 2010). However, the extent to which morphologically cryptic species complexes show host‐associated differentiation, whether between or within species, remains unknown.

Here, we examine genetic structure in the parasitoid *Megastigmus dorsalis* (Hymenoptera: Torymidae; also called *Bootanomyia dorsalis* following Doganlar, 2011), a morphospecies complex that has a particularly broad host gall range (Askew et al., 2013). This allows assessment of the impact on genetic structure within a species complex of the same host traits that are known to structure patterns across parasitoid species. Previous molecular analysis of the *M. dorsalis* complex using sequence data for the mitochondrial cytochrome *b* gene (cytb) and three unlinked nuclear markers (Nicholls, Preuss, et al., 2010) has shown it to contain two cryptic species (hereafter called *M. dorsalis* sp.1 and *M. dorsalis* sp.2), identifiable with 100% accuracy using cytb haplotypes alone. Both cryptic species have distributions that span the Western Palaearctic from the Iberian Peninsula to Iran, and both are also found in northern Europe, including the UK (Nicholls, Preuss, et al., 2010). Broad overlap in their distributions means that previous host gall wasp records for the morphospecies *M. dorsalis* cannot be retrospectively assigned either cryptic species on the basis of geography. Host gall wasp associations for the two cryptic species must thus be established de novo by sequence‐based identification of emerging insects (see also Kaartinen et al., 2010).

We used this approach to establish the cryptic species status of 368 individuals from the *M. dorsalis* morphospecies complex and examined host‐associated genetic structure between and within the cryptic species at two temporal and spatial scales. First, we examine the impact of host gall traits on long‐term patterns of host‐associated genetic structure in 316 *M. dorsalis* sampled in Hungary. These samples are from a known glacial refuge area in which oak gall wasps populations are thought to have persisted for hundreds of thousands of years (Stone et al., 2012). We use parasitoid samples reared from host galls whose traits span the known diversity for hosts of *M. dorsalis* to ask the following questions:

1. Do the two cryptic species differ in the spectra of host gall wasps they attack, with particular respect to oak food plant, gall phenology, and gall location on the food plant?
2. Are any of these host traits associated with genetic structure within a cryptic species? For the second question, we focus on *M. dorsalis* sp.2 shown by previous work to have high intraspecific genetic diversity (in contrast to *M. dorsalis* sp.1, which has much less; Nicholls, Preuss, et al., 2010). If patterns within species parallel those found across species (Bailey et al., 2009), we expect a dominant role for structuring by oak food plant.

On a second temporal scale of just several hundred years, we examine evidence for more recently established host association patterns using a further 52 samples collected in the United Kingdom. Since the 1840s, the UK has been invaded by a suite of oak gall wasp species from southern European refugial areas following anthropogenic planting of Turkey oak (*Q. cerris*) throughout northern Europe (Schönrogge et al., 2012; Stone et al., 2007). Genetic evidence indicates that invading gall wasp populations originated in the Balkans (Stone, Atkinson, Rokas, Csóka, & Nieves‐Aldrey, 2001; Stone & Sunnucks, 1993; Stone et al., 2007). These invading gall wasps represent different gall trait combinations to those present in native oak gall wasp species, providing a large‐scale natural experiment on the impact of host gall traits on the development of parasitoid population structure on a timescale of decades to centuries. All of the invading gall wasps are now attacked in the UK by multiple parasitoid species, including *M. dorsalis* (Schönrogge, Walker, & Crawley, 1998; Schönrogge et al., 2012). Two alternative hypotheses exist for the recruitment of parasitoids to the invading gall wasps: local recruitment of native UK parasitoid populations onto the invading hosts (Schönrogge, Walker, & Crawley, 2000), and pursuit of invading hosts by coinvading non‐native parasitoids (Hayward & Stone, 2006; Nicholls, Fuentes‐Utrilla, et al., 2010). Both are possible for recruitment of *M. dorsalis* in the UK. The availability of native UK populations for local recruitment is supported by the fact that *M. dorsalis* was first described from northern European specimens in 1798 and is common in regions (such as Finland; Kaartinen et al., 2010) that have yet to be colonized by invading southern gall wasp species. Potential for host pursuit is supported by the abundance of *M. dorsalis* as a parasitoid of potential source populations of the invading gall wasps in southern Europe (Askew et al., 2013; Schönrogge, Stone, & Crawley, 1995). The local recruitment and host pursuit hypotheses make contrasting predictions for patterns for genetic diversity in *M. dorsalis* reared from native and invading host galls in the UK. If native *M. dorsalis* are attacking invading hosts, then we expect no genetic divergence between individuals reared from native and invading gall wasp hosts in the UK, but divergence of both of these from southern refugial populations. In contrast, if invading gall wasps in the UK are attacked primarily by non‐native parasitoids that have pursued them from the Balkans, then we predict genetic differentiation between *M. dorsalis* reared from native and invading hosts in the UK, but genetic similarity between individuals reared from invading hosts and those sampled from potential source populations in southern and central Europe. We thus ask a third question:

- Are patterns of genetic diversity in UK *M. dorsalis* reared from invading host galls more compatible with the local recruitment or host pursuit hypothesis?

## 2 MATERIALS AND METHODS

### 2.1 Sampling

**Figure 1:**
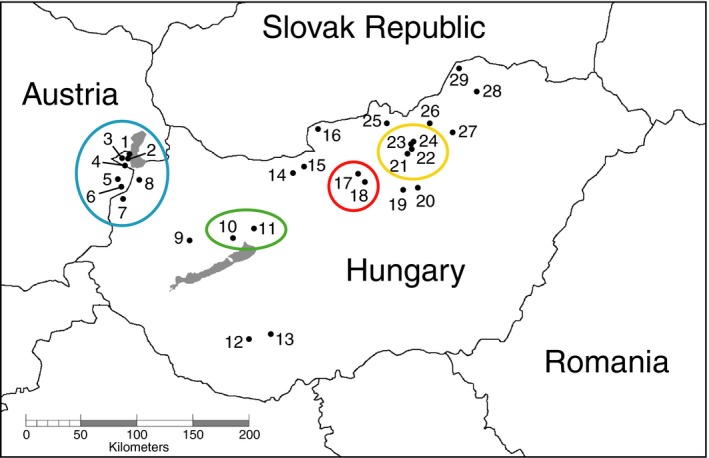
Map of Hungary and surrounding regions, with numbered dots indicating sampling locations of *Megastigmus dorsalis* individuals sequenced/genotyped in this study. Numbers correspond to sites named in Appendix S1. Colored circles surround sites that were lumped into the four regions used for AMOVA tests of geographic structuring

Sampling for this study included data for 35 Hungarian individuals sequenced by Nicholls, Preuss, et al. (2010), 33 of which were shown to be from *M. dorsalis* sp.1 and two from *M. dorsalis* sp.2, and a further 281 Hungarian individuals (of which 171 proved to be from *M. dorsalis* sp.1 and 110 from *M. dorsalis* sp.2). We generated novel microsatellite data for all 112 *M. dorsalis* sp.2 individuals. The UK sampling incorporates 20 individuals sequenced by Nicholls, Preuss, et al. (2010), and an additional 32 individuals. Cynipid oak galls were collected at multiple sites in Hungary and adjacent areas of Austria (henceforth simplified to “Hungary”) between 1999 and 2006 (Figure 1). Full metadata for all sampled galls and *M. dorsalis* specimens are given in Appendix S1. Galls were reared singly at ambient temperature, and emerging parasitoids were stored in 100% ethanol. *Megastigmus dorsalis* individuals were selected with no prior knowledge of their assignment to either cryptic genetic species in this complex. Sampling was balanced across host oak lineages (section *Cerris* vs. section *Quercus* oaks), and structured to include a maximum of 45 individuals obtained from as many of the major host gall wasp lineages as possible (either different gall wasp genera or major clades within the speciose genus *Andricus*, following the classification of Stone et al., 2009). Some oak galls contain multiple host larvae and can yield multiple parasitoid individuals, so for these only a single parasitoid per gall was selected to minimize the chance of sampling siblings. The UK field sampling followed similar protocols at multiple sites within the southern UK (Appendix S1). Specimen selection of the UK *M. dorsalis* was balanced across those emerging from native galls (*Andricus curvator*,* A. foecundatrix*,* Biorhiza pallida*) and those from galls of species that have recently invaded the UK from central and eastern Europe (*A. aries*,* A. grossulariae*,* A. kollari*,* A. quercuscalicis,* and *Aphelonyx cerricola*; Schönrogge et al., 2012).

### 2.2 Molecular methods

DNA was extracted using a Chelex method following Nicholls, Preuss, et al. (2010). All individuals were sequenced for a 433 base pair fragment of the mitochondrial cytochrome *b* gene using the primers CB1/CB2 or CB1/CP2 (see Nicholls, Preuss, et al., 2010 for details). This gene fragment provides unambiguous assignment of individuals to either *M. dorsalis* sp.1 or *M. dorsalis* sp.2 (Nicholls, Preuss, et al., 2010). Amplicons were cleaned using a SAP/ExoI protocol and sequenced in both directions using BigDye v3.1 terminator chemistry run on an ABI3730 capillary electrophoresis machine. Sequences were edited in Sequence Navigator v1.0.1 (Parker, 1997), and the LASERGENE package (DNAstar Inc, Madison WI, USA) was used to check for open reading frames and to align sequences.

Eight microsatellite loci were genotyped for all Hungarian individuals of *M. dorsalis* sp.2, the cryptic species previously shown to contain substantial genetic diversity (Nicholls, Preuss, et al., 2010), and that showed significant host‐associated genetic structure in the cytb data presented here. All loci (Mdo6, Mdo7A, Mdo9, Mdo12, Mdo16, Mst2, Mst13, and Mst14) were amplified following conditions in Garnier, Verduijn, Preuss, Wolff, and Stone (2008). Amplicons were run on an ABI 3730 capillary machine, and allele sizes scored using GeneMapper v4.0 (Applied Biosystems, Foster City CA, USA).

### 2.3 Microsatellite analyses

Population structure within the eight‐locus microsatellite data from *M. dorsalis* sp.2 was examined using the Bayesian model‐based clustering algorithm implemented in Structure v2.3.2 (Pritchard, Stephens, & Donnelly, 2000). Both diploid females and haploid males were included, with males coded as missing one allele per locus. Initial exploratory analyses assuming a single population were conducted to determine the value of λ (the parameter describing the allele frequency distribution) in models with or without admixture. In both cases, λ was 0.638, and this value was fixed in all subsequent analyses. The best choice of ancestry and allele frequency models was determined by conducting two runs for each combination of models with/without admixture and with correlated/uncorrelated allele frequencies for numbers of assumed populations (*K*) ranging from 1 to 8. In admixture models, alpha was estimated and assumed to be equal across populations. No prior information on population assignment was used. All runs had a burn‐in of 100,000 states followed by 1,000,000 iterations. Having determined the best model (no admixture with correlated allele frequencies), three further runs of this model were conducted for *K* from 1 to 8, giving five runs for each value. The value Δ*K*, following Evanno, Regnaut, and Goudet (2005), was used to guide selection of the best‐supported *K* value. Results for the full dataset were compared with those for females only to check that results were robust to violating the assumption that all samples have the same ploidy level. No difference in the results was detected, so we present results for the full dataset below.

Our analyses reveal strong associations between the oak section on which a host gall developed and the genotype of associated *M. dorsalis* sp.2 individuals. However, a small number of individuals had nuclear/mitochondrial genotypic combinations that were inconsistent with the oak association of their host gall. We therefore conducted additional ancestry analyses in Structure incorporating host food plant information as a prior to assess whether such mismatches could be explained by introgression between populations attacking hosts on the different oak lineages. Six of the 112 individuals genotyped for microsatellites showed host associations inconsistent with expectations based on their nuclear genotype, so this frequency was used as the value for the migration prior (ν  =  0.05; the probability that an individual is genetically derived, or has recent ancestors, from the population attacking galls on the oak section it was not collected from). Two further analyses using *ν* = 0.1 and *ν* = 0.01 were also conducted to examine the robustness of the result to variation in the migration prior. Each analysis was run twice, with *K *=* *2 and assessing ancestors back two generations. Results were consistent over all three values of *ν*, so we present results for *ν* = 0.05.

Impacts of host traits on genetic variation within *M. dorsalis* sp.2 were assessed using analyses of molecular variance (AMOVA) in Arlequin v2.001 (Schneider, Roessli, & Excoffier, 2000). As with the Structure analyses, the second allele for haploid males was coded as missing. After demonstrating a lack of significant spatial substructure among Hungarian populations (see Results, Table 1a), all samples were pooled for the host trait analyses. We examined potential population structuring by three gall traits: the timing of host gall development (spring vs. late summer/autumn); the plant organ galled by the host gall wasp (using the categories acorn, bud, catkin, leaf, and shoot, following Bailey et al., 2009); and the major lineage of oak being galled. Since closely related gall wasps often share similar trait values (see Bailey et al., 2009), we reduced potential impacts of phylogenetic nonindependence by including the gall wasp genus (or major clade within the diverse genus *Andricus*, following Stone et al., 2009) as a level nested within the main comparison of interest. The full data structure for individuals in these analyses is provided in Appendix S1.

**Table 1:** Partitioning of genetic variation among Hungarian populations of the *Megastigmus dorsalis* morphospecies complex using hierarchical analyses of molecular variance (AMOVA). The four regions in the spatial analysis are indicated by colored circles in Figure 1. (a) test using eight microsatellite loci within *M. dorsalis* sp.2 only; (b) tests using cytochrome *b* haplotype data across all individuals within the morphospecies complex and within *M. dorsalis* sp.2 only

Taxon assessed	Source of variation	*df*	Variance component	% of total variation	*F*‐statistics	*p*‐value
(a) Microsatellite spatial structure among Hungarian regions						
*M. dorsalis* sp.2	Among regions	3	−0.098	−5.92	*F*_CT_ = −0.059	.915
	Among sites in regions	9	0.311	18.73	*F*_SC_ = 0.177	<.001
	Within sites	183	1.446	87.19	*F*_ST_ = 0.128	<.001
(b) Cytb spatial structure among Hungarian regions						
*M. dorsalis* morphosp.	Among regions	3	−0.036	−8.55	*F*_CT_ = −0.086	.984
	Among sites in regions	12	0.077	18.41	*F*_SC_ = 0.170	<.001
	Within sites	243	0.378	90.14	*F*_ST_ = 0.099	<.001
*M. dorsalis* sp.2	Among regions	3	−0.034	−7.03	*F*_CT_ = −0.070	.849
	Among sites in regions	9	0.110	23.07	*F*_SC_ = 0.216	<.001
	Within sites	85	0.402	83.95	*F*_ST_ = 0.160	<.001

### 2.4 Cytb sequence analyses

Individual‐level data across the *M. dorsalis* complex were collapsed into unique haplotypes, and individuals were allocated to one or other of the known cryptic species in this complex following phylogeny reconstruction using MrBayes v3.2.6 (Ronquist et al., 2012). One sequence was included from each of the other three *Megastigmus* species attacking oak gall wasps in the Western Palaearctic, *M. dumicola* (GenBank GU123593), *M. stigmatizans* (GenBank FJ026675), and *M. synophri* (GenBank GU123575), with *M. synophri* specified as the out‐group in the analysis following the phylogeny presented in Nicholls, Preuss, et al. (2010). Initial assessment of the base substitutions present in our data revealed limited informative variation in first or second positions, and some types of transversions were never observed. Hence, the data were divided into two partitions (combined 1st/2nd codon positions, and 3rd codon positions) with independent HKY + I + G substitution models estimated for each partition. Comparison of Bayes factors [estimated using twice the difference in the natural log of the harmonic mean of model likelihoods of each model (2ΔlnHML), and assessed following Table 2 of Kass and Raftery (1995)] indicated that a strict clock model incorporating a birth–death speciation process provided a better fit to the data than either a no‐clock model or a model fitting a strict clock incorporating a coalescent process (2ΔlnHML = 122 and 2ΔlnHML = 328, respectively), and that a relaxed clock was no better than a strict clock (2ΔlnHML = 1.44). Our final analysis thus utilized a model incorporating a strict clock and a birth–death process of lineage diversification, with relative substitution rates allowed to vary for each data partition. We carried out two independent MCMC runs, each comprising four chains (one cold, three heated, with a temperature setting of 0.12), running for 10 million generations and sampled every 2,000 generations. Convergence between runs, stationarity of parameters, and appropriate levels of chain swapping were assessed using Tracer version 1.6 (Rambaut & Drummond, 2007), and a 50% majority‐rule consensus tree was generated from samples taken during the last 3 million generations of each run.

**Table 2:** Sampling effort for both *Megastigmus dorsalis* cryptic species across the different levels of their hosts’ traits. Host gall clade follows genus‐level structure within oak gall wasps with the exception of the diverse genus *Andricus*, within which major clades are named following Stone et al. (2009)

	No. sp. 1 individuals	No. sp. 2 individuals	Total individuals
Oak lineage galled by host			
Section Cerris	116	49	165
Section Quercus	88	63	151
Phenology of host gall			
Spring gall	49	73	122
Autumn gall	155	39	194
Plant organ galled by host			
Acorn	18	8	26
Bud	107	49	156
Catkin	10	23	33
Leaf	42	6	48
Shoot	27	26	53
Host gall clade			
*A. foecundatrix* clade	‐	1	1
*A. inflator* clade	‐	8	8
*A. kollari* clade	35	6	41
*A. lucidus* clade	22	23	45
*A. multiplicatus* clade	41	6	47
*A. quercuscalicis* clade	26	14	40
*Aphelonyx*	29	‐	29
*Biorhiza*	6	34	40
*Callirhytis*	17	‐	17
*Cynips*	1	‐	1
*Pseudoneuroterus*	23	19	42
*Synophrus*	4	1	5

As with the microsatellite data, AMOVAs were conducted on the Hungarian cytb data to test for parasitoid genetic structuring in relation to host gall traits. All cytb AMOVA analyses were conducted both across all samples (to test for ecological divergence between the two cryptic species in the *M. dorsalis* complex) and within *M. dorsalis* sp.2 (to test for the presence of intraspecific host races). Again, after first determining that there was no spatial genetic structure within Hungary (Table 1b), we pooled relevant samples for analyses across and within species. Subsequent analyses testing for host‐related structure included host gall clade nested within the main trait being examined.

Three AMOVAs were conducted using the UK cytb haplotypic data. The first compared samples derived from native UK host galls with samples from Hungarian host galls, to establish whether underlying geographic structure was present to allow discrimination between the local recruitment and host pursuit hypotheses. Subsequent AMOVAs examined genetic differentiation between UK *M. dorsalis* emerging from invading hosts and either (a) native UK hosts or (b) Hungarian hosts.

A parsimony network was computed for the cytb haplotypes in each cryptic *M. dorsalis* species using the program TCS version 1.21 (Clement, Posada, & Crandall, 2000). Demographic signatures of population growth were tested using Tajima's D statistic for the same cytb datasets, calculated in Arlequin (Schneider et al., 2000).

## 3 RESULTS

### 3.1 Host‐associated differentiation in a long‐established refuge population

#### 3.1.1 Molecular diversity in cytb and microsatellite data

**Figure 2:**
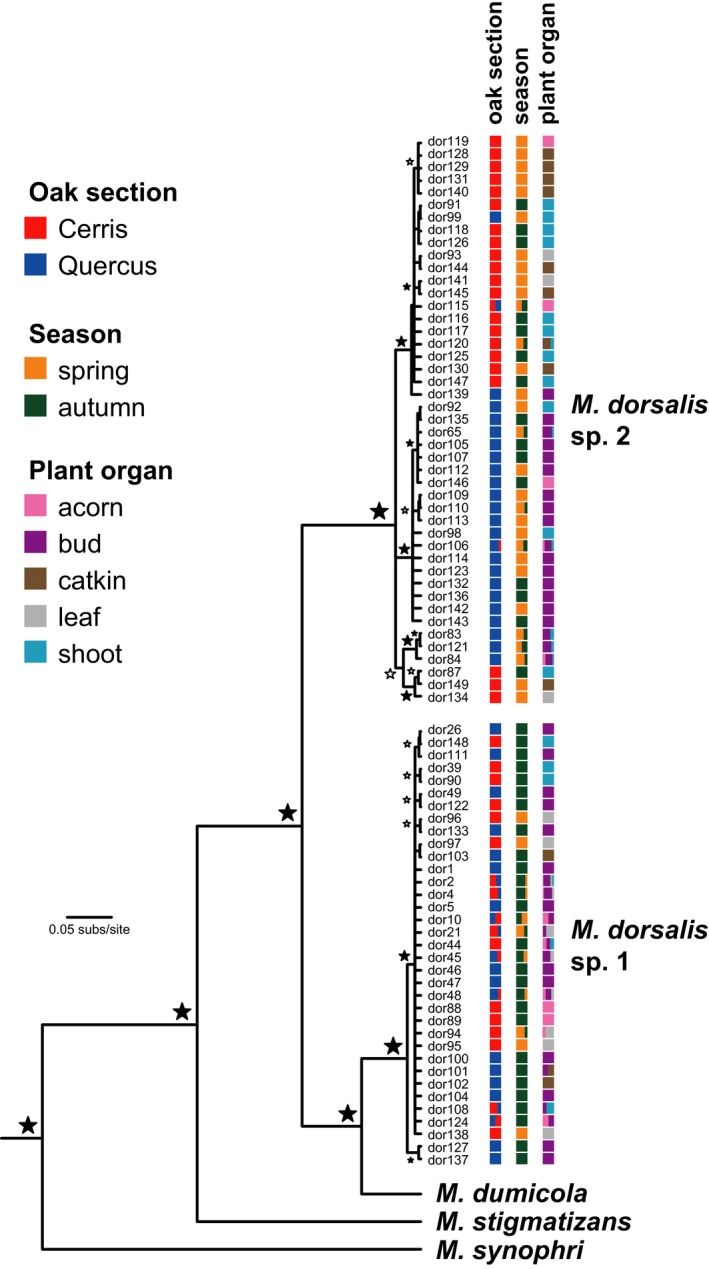
Bayesian 50% majority‐rule consensus tree for the 80 *Megastigmus dorsalis* cytochrome *b* haplotypes obtained for Hungarian samples in this study. Clades corresponding to the two cryptic species within the *M. dorsalis* morphospecies are indicated. Columns of colored squares next to haplotype names indicate the proportion of individuals with that haplotype that were collected from galls on different oak sections, in different seasons, or on different plant organs. Black stars next to nodes indicate ≥95% posterior probability node support, white stars indicate node support of ≥70%, other nodes present have posterior support of 50%–69%. Haplotypes are numbered as in Appendix S1

Cytb data for 316 Hungarian individuals of the *M. dorsalis* morphospecies contained 80 haplotypes, of which 35 were placed in *M. dorsalis* sp.1 (*n* = 204 individuals) as defined by Nicholls, Preuss, et al. (2010), while 45 were placed in *M. dorsalis* sp.2 (*n* = 112 individuals). Representative sequences for each haplotype have been deposited with GenBank (accessions within GU123486‐GU123573, and KX980093‐KX980153). Phylogenetic patterns of haplotype variation were consistent with those previously described by Nicholls, Preuss, et al. (2010), with deep divergence between the two cryptic species of *M. dorsalis* (Figure 2; mean haplotype divergence = 9.90%, range 8.08%–11.32%). *Megastigmus dorsalis* sp.1 showed very little haplotype diversity (mean divergence = 0.76%, range 0.23%–2.54%), while *M. dorsalis* sp.2 had higher diversity distributed among four well‐supported subclades (average haplotype divergence between subclades = 2.86% (range 1.39%–4.85%) and within subclades = 0.76% (range 0.23%–1.85%); Figure 2). Table 2 provides a summary of the distribution of the two cryptic *M. dorsalis* species across host traits of interest (oak lineage, season, plant organ, and gall wasp clade).

Microsatellite allelic data were obtained for eight loci for all 112 individuals of *M. dorsalis* sp.2, with the exception of four individuals for which one locus could not be scored (full dataset available in Appendix S2). Allelic richness ranged between 3 and 17 alleles per locus (average 10.1; Appendix S2). Most samples (88%) were obtained from four main regions within Hungary, each separated by 50–110 km (indicated by colored circles in Figure 1). AMOVA (Table 1a) showed that geographic region accounted for none of the genetic variation within this species, indicating considerable gene flow between sites and justifying pooling of samples for subsequent AMOVAs based on host gall traits.

#### 3.1.2 *M. dorsalis* cryptic species attack host galls in different seasons

Host gall phenology explained significant genetic structure within the *M. dorsalis* morphospecies complex, accounting for 6.4% of the cytb haplotype variation (*p *=* *.007; Table 3a). *Megastigmus dorsalis* sp.1 was more often associated with autumn galls, while *M. dorsalis* sp.2 was more commonly found in spring galls (Chi‐squared test, χ^2^ = 51.7, *df* = 1, *p *<* *.001; see also Figure 2). Gall phenology explained no significant intraspecific variation in *M. dorsalis* sp.2 for either the microsatellite or cytb data (*p *=* *1.00 for both marker types; Table 3a, b).

**Table 3:** Partitioning of genetic variation by host traits between the two cryptic species in the *Megastigmus dorsalis* complex and within *M. dorsalis* sp.2 using hierarchical analyses of molecular variance (AMOVA). (a) patterns in cytochrome *b* haplotype data; (b) patterns in eight microsatellite loci for *Megastigmus dorsalis* cryptic sp.2 only. Categories for the host gall traits are defined in the Materials and Methods. All analyses included host gall clade as a nesting variable (see Materials and Methods). Statistically significant effects are in bold

Host gall trait	Taxon	*df*	Variance component	% of total variation	Trait *F*‐statistic	*p*‐value
(a) Patterns in cytb haplotype data						
Host gall food plant	*M. dorsalis* morpho sp.	1	−0.003	−0.60	*F*_CT_ = −0.006	.486
	*M. dorsalis* sp.2	1	0.029	5.86	*F*_CT_ = 0.059	**.016**
Host gall phenology.	*M. dorsalis* morpho sp.	1	0.028	6.36	*F*_CT_ = 0.064	**.007**
	*M. dorsalis* sp.2	1	−0.019	−4.05	*F*_CT_ = −0.041	1.000
Host gall plant organ.	*M. dorsalis* morpho sp.	4	−0.009	−2.20	*F*_CT_ = −0.022	.779
	*M. dorsalis* sp.2	4	0.034	6.93	*F*_CT_ = 0.069	**.030**
(b) Patterns in microsatellite data						
Host gall food plant	*M. dorsalis* sp.2	1	0.244	13.61	*F*_CT_ = 0.136	**.008**
Host gall phenology	*M. dorsalis* sp.2	1	−0.087	−5.23	*F*_CT_ = −0.052	1.000
Host gall plant organ	*M. dorsalis* sp.2	4	0.106	6.22	*F*_CT_ = 0.062	.136

#### 3.1.3 *M. dorsalis* species 2 contains host races attacking galls on different host‐plant taxa

**Figure 3:**
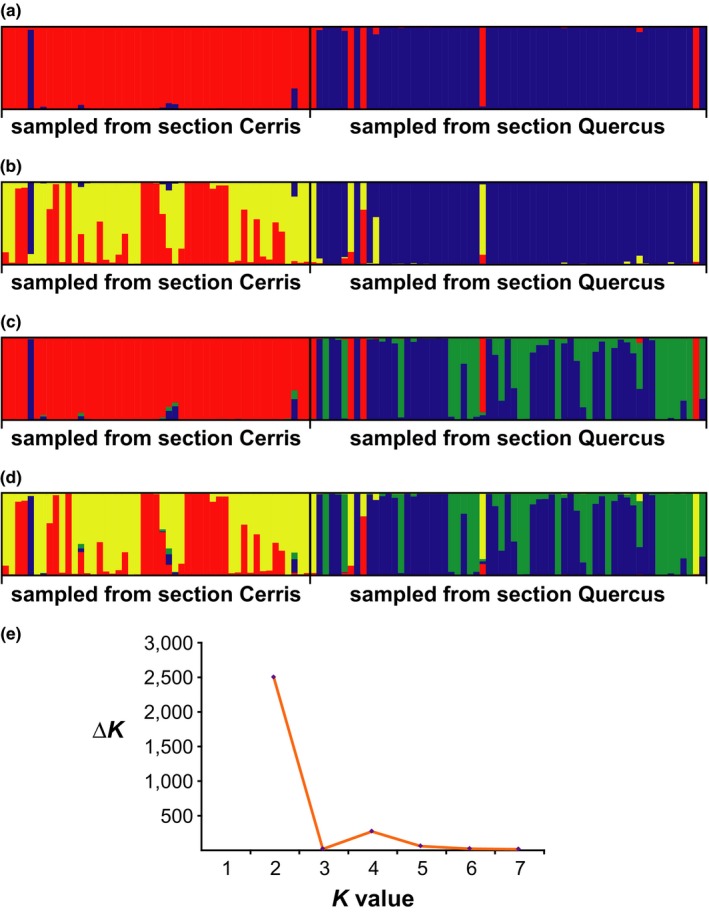
Allocation of individuals of *Megastigmus dorsalis* sp.2 to microsatellite genotype pools using Structure. From top to bottom, plots show results for (a) *K *=* *2*,* (b, c) two alternative solutions for *K *=* *3, and (d) *K *=* *4. Each sampled individual is represented by a column, with individuals arranged horizontally in the plot according to the oak section from which they were sampled. (e) Plot of the Δ*K* value for each value of *K* assessed, following Evanno et al. (2005)

Structure analysis of *M. dorsalis* sp.2 gave strongest support for *K *=* *2 populations, which correspond almost exactly to samples reared out of host galls from oaks in either section *Cerris* or section *Quercus* (Figure 3a,e). Almost all individuals were assigned to one or other cluster with a probability >.95 (Appendix S2). Analyses at higher values of *K* revealed additional structure, involving subdivision of one or both of the *K* = 2 clusters (Figure 3b–d). These subdivisions did not correlate with any obvious biological traits of the individuals assigned to them (e.g., spatial structure, host traits, and phenology). AMOVA revealed statistically significant genetic structuring by oak section within this species in both the microsatellite and cytb datasets (microsatellites: 13.6% of the variation, *p *=* *.008; Table 3b; cytb: 5.86% of the variation, *p *=* *.016; Table 3a). This structure is apparent in the cytb phylogeny, with the major subclades within *M. dorsalis* sp.2 being restricted (or almost so) to either the oak section *Cerris* or *Quercus* (Figure 2). In contrast, oak section explained no significant cytb variation across the whole morphospecies (*p *=* *.49; Table 3a).

Only eight of 112 *M. dorsalis* sp.2 individuals had nuclear genotypes or mitochondrial haplotypes that were inconsistent with expectations based on the oak from which their host galls were sampled (detailed summaries for each individual are provided in Table 4). Incorporating prior information about the oak from which an individual was sampled into our Structure analysis removed one of these apparent conflicts by changing the microsatellite genetic cluster to which individual Mdor1610 was assigned (Table 4). Other cases showed patterns of nuclear and mitochondrial variation that is best explained either by introgression or incomplete lineage sorting between populations associated with the two oak sections. We highlight these examples in turn.

**Table 4:** Summary of ancestry analysis for the eight *Megastigmus dorsalis* cryptic sp.2 individuals showing an inconsistency between their oak‐associated host race classification based upon cytb sequence or initial nuclear microsatellite grouping and the oak section from which they were sampled. The ancestry analysis provides microsatellite groupings revised using prior information on the oak section sampled, and (from left to right) the posterior probabilities that the individual is derived from the host race consistent with its sampling, or that the individual, one of its parents, or one of its grandparents was derived from the alternative host race. The final column provides a conclusion as to the source of the sampling/host race inconsistency

Individual	Gender	Sampled oak section	Cytb host race	Initial microsat host race	Ancestry analysis results					Conclusion
					Sampling‐informed microsat host race	Individual from host race attacking sampled oak	Individual from other host race	Parent from other host race	Grandparent from other host race	
Mdor1610	Male	*Quercus*	*Quercus*	*Cerris*	*Quercus*	0.46	0.08	0.17	0.29	Initial classification using microsat data incorrect
Mdor2368	Male	*Cerris*	*Quercus*	*Cerris*	*Cerris*	0.99	0.00	0.00	0.01	Old introgression or incomplete cytb lineage sorting
Mdor2204	Male	*Quercus*	*Cerris*	*Quercus*	*Quercus*	0.98	0.00	0.00	0.02	Old introgression or incomplete cytb lineage sorting
Mdor1352	Male	*Cerris*	*Quercus*	*Quercus*	*Cerris*	0.40	0.10	0.20	0.30	Initial classification by microsat data incorrect; old introgression or incomplete cytb lineage sorting
Mdor1472	Male	*Quercus*	*Cerris*	*Cerris*	*Quercus*	0.04	0.18	0.37	0.41	Recent introgression; mother/grandmother from *Cerris* host race
Mdor0420	Male	*Quercus*	*Cerris*	*Cerris*	*Quercus*	0.12	0.12	0.24	0.52	Recent introgression; maternal grandmother from *Cerris* host race
Mdor2604	Female	*Quercus*	*Cerris*	*Cerris*	*Cerris*	0.00	1.00	0.00	0.00	Oviposition mistake by mother from *Cerris* host race
Mdor1428	Male	*Quercus*	*Quercus*	*Cerris*	*Cerris*	0.00	0.30	0.59	0.11	Recent introgression; maternal grandfather from *Cerris* host race

1. Three individuals (Mdor2368, Mdor2204, Mdor1352) had a nuclear genotype typical of other samples reared from galls on the same oak section but had cytb haplotypes diagnostic of the second cluster. This nuclear/mitochondrial mismatch could have arisen either through introgression between host races followed by extensive back crossing to the correct host race or incomplete mitochondrial lineage sorting between the two host races. We found both possible nuclear/mitochondrial genotype mismatches (i.e., a nuclear genotype that matches other samples from section *Quercus* combined with a cytb haplotype diagnostic of section *Cerris*, and vice versa), indicating that female dispersal between host oak sections may have occurred in both directions.
2. Individuals Mdor1472 and Mdor0420, both reared from section *Quercus* oaks, showed higher posterior probabilities of recent ancestry from the *Cerris* host race and had *Cerris* host race cytb haplotypes. This is suggestive of introgression events a small number of generations ago resulting from a maternal host shift from host galls on oaks in section *Cerris* to section *Quercus*.
3. Individual Mdor2604 was reared from a host gall on a section *Quercus* oak, despite having a mitochondrial haplotype and nuclear ancestry (with probability of 1.0) derived from the section *Cerris* host race. This pattern is best explained by oviposition on the wrong host by a mother from the section *Cerris* host race.
4. Individual Mdor1428, sampled from section *Quercus*, had high posterior probability of recent nuclear gene ancestry from the *Cerris* host race but a *Quercus* host race cytb haplotype. This pattern is consistent with mating of a male of the section *Cerris* host race with a female of the section *Quercus* host race.

#### 3.1.4 Little evidence of parasitoid population structure associated with host gall location

The plant organ galled by the host gall wasp explained no significant cytb sequence variation within the *M. dorsalis* morphospecies as a whole (*p *=* *.78; Table 3a; Figure 2) but explained significant structure for *M. dorsalis* sp.2 (*p *=* *.03; Table 3a). However, host gall location was associated with no significant genetic structure for the more variable and informative microsatellite data (*p *=* *.14; Table 3b) for *M. dorsalis* sp.2, suggesting that the cytb result for this species may be a false positive (Type 1 error).

### 3.2 Genetic structure supports recruitment of native parasitoids to invading hosts in the UK

**Figure 4:**
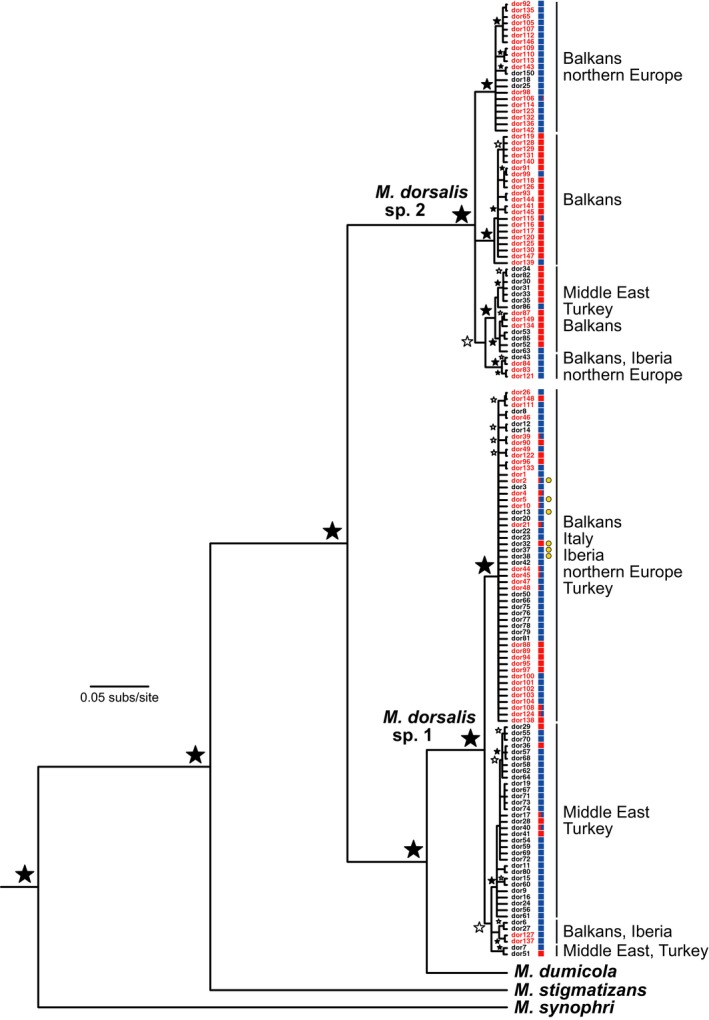
Bayesian 50% majority‐rule consensus tree for the 150 *Megastigmus dorsalis* cytochrome *b* haplotypes obtained in this study and in Nicholls, Preuss, et al. (2010). Clades corresponding to the two cryptic species within the *M. dorsalis* morphospecies are marked, with regions of occurrence indicated for major within‐species clades. Haplotype names in red were sampled from Hungary in this study, while haplotype names in black are from individuals sampled from other regions by Nicholls, Preuss, et al. (2010). Haplotypes of *M. dorsalis* sp.1 occurring in the UK are indicated by yellow circles. Colored squares next to haplotype names indicate the proportion of individuals with that haplotype that were collected from galls on section *Cerris* oaks (red) or section *Quercus* oaks (blue). Black stars next to nodes indicate ≥95% posterior probability node support, white stars indicate node support of ≥70%, other nodes present have posterior support of 50%–69%. Haplotypes are numbered as in Appendix S1

All 52 UK *M. dorsalis* individuals (26 from native host galls and 26 from invading host galls) had cytb sequences diagnostic of *M. dorsalis* sp.1. Our UK sampling revealed six haplotypes, four sampled only in the UK and two that were shared with the Hungarian population (one was common in both locations, the second was very rare in Hungary but sampled at 15 times the frequency in the UK). While the geographic distribution of haplotypes shows little apparent structure within Europe in *M. dorsalis* sp.1 (Figure 4), AMOVAs incorporating haplotype frequencies showed significant differentiation between samples of *M. dorsalis* sp.1 from Hungary and samples from each of native and invading hosts in the UK (*F*_ST_ = 0.036, *p *=* *.022; and *F*_ST_ = 0.028, *p *=* *.048 respectively; see Table 5a,b). In contrast, the UK *M. dorsalis* emerging from native and invading host galls showed no differentiation (*F*_ST_ = −0.010, *p *=* *.612; see Table 5c). These results are compatible with local recruitment of native UK populations onto invading gall hosts but are not predicted by the host pursuit model.

**Table 5:** Partitioning of genetic variation in *Megastigmus dorsalis* cryptic sp.1 associated with native and invading oak gall hosts in the UK using hierarchical analyses of molecular variance of cytochrome *b* haplotype data. (a) differentiation between populations attacking native UK hosts and Hungarian hosts (the source of invading host species); (b) differentiation between populations attacking invading UK hosts and Hungarian hosts; (c) test of genetic structure associated with native or invading host galls within the UK

Taxon assessed	Source of variation	*df*	Variance component	% of total variation	*F*‐statistic	*p*‐value
(a) UK natives and Hungary						
* M. dorsalis* sp.1	UK native vs. Hungary	1	0.011	3.62	*F*_ST_ = 0.036	.022
	Within country	228	0.289	96.38		
(b) UK invaders and Hungary						
* M. dorsalis* sp.1	UK invader vs. Hungary	1	0.008	2.80	*F*_ST_ = 0.028	.048
	Within country	228	0.292	97.20		
(c) Native or invading host galls in the UK						
* M. dorsalis* sp.1	Native vs. invading hosts	1	−0.002	−1.04	*F*_ST_ = −0.010	.612
	Within hosts	50	0.158	101.04		

## 4 DISCUSSION

Our results show genetic structuring within the parasitoid morphospecies *Megastigmus dorsalis* that is associated with host gall wasp traits, revealing previously unknown HAD within this species complex. We have shown that the two cryptic species first described by Nicholls, Preuss, et al. (2010) show differing seasonal phenologies, with *M. dorsalis* sp.2 attacking oak galls primarily in the spring and *M. dorsalis* sp.1 attacking galls primarily in autumn. *Megastigmus dorsalis* sp.2 also contains two divergent genetic lineages that attack galls on different host‐plant taxa, with one host race attacking galls on section *Cerris* oaks and the other attacking galls on section *Quercus* oaks. These results show that some of the same host traits structure both intraspecific genetic diversity and multispecies composition in the oak gall parasitoid community (Bailey et al., 2009), highlighting the importance of tri‐trophic niches in promoting cascades of diversification across interacting trophic levels (Feder & Forbes, 2010; Forbes et al., 2009; Hood et al., 2015; Nyman, Bokma, & Kopelke, 2007; Stireman et al., 2006). Our results also underline the need for more data, even in systems such as the oak gall wasp community that are relatively well known, in order for the subtleties of HAD to be revealed.

### 4.1 Host‐associated structure and speciation within *Megastigmus*

**Figure 5:**
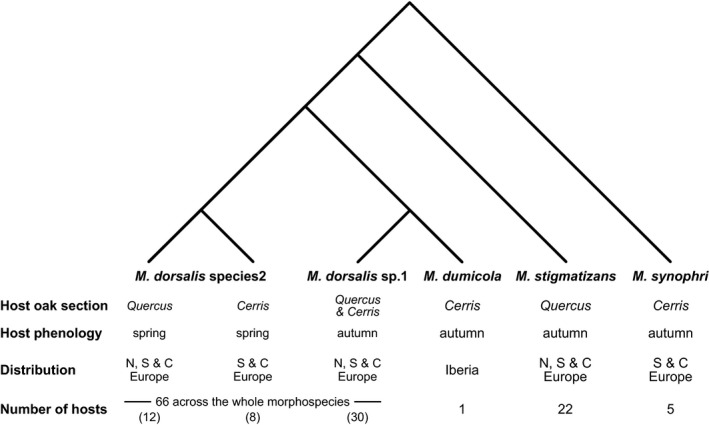
Schematic of the phylogeny of parasitoid *Megastigmus* species in the Western Palaearctic with information on the oak section and phenology of their cynipid host species, their distribution within Europe and the number of hosts they attack. Host numbers for morphospecies are from Askew et al. (2013), Nicholls, Fuentes‐Utrilla, et al. (2010) and Nicholls, Preuss, et al. (2010) based on surveys conducted over many decades; host numbers in parentheses for the cryptic species and host races within *M. dorsalis* are based on the sample of 556 individuals characterized using DNA markers in this study and Nicholls, Preuss, et al. (2010)

**Figure 6:**
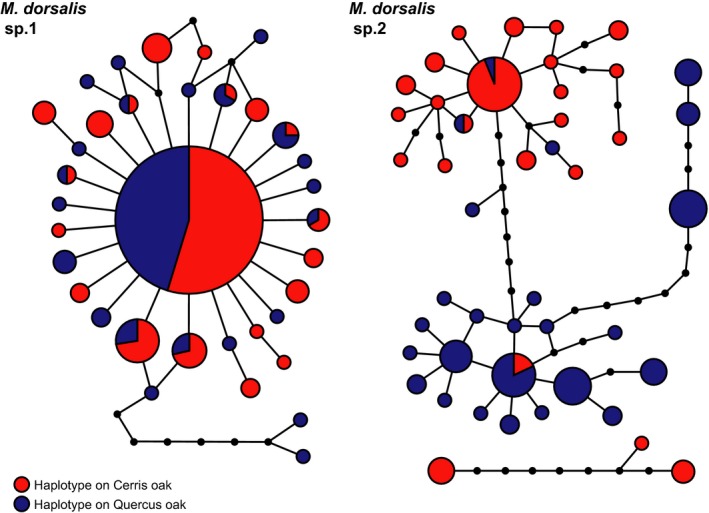
Parsimony networks of cytochrome b haplotypes sampled from the two *Megastigmus dorsalis* cryptic species in Hungary, *M. dorsalis* sp.1 (left) and *M. dorsalis* sp.2 (right). Networks were generated using the software TCS (Clement et al., 2000) using a 95% parsimony cutoff of eight base changes. Each circle represents a unique haplotype, with haplotype frequency proportional to the area of the circle; each line represents a single base change, with unsampled intermediate haplotypes indicated by small black circles. Circles are colored according to the proportion of individuals with that haplotype that were collected from galls on section *Cerris* oaks (red) or section *Quercus* oaks (blue)

Host races are often hypothesized to be a first step toward speciation (Drès & Mallet, 2002; Powell et al., 2013), with host trait diversity creating multiple ecological niches that lead, in higher trophic levels, first to specialized ecotypes and ultimately to reproductively isolated species (Emerson & Kolm, 2005). We hypothesize that the *Megastigmus* species attacking oak galls in the Western Palaearctic illustrate stages along this continuum (Figure 5). *Megastigmus dorsalis* sp.1 is a very polyphagous species that (on the basis of cytb haplotype data at least) shows no host‐associated structuring, attacking many gall species on both oak sections (Figure 2). This species appears to be the more abundant of the two cryptic species, contributing 75% of the individuals sampled in this study and Nicholls, Preuss, et al. (2010) combined. *Megastigmus dorsalis* sp.1 also shows a star‐like haplotype network (Figure 6) consistent with recent population expansion, an interpretation supported by a significant negative value for Tajima's *D* of −2.29 (*p *<* *.001). It is possible that the wide host range of this species may have contributed to its ability both to expand its distribution into northern Europe and to expand its population size following the retreat of the Pleistocene ice sheets.

In contrast, *M. dorsalis* sp.2 is the less abundant of the two cryptic species in our sampling, contributing 25% of samples in this study and Nicholls, Preuss, et al. (2010) combined. It shows high genetic diversity in both cytb haplotypes and microsatellite data, and this is strongly structured at the trophic level of the food plant utilized by its gall wasp hosts. The haplotype network for cytb haplotypes in *M. dorsalis* sp.2 (Figure 6) is not star‐like but shows a more even distribution of haplotypes over individuals within a structured network. Tajima's D for *M. dorsalis* sp.2 shows no significant deviation from zero (Tajima's *D* = −0.22, *p *=* *.48), and no signature of population growth. Host‐associated differentiation in *M. dorsalis* sp.2 parallels that seen in other insects (e.g., Frohlich, Torres‐Jerez, Bedford, Markham, & Brown, 1999; Lozier et al., 2007; Popkin et al., 2017) and is more pronounced than the HAD seen in the classic example of *Rhagoletis* fruit flies and their parasitoids (which is apparent in nuclear allele frequencies, but not in mitochondrial haplotypes; Powell et al., 2013, 2014; Hood et al., 2015). The rarity of gene flow between the two host races (identified in only seven of 112 individuals in our analyses) suggests that *M. dorsalis* sp.2 host races may be in the process of splitting into two daughter species, each specific to a single host oak section. If the oak associations of these two lineages are highly obligate, we expect their geographic distributions to reflect those of the two oak lineages. These predictions are supported by available data (Figure 4). Individuals assigned to the section *Cerris* host race are indeed restricted to the natural range of *Cerris* section oaks in southern and central Europe, Asia Minor, and Iran, while individuals assigned to the section *Quercus* host race have a wider distribution incorporating both southern and northern Europe, mirroring the distribution of its host oak lineage.

The phenological divergence between the two cryptic *M. dorsalis* species parallels that between two cryptic species recently discovered in *Torymus flavipes*, another torymid parasitoid morphospecies attacking oak galls (Kaartinen et al., 2010). Previous work on the *M. dorsalis* morphospecies (summarized in Askew et al., 2013) identified two peaks of adult emergence from oak galls every year and assumed a bivoltine lifecycle for a single species. However, our results suggest that this pattern represents the superposed but contrasting emergence periods of the two cryptic *M. dorsalis* species. Further survey work is now required to assess the extent to which such phenological divergence is characteristic of the two *M. dorsalis* cryptic species in other parts of their distributions.

Three other *Megastigmus* parasitoid species attack Western Palaearctic oak galls: two larger species (*M. stigmatizans*,* M. synophri*) and one species (*M. dumicola*) of similar body size to *M. dorsalis*. Divergence between these species could reflect the final stages of speciation through ecological differentiation (Figure 5). Each attacks hosts associated with just a single oak section, and attacks far fewer hosts than the 66 species attacked by the *M. dorsalis* morphospecies complex (Askew et al., 2013; Nicholls, Fuentes‐Utrilla, et al., 2010; Nicholls, Preuss, et al., 2010). *Megastigmus stigmatizans* is a larger‐bodied species than *M. dorsalis* that parasitizes 22 species of large autumn galls on section *Quercus* oaks. *Megastigmus synophri* is also a larger‐bodied species that parasitizes five species of larger autumn galls only on section *Cerris* oaks. The poorly known Iberian endemic *M. dumicola* has been recorded attacking just a single autumn‐galling gall wasp species on an oak species from the divergent Ilex group within section *Cerris*.

### 4.2 Community‐level implications of host specialization in *M. dorsalis* species

As highlighted by Askew et al. (2013) and mentioned above, all the historical ecological data that have been collected for *M. dorsalis* now need to be re‐assessed, since without molecular identification of the specimens involved the data cannot be reliably attributed to one or other cryptic taxon. While we can make informed guesses as to which species such data may refer to given information on phenology and the oak section of the host gall, the absence of complete separation in these traits between *M. dorsalis* taxa means there will be some error associated with this. As has been highlighted for cryptic *Torymus flavipes* lineages (Kaartinen et al., 2010), many more field collections are required, in conjunction with molecular identification, before a true picture of the ecology of these and similar cryptic parasitoid taxa can be known.

The host traits explored in this analysis were selected based on their demonstrated role in structuring parasitoid communities in oak cynipids (Bailey et al., 2009). A question that follows is whether parasitoid populations could be structured by additional host gall traits, or even across host gall species. While this is possible in principle, achieving the statistical power required to discriminate the effects of larger numbers of gall traits would require more extensive sampling, both of different gall types and of parasitoid individuals from each gall type. We hypothesize that genetic structuring of parasitoid populations at the level of galler species within a given oak/phenology/location phenotype would be unlikely, because the apparently chaotic population dynamics of many host gall wasp populations (Hails & Crawley, 1991; Stone, Schönrogge, Atkinson, Bellido, & Pujade‐Villar, 2002) would make any such genetic structure fleeting at best. The same dynamics are thought to explain why no parasitoids associated with oak cynipid galls are restricted to a single host gall type (Askew et al., 2013), with selection over time having favored a certain degree of polyphagy in response to fluctuating host species abundances, as predicted by theoretical models (Lapchin, 2002).

### 4.3 Lack of HAD and recruitment of native parasitoids to invading herbivore hosts

The differing levels of within‐species HAD observed in this system are relevant to the broader issue of the biocontrol ecosystem service provided by native parasitoids against invading herbivore hosts. The recent invasion of northwestern Europe by oak‐feeding cynipids has provided new sets of host traits for native parasitoids, and of the two cryptic species in the *M. dorsalis* morphospecies*,* only native populations of the more generalist and less structured species 1, attacking autumn galls, significantly exploit these hosts in the UK. Other parasitoids have also recruited rapidly to invading gall wasps in the UK (Schönrogge et al., 1995, 2000, 2012), and it remains to be seen whether data for these species are more compatible with either the local recruitment or host pursuit models of community assembly. Recruitment of native parasitoids can contribute significantly to mortality imposed on economically important herbivore pests, and we might predict that more generalist and less host‐structured species would be more effective in this role. Support for this hypothesis comes from recruitment of parasitoids that normally attack oak cynipid gall wasp hosts to the galls of the invasive oriental chestnut gall wasp, *Dryocosmus kuriphilus*, an economically important pest of *Castanea sativa. Megastigmus dorsalis* sp.1 recruited to this host at multiple locations within Europe within only a few years of this pest's introduction from China (Aebi et al., 2007; Matoševic & Melika, 2013; Quacchia et al., 2013), providing some level of natural biocontrol. In contrast, although *M. dorsalis* sp.2 is recorded as attacking *Dryocosmus kuriphilus*, it is so far only rarely recorded from this host in a single location (Quacchia et al., 2013).

## DATA ACCESSIBILITY

Cytb DNA sequences are available from GenBank (accessions GU123486‐GU123573 and KX980093‐KX980153). Sampling information, host association data, and microsatellite genotypes are available for each individual as online appendices.

## CONFLICT OF INTEREST

None declared.

## AUTHOR CONTRIBUTIONS

JAN, KS, and GNS devised the study. JAN designed the sampling, collected, and analyzed the data. JAN and GNS wrote the manuscript. SP helped collect samples and generated the microsatellite data.
