# Supplementary material for: Partitioning of herbivore hosts across time and food plants promotes diversification in the Megastigmus dorsalis oak gall parasitoid complex
Source: Ecol Evol. 2017 Dec 25;8(2):1300–15. doi: 10.1002/ece3.3712 (PMC5773290; doi:10.1002/ece3.3712)
Supplement: Supplementary file 1 [file ECE3-8-1300-s001.doc]

Appendix 1. Metadata for *Megastigmus* samples sequenced for cytochrome *b*, including collection information, host gall and host plant characteristics and genetic species assignments. Autumn galls correspond to host asexual generations, and spring galls correspond to host sexual generations. Numbers next to Hungarian site names correspond to numerical labels in Figure 1. All *M. dorsalis* sp. 2 individuals were also genotyped at eight microsatellite loci. Rows at the bottom of the table below the line provide host oak information for samples from throughout the Western Palaearctic that were sequenced by Nicholls *et al*. 2010b.

| **sample name** | **sex** | **host gall** | **host gall clade** | **oak section** | **season** | **plant organ**  **galled** | **UK galler status** | **country** | **site** | **latitude** | **longitude** | **Hungarian region** | **collection date** | **cytb haplotype** | **Cryptic species assignment** |
| --- | --- | --- | --- | --- | --- | --- | --- | --- | --- | --- | --- | --- | --- | --- | --- |
| d10 |  | *Andricus grossulariae* | lucidus | quercus | autumn | catkin |  | Hungary | 17. Gödöllö | 47.6 | 19.33 | region1 | Sep-2001 | dor2 | *M. dorsalis* sp.1 |
| d11 |  | *Andricus grossulariae* | lucidus | quercus | autumn | catkin |  | Hungary | 17. Gödöllö | 47.6 | 19.33 | region1 | Sep-2001 | dor2 | *M. dorsalis* sp.1 |
| d12 |  | *Andricus hungaricus* | quercuscalicis | quercus | autumn | bud |  | Hungary | 17. Gödöllö | 47.6 | 19.33 | region1 | Oct-2001 | dor1 | *M. dorsalis* sp.1 |
| d13 |  | *Andricus lignicolus* | kollari | quercus | autumn | bud |  | Hungary | 17. Gödöllö | 47.6 | 19.33 | region1 | Oct-2001 | dor2 | *M. dorsalis* sp.1 |
| d23 |  | *Andricus coriarius* | kollari | quercus | autumn | bud |  | Hungary | 14. Tatabanya | 47.55 | 18.45 |  | Nov-1999 | dor4 | *M. dorsalis* sp.1 |
| d24 |  | *Andricus coriarius* | kollari | quercus | autumn | bud |  | Hungary | 14. Tatabanya | 47.55 | 18.45 |  | Nov-1999 | dor4 | *M. dorsalis* sp.1 |
| Mdor0145 | f | *Andricus quercustozae* | quercuscalicis | quercus | autumn | bud |  | Hungary | 10. Veszprém | 47.1 | 17.9 | region3 | Nov-1999 | dor5 | *M. dorsalis* sp.1 |
| Mdor0343 | m | *Andricus quercustozae* | quercuscalicis | quercus | autumn | bud |  | Hungary | 27. Szölöske | 47.92 | 20.45 |  | Nov-1999 | dor2 | *M. dorsalis* sp.1 |
| Mdor0355 | f | *Andricus grossulariae* | lucidus | quercus | autumn | catkin |  | Hungary | 17. Gödöllö | 47.6 | 19.33 | region1 | Sep-2001 | dor103 | *M. dorsalis* sp.1 |
| Mdor0356 | f | *Andricus grossulariae* | lucidus | quercus | autumn | catkin |  | Hungary | 17. Gödöllö | 47.6 | 19.33 | region1 | Oct-2001 | dor2 | *M. dorsalis* sp.1 |
| Mdor0359 | f | *Andricus grossulariae* | lucidus | quercus | autumn | catkin |  | Hungary | 17. Gödöllö | 47.6 | 19.33 | region1 | Sep-2001 | dor102 | *M. dorsalis* sp.1 |
| Mdor0363 | f | *Andricus coriarius* | kollari | quercus | autumn | bud |  | Hungary | 25. Szentkút | 48 | 19.77 |  | Oct-2001 | dor108 | *M. dorsalis* sp.1 |
| Mdor0365 | f | *Andricus lignicolus* | kollari | quercus | autumn | bud |  | Hungary | 17. Gödöllö | 47.6 | 19.33 | region1 | Oct-2001 | dor111 | *M. dorsalis* sp.1 |
| Mdor0366 | f | *Andricus lignicolus* | kollari | quercus | autumn | bud |  | Hungary | 22. Mátrafüred | 47.83 | 19.97 | region2 | Oct-2001 | dor45 | *M. dorsalis* sp.1 |
| Mdor0369 | f | *Andricus lignicolus* | kollari | quercus | autumn | bud |  | Hungary | 17. Gödöllö | 47.6 | 19.33 | region1 | Oct-2001 | dor2 | *M. dorsalis* sp.1 |
| Mdor0371 | m | *Andricus kollari* | kollari | quercus | autumn | bud |  | Hungary | 17. Gödöllö | 47.6 | 19.33 | region1 | Aug-2001 | dor2 | *M. dorsalis* sp.1 |
| Mdor0372 | f | *Andricus coriarius* | kollari | quercus | autumn | bud |  | Hungary | 17. Gödöllö | 47.6 | 19.33 | region1 | Sep-2000 | dor2 | *M. dorsalis* sp.1 |
| Mdor0374 | f | *Pseudoneuroterus saliens* | Pseudoneuroterus | cerris | spring | acorn |  | Hungary | 17. Gödöllö | 47.6 | 19.33 | region1 | May-2001 | dor119 | *M. dorsalis* sp.2 |
| Mdor0378 | f | *Andricus kollari* | kollari | quercus | autumn | bud |  | Hungary | 17. Gödöllö | 47.6 | 19.33 | region1 | Sep-2000 | dor2 | *M. dorsalis* sp.1 |
| Mdor0379 | f | *Callirhytis glandium* | Callirhytis | cerris | autumn | acorn |  | Hungary | 22. Mátrafüred | 47.83 | 19.97 | region2 | Oct-2001 | dor2 | *M. dorsalis* sp.1 |
| Mdor0380 | m | *Callirhytis glandium* | Callirhytis | cerris | autumn | acorn |  | Hungary | 22. Mátrafüred | 47.83 | 19.97 | region2 | Oct-2001 | dor2 | *M. dorsalis* sp.1 |
| Mdor0383 | f | *Cynips quercusfolii* | Cynips | quercus | autumn | leaf |  | Hungary | 17. Gödöllö | 47.6 | 19.33 | region1 | Sep-2000 | dor2 | *M. dorsalis* sp.1 |
| Mdor0392 | m | *Callirhytis glandium* | Callirhytis | cerris | autumn | acorn |  | Hungary | 22. Mátrafüred | 47.83 | 19.97 | region2 | Oct-2001 | dor2 | *M. dorsalis* sp.1 |
| Mdor0394 | f | *Callirhytis glandium* | Callirhytis | cerris | autumn | acorn |  | Hungary | 22. Mátrafüred | 47.83 | 19.97 | region2 | Oct-2001 | dor4 | *M. dorsalis* sp.1 |
| Mdor0396 | f | *Andricus grossulariae* | lucidus | quercus | autumn | catkin |  | Hungary | 17. Gödöllö | 47.6 | 19.33 | region1 | Sep-2000 | dor101 | *M. dorsalis* sp.1 |
| Mdor0403 | f | *Andricus coriarius* | kollari | quercus | autumn | bud |  | Hungary | 17. Gödöllö | 47.6 | 19.33 | region1 | Sep-2000 | dor2 | *M. dorsalis* sp.1 |
| Mdor0407 | m | *Andricus kollari* | kollari | quercus | autumn | bud |  | Hungary | 17. Gödöllö | 47.6 | 19.33 | region1 | Oct-2000 | dor2 | *M. dorsalis* sp.1 |
| Mdor0408 | f | *Callirhytis glandium* | Callirhytis | cerris | autumn | acorn |  | Hungary | 22. Mátrafüred | 47.83 | 19.97 | region2 | Oct-2001 | dor2 | *M. dorsalis* sp.1 |
| Mdor0410 | f | *Callirhytis glandium* | Callirhytis | cerris | autumn | acorn |  | Hungary | 22. Mátrafüred | 47.83 | 19.97 | region2 | Oct-2001 | dor10 | *M. dorsalis* sp.1 |
| Mdor0411 | f | *Andricus kollari* | kollari | quercus | autumn | bud |  | Hungary | 17. Gödöllö | 47.6 | 19.33 | region1 | Sep-2001 | dor2 | *M. dorsalis* sp.1 |
| Mdor0412 | m | *Callirhytis glandium* | Callirhytis | cerris | autumn | acorn |  | Hungary | 22. Mátrafüred | 47.83 | 19.97 | region2 | Oct-2001 | dor2 | *M. dorsalis* sp.1 |
| Mdor0413 | f | *Pseudoneuroterus saliens* | Pseudoneuroterus | cerris | spring | acorn |  | Hungary | 22. Mátrafüred | 47.83 | 19.97 | region2 | May-2001 | dor115 | *M. dorsalis* sp.2 |
| Mdor0416 | m | *Andricus glutinosus* | quercuscalicis | quercus | autumn | bud |  | Hungary | 22. Mátrafüred | 47.83 | 19.97 | region2 | Oct-2001 | dor2 | *M. dorsalis* sp.1 |
| Mdor0417 | f | *Callirhytis glandium* | Callirhytis | cerris | autumn | acorn |  | Hungary | 22. Mátrafüred | 47.83 | 19.97 | region2 | Oct-2001 | dor89 | *M. dorsalis* sp.1 |
| Mdor0418 | m | *Callirhytis glandium* | Callirhytis | cerris | autumn | acorn |  | Hungary | 22. Mátrafüred | 47.83 | 19.97 | region2 | Oct-2001 | dor89 | *M. dorsalis* sp.1 |
| Mdor0420 | m | *Andricus testaceipes* | inflator | quercus | spring | shoot |  | Hungary | 22. Mátrafüred | 47.83 | 19.97 | region2 | Jul-2001 | dor99 | *M. dorsalis* sp.2 |
| Mdor0421 | m | *Andricus caputmedusae* | quercuscalicis | quercus | autumn | acorn |  | Hungary | 22. Mátrafüred | 47.83 | 19.97 | region2 | Sep-2001 | dor48 | *M. dorsalis* sp.1 |
| Mdor0422 | f | *Callirhytis glandium* | Callirhytis | cerris | autumn | acorn |  | Hungary | 22. Mátrafüred | 47.83 | 19.97 | region2 | Oct-2001 | dor2 | *M. dorsalis* sp.1 |
| Mdor0425 | f | *Andricus grossulariae* | lucidus | cerris | spring | catkin |  | Hungary | 17. Gödöllö | 47.6 | 19.33 | region1 | May-2001 | dor2 | *M. dorsalis* sp.1 |
| Mdor0427 | m | *Andricus testaceipes* | inflator | quercus | spring | shoot |  | Hungary | 22. Mátrafüred | 47.83 | 19.97 | region2 | Jul-2001 | dor106 | *M. dorsalis* sp.2 |
| Mdor0429 | f | *Andricus galeatus* | kollari | quercus | autumn | bud |  | Hungary | 17. Gödöllö | 47.6 | 19.33 | region1 | Aug-2001 | dor2 | *M. dorsalis* sp.1 |
| Mdor0431 | m | *Callirhytis glandium* | Callirhytis | cerris | autumn | acorn |  | Hungary | 22. Mátrafüred | 47.83 | 19.97 | region2 | Oct-2001 | dor2 | *M. dorsalis* sp.1 |
| Mdor0432 | f | *Callirhytis glandium* | Callirhytis | cerris | autumn | acorn |  | Hungary | 22. Mátrafüred | 47.83 | 19.97 | region2 | Oct-2001 | dor2 | *M. dorsalis* sp.1 |
| Mdor0438 | f | *Synophrus politus* | Synophrus | cerris | autumn | shoot |  | Hungary | 25. Szentkút | 48 | 19.77 |  | Aug-2001 | dor2 | *M. dorsalis* sp.1 |
| Mdor0439 | m | *Andricus coronatus* | quercuscalicis | quercus | autumn | bud |  | Hungary | 25. Szentkút | 48 | 19.77 |  | Oct-2001 | dor2 | *M. dorsalis* sp.1 |
| Mdor0440 | f | *Synophrus politus* | Synophrus | cerris | autumn | shoot |  | Hungary | 25. Szentkút | 48 | 19.77 |  | Aug-2001 | dor2 | *M. dorsalis* sp.1 |
| Mdor0443 | f | *Andricus lucidus* | lucidus | quercus | autumn | bud |  | Hungary | 22. Mátrafüred | 47.83 | 19.97 | region2 | Sep-2001 | dor46 | *M. dorsalis* sp.1 |
| Mdor0448 | f | *Callirhytis glandium* | Callirhytis | cerris | autumn | acorn |  | Hungary | 22. Mátrafüred | 47.83 | 19.97 | region2 | Oct-2001 | dor44 | *M. dorsalis* sp.1 |
| Mdor0449 | f | *Andricus grossulariae* | lucidus | quercus | autumn | catkin |  | Hungary | 17. Gödöllö | 47.6 | 19.33 | region1 | Sep-2001 | dor2 | *M. dorsalis* sp.1 |
| Mdor0450 | f | *Andricus foecundatrix* | foecundatrix | quercus | autumn | bud |  | Hungary | 17. Gödöllö | 47.6 | 19.33 | region1 | Jul-2001 | dor83 | *M. dorsalis* sp.2 |
| Mdor0453 | f | *Callirhytis glandium* | Callirhytis | cerris | autumn | acorn |  | Hungary | 22. Mátrafüred | 47.83 | 19.97 | region2 | Oct-2001 | dor88 | *M. dorsalis* sp.1 |
| Mdor0454 | f | *Callirhytis glandium* | Callirhytis | cerris | autumn | acorn |  | Hungary | 22. Mátrafüred | 47.83 | 19.97 | region2 | Oct-2001 | dor2 | *M. dorsalis* sp.1 |
| Mdor0456 | f | *Andricus lignicolus* | kollari | quercus | autumn | bud |  | Hungary | 22. Mátrafüred | 47.83 | 19.97 | region2 | Oct-2001 | dor2 | *M. dorsalis* sp.1 |
| Mdor0457 | f | *Andricus lucidus* | lucidus | quercus | autumn | bud |  | Hungary | 17. Gödöllö | 47.6 | 19.33 | region1 | Oct-2001 | dor45 | *M. dorsalis* sp.1 |
| Mdor0461 | f | *Andricus lucidus* | lucidus | quercus | autumn | bud |  | Hungary | 22. Mátrafüred | 47.83 | 19.97 | region2 | Sep-2001 | dor47 | *M. dorsalis* sp.1 |
| Mdor0470 | m | *Andricus hungaricus* | quercuscalicis | quercus | autumn | bud |  | Hungary | 17. Gödöllö | 47.6 | 19.33 | region1 | Oct-2001 | dor2 | *M. dorsalis* sp.1 |
| Mdor0478 | f | *Andricus lucidus* | lucidus | quercus | autumn | bud |  | Hungary | 17. Gödöllö | 47.6 | 19.33 | region1 | Oct-2001 | dor48 | *M. dorsalis* sp.1 |
| Mdor0492 | m | *Andricus kollari* | kollari | quercus | autumn | bud |  | Hungary | 17. Gödöllö | 47.6 | 19.33 | region1 | Sep-2000 | dor4 | *M. dorsalis* sp.1 |
| Mdor0497 | f | *Andricus lignicolus* | kollari | quercus | autumn | bud |  | Hungary | 17. Gödöllö | 47.6 | 19.33 | region1 | Oct-2001 | dor49 | *M. dorsalis* sp.1 |
| Mdor0499 | f | *Andricus lucidus* | lucidus | quercus | autumn | bud |  | Hungary | 3. Sopron | 47.68 | 16.6 | region4 | Oct-2001 | dor2 | *M. dorsalis* sp.1 |
| Mdor0509 | m | *Andricus grossulariae* | lucidus | quercus | autumn | catkin |  | Hungary | 17. Gödöllö | 47.6 | 19.33 | region1 | Oct-2000 | dor2 | *M. dorsalis* sp.1 |
| Mdor0515 | m | *Callirhytis glandium* | Callirhytis | cerris | autumn | acorn |  | Hungary | 22. Mátrafüred | 47.83 | 19.97 | region2 | Oct-2001 | dor94 | *M. dorsalis* sp.1 |
| Mdor0527 | f | *Andricus lucidus* | lucidus | quercus | autumn | bud |  | Hungary | 22. Mátrafüred | 47.83 | 19.97 | region2 | Sep-2001 | dor2 | *M. dorsalis* sp.1 |
| Mdor0536 | f | *Andricus lignicolus* | kollari | quercus | autumn | bud |  | Hungary | 17. Gödöllö | 47.6 | 19.33 | region1 | Oct-2001 | dor26 | *M. dorsalis* sp.1 |
| Mdor0538 | f | *Andricus lucidus* | lucidus | quercus | autumn | bud |  | Hungary | 22. Mátrafüred | 47.83 | 19.97 | region2 | Sep-2001 | dor2 | *M. dorsalis* sp.1 |
| Mdor0539 | f | *Andricus lucidus* | lucidus | quercus | autumn | bud |  | Hungary | 17. Gödöllö | 47.6 | 19.33 | region1 | Oct-2001 | dor100 | *M. dorsalis* sp.1 |
| Mdor0547 | m | *Andricus quercustozae* | quercuscalicis | quercus | autumn | bud |  | Hungary | 27. Szölöske | 47.92 | 20.45 |  | Nov-1999 | dor2 | *M. dorsalis* sp.1 |
| Mdor1249 | m | *Andricus quercustozae* | quercuscalicis | quercus | autumn | bud |  | Hungary | 10. Veszprém | 47.1 | 17.9 | region3 | Nov-1999 | dor2 | *M. dorsalis* sp.1 |
| Mdor1250 | m | *Andricus quercustozae* | quercuscalicis | quercus | autumn | bud |  | Hungary | 27. Szölöske | 47.92 | 20.45 |  | Nov-1999 | dor2 | *M. dorsalis* sp.1 |
| Mdor1251 | f | *Andricus lucidus* | lucidus | quercus | autumn | bud |  | Hungary | 22. Mátrafüred | 47.83 | 19.97 | region2 | Sep-2001 | dor46 | *M. dorsalis* sp.1 |
| Mdor1254 | f | *Andricus coriarius* | kollari | quercus | autumn | bud |  | Hungary | 11. Várpalota | 47.2 | 18.21 | region3 | Nov-1999 | dor2 | *M. dorsalis* sp.1 |
| Mdor1255 | f | *Andricus coriarius* | kollari | quercus | autumn | bud |  | Hungary | 11. Várpalota | 47.2 | 18.21 | region3 | Nov-1999 | dor2 | *M. dorsalis* sp.1 |
| Mdor1256 | f | *Andricus coriarius* | kollari | quercus | autumn | bud |  | Hungary | 11. Várpalota | 47.2 | 18.21 | region3 | Nov-1999 | dor2 | *M. dorsalis* sp.1 |
| Mdor1284 | f | *Andricus conglomeratus* | kollari | quercus | autumn | bud |  | Hungary | 16. Kemence | 48.02 | 18.89 |  | Nov-1999 | dor2 | *M. dorsalis* sp.1 |
| Mdor1288 | f | *Andricus conglomeratus* | kollari | quercus | autumn | bud |  | Hungary | 27. Szölöske | 47.92 | 20.45 |  | Nov-1999 | dor2 | *M. dorsalis* sp.1 |
| Mdor1289 | f | *Andricus conglomeratus* | kollari | quercus | autumn | bud |  | Hungary | 27. Szölöske | 47.92 | 20.45 |  | Nov-1999 | dor2 | *M. dorsalis* sp.1 |
| Mdor1294 | f | *Andricus conglomeratus* | kollari | quercus | autumn | bud |  | Hungary | 26. Sirok | 47.94 | 20.25 |  | Nov-1999 | dor127 | *M. dorsalis* sp.1 |
| Mdor1295 | f | *Andricus conglomeratus* | kollari | quercus | autumn | bud |  | Hungary | 26. Sirok | 47.94 | 20.25 |  | Nov-1999 | dor2 | *M. dorsalis* sp.1 |
| Mdor1346 | f | *Pseudoneuroterus macropterus* | Pseudoneuroterus | cerris | autumn | shoot |  | Hungary | 13. Pécsvárad | 46.15 | 18.42 |  | Dec-2005 | dor87 | *M. dorsalis* sp.2 |
| Mdor1347 | f | *Pseudoneuroterus macropterus* | Pseudoneuroterus | cerris | autumn | shoot |  | Hungary | 13. Pécsvárad | 46.15 | 18.42 |  | Dec-2005 | dor87 | *M. dorsalis* sp.2 |
| Mdor1350 | m | *Aphelonyx cerricola* | Aphelonyx | cerris | autumn | bud |  | Hungary | 18. Isaszeg | 47.53 | 19.4 | region1 | Sep-2005 | dor2 | *M. dorsalis* sp.1 |
| Mdor1352 | m | *Synophrus politus* | Synophrus | cerris | autumn | shoot |  | Hungary | 18. Isaszeg | 47.53 | 19.4 | region1 | Sep-2006 | dor106 | *M. dorsalis* sp.2 |
| Mdor1363 | f | *Pseudoneuroterus macropterus* | Pseudoneuroterus | cerris | autumn | shoot |  | Hungary | 21. Gyöngyössolymos | 47.82 | 19.93 | region2 | Apr-2006 | dor39 | *M. dorsalis* sp.1 |
| Mdor1397 | m | *Andricus infectorius* | kollari | quercus | autumn | bud |  | Hungary | 22. Mátrafüred | 47.83 | 19.97 | region2 | Sep-2005 | dor2 | *M. dorsalis* sp.1 |
| Mdor1407 | f | *Andricus infectorius* | kollari | quercus | autumn | bud |  | Hungary | 22. Mátrafüred | 47.83 | 19.97 | region2 | Oct-2004 | dor132 | *M. dorsalis* sp.2 |
| Mdor1409 | f | *Andricus glutinosus* | quercuscalicis | quercus | autumn | bud |  | Hungary | 22. Mátrafüred | 47.83 | 19.97 | region2 | Oct-2004 | dor65 | *M. dorsalis* sp.2 |
| Mdor1417 | m | *Andricus kollari* | kollari | quercus | autumn | bud |  | Hungary | 18. Isaszeg | 47.53 | 19.4 | region1 | Oct-2004 | dor101 | *M. dorsalis* sp.1 |
| Mdor1418 | m | *Andricus quercuscalicis* | quercuscalicis | quercus | autumn | acorn |  | Hungary | 19. Jászbereny | 47.5 | 19.93 |  | Oct-2004 | dor84 | *M. dorsalis* sp.2 |
| Mdor1419 | m | *Aphelonyx cerricola* | Aphelonyx | cerris | autumn | bud |  | Hungary | 3. Sopron | 47.68 | 16.6 | region4 | Nov-1999 | dor2 | *M. dorsalis* sp.1 |
| Mdor1428 | m | *Andricus glutinosus* | quercuscalicis | quercus | autumn | bud |  | Hungary | 22. Mátrafüred | 47.83 | 19.97 | region2 | Oct-2004 | dor107 | *M. dorsalis* sp.2 |
| Mdor1444 | f | *Aphelonyx cerricola* | Aphelonyx | cerris | autumn | bud |  | Hungary | 18. Isaszeg | 47.53 | 19.4 | region1 | Sep-2005 | dor2 | *M. dorsalis* sp.1 |
| Mdor1445 | f | *Aphelonyx cerricola* | Aphelonyx | cerris | autumn | bud |  | Hungary | 3. Sopron | 47.68 | 16.6 | region4 | Sep-2005 | dor2 | *M. dorsalis* sp.1 |
| Mdor1446 | f | *Aphelonyx cerricola* | Aphelonyx | cerris | autumn | bud |  | Hungary | 3. Sopron | 47.68 | 16.6 | region4 | Nov-1999 | dor2 | *M. dorsalis* sp.1 |
| Mdor1448 | m | *Aphelonyx cerricola* | Aphelonyx | cerris | autumn | bud |  | Hungary | 17. Gödöllö | 47.6 | 19.33 | region1 | Aug-2001 | dor2 | *M. dorsalis* sp.1 |
| Mdor1471 | f | *Andricus hungaricus* | quercuscalicis | quercus | autumn | bud |  | Hungary | 8. Lövő Hills | 47.51 | 16.78 | region4 | Oct-2001 | dor110 | *M. dorsalis* sp.2 |
| Mdor1472 | m | *Andricus hungaricus* | quercuscalicis | quercus | autumn | bud |  | Hungary | 17. Gödöllö | 47.6 | 19.33 | region1 | Oct-2001 | dor120 | *M. dorsalis* sp.2 |
| Mdor1480 | f | *Andricus lucidus* | lucidus | quercus | autumn | bud |  | Hungary | 19. Jászbereny | 47.5 | 19.93 |  | Oct-2004 | dor124 | *M. dorsalis* sp.1 |
| Mdor1487 | m | *Andricus glutinosus* | quercuscalicis | quercus | autumn | bud |  | Hungary | 22. Mátrafüred | 47.83 | 19.97 | region2 | Oct-2004 | dor110 | *M. dorsalis* sp.2 |
| Mdor1489 | f | *Andricus quercuscalicis* | quercuscalicis | quercus | autumn | acorn |  | Hungary | 19. Jászbereny | 47.5 | 19.93 |  | Oct-2004 | dor106 | *M. dorsalis* sp.2 |
| Mdor1493 | f | *Aphelonyx cerricola* | Aphelonyx | cerris | autumn | bud |  | Hungary | 17. Gödöllö | 47.6 | 19.33 | region1 | Aug-2001 | dor2 | *M. dorsalis* sp.1 |
| Mdor1494 | f | *Pseudoneuroterus macropterus* | Pseudoneuroterus | cerris | autumn | shoot |  | Hungary | 16. Kemence | 48.02 | 18.89 |  | Nov-1999 | dor108 | *M. dorsalis* sp.1 |
| Mdor1495 | f | *Pseudoneuroterus macropterus* | Pseudoneuroterus | cerris | autumn | shoot |  | Hungary | 3. Sopron | 47.68 | 16.6 | region4 | Nov-1999 | dor2 | *M. dorsalis* sp.1 |
| Mdor1496 | m | *Pseudoneuroterus macropterus* | Pseudoneuroterus | cerris | autumn | shoot |  | Hungary | 3. Sopron | 47.68 | 16.6 | region4 | Nov-1999 | dor2 | *M. dorsalis* sp.1 |
| Mdor1498 | f | *Andricus coronatus* | quercuscalicis | quercus | autumn | bud |  | Hungary | 25. Szentkút | 48 | 19.77 |  | Oct-2001 | dor2 | *M. dorsalis* sp.1 |
| Mdor1499 | f | *Andricus glutinosus* | quercuscalicis | quercus | autumn | bud |  | Hungary | 22. Mátrafüred | 47.83 | 19.97 | region2 | Oct-2004 | dor105 | *M. dorsalis* sp.2 |
| Mdor1500 | f | *Andricus hungaricus* | quercuscalicis | quercus | autumn | bud |  | Hungary | 17. Gödöllö | 47.6 | 19.33 | region1 | Oct-2001 | dor104 | *M. dorsalis* sp.1 |
| Mdor1501 | m | *Pseudoneuroterus macropterus* | Pseudoneuroterus | cerris | autumn | shoot |  | Hungary | 3. Sopron | 47.68 | 16.6 | region4 | Nov-1999 | dor2 | *M. dorsalis* sp.1 |
| Mdor1503 | f | *Synophrus politus* | Synophrus | cerris | autumn | shoot |  | Hungary | 13. Pécsvárad | 46.15 | 18.42 |  | Feb-2006 | dor2 | *M. dorsalis* sp.1 |
| Mdor1505 | f | *Andricus multiplicatus* | multiplicatus | cerris | spring | leaf |  | Hungary | 11. Várpalota | 47.2 | 18.21 | region3 | Jun-2006 | dor97 | *M. dorsalis* sp.1 |
| Mdor1506 | m | *Andricus multiplicatus* | multiplicatus | cerris | spring | leaf |  | Hungary | 11. Várpalota | 47.2 | 18.21 | region3 | Jun-2006 | dor97 | *M. dorsalis* sp.1 |
| Mdor1508 | f | *Andricus multiplicatus* | multiplicatus | cerris | spring | leaf |  | Hungary | 11. Várpalota | 47.2 | 18.21 | region3 | Jun-2006 | dor2 | *M. dorsalis* sp.1 |
| Mdor1509 | m | *Andricus multiplicatus* | multiplicatus | cerris | spring | leaf |  | Hungary | 11. Várpalota | 47.2 | 18.21 | region3 | Jun-2006 | dor96 | *M. dorsalis* sp.1 |
| Mdor1510 | m | *Andricus multiplicatus* | multiplicatus | cerris | spring | leaf |  | Hungary | 11. Várpalota | 47.2 | 18.21 | region3 | Jun-2006 | dor2 | *M. dorsalis* sp.1 |
| Mdor1512 | m | *Andricus multiplicatus* | multiplicatus | cerris | spring | leaf |  | Hungary | 11. Várpalota | 47.2 | 18.21 | region3 | Jun-2006 | dor95 | *M. dorsalis* sp.1 |
| Mdor1514 | m | *Andricus multiplicatus* | multiplicatus | cerris | spring | leaf |  | Hungary | 11. Várpalota | 47.2 | 18.21 | region3 | Jun-2006 | dor2 | *M. dorsalis* sp.1 |
| Mdor1515 | f | *Andricus multiplicatus* | multiplicatus | cerris | spring | leaf |  | Hungary | 11. Várpalota | 47.2 | 18.21 | region3 | Jun-2006 | dor95 | *M. dorsalis* sp.1 |
| Mdor1517 | f | *Andricus multiplicatus* | multiplicatus | cerris | spring | leaf |  | Hungary | 11. Várpalota | 47.2 | 18.21 | region3 | Jun-2006 | dor2 | *M. dorsalis* sp.1 |
| Mdor1519 | m | *Andricus multiplicatus* | multiplicatus | cerris | spring | leaf |  | Hungary | 11. Várpalota | 47.2 | 18.21 | region3 | Jun-2006 | dor94 | *M. dorsalis* sp.1 |
| Mdor1520 | m | *Andricus multiplicatus* | multiplicatus | cerris | spring | leaf |  | Hungary | 11. Várpalota | 47.2 | 18.21 | region3 | Jun-2006 | dor2 | *M. dorsalis* sp.1 |
| Mdor1521 | m | *Andricus multiplicatus* | multiplicatus | cerris | spring | leaf |  | Hungary | 11. Várpalota | 47.2 | 18.21 | region3 | Jun-2006 | dor2 | *M. dorsalis* sp.1 |
| Mdor1522 | m | *Andricus multiplicatus* | multiplicatus | cerris | spring | leaf |  | Hungary | 11. Várpalota | 47.2 | 18.21 | region3 | Jun-2006 | dor2 | *M. dorsalis* sp.1 |
| Mdor1523 | f | *Andricus multiplicatus* | multiplicatus | cerris | spring | leaf |  | Hungary | 11. Várpalota | 47.2 | 18.21 | region3 | Jun-2006 | dor93 | *M. dorsalis* sp.2 |
| Mdor1525 | f | *Biorhiza pallida* | Biorhiza | quercus | spring | bud |  | Hungary | 24. Mátrahaza | 47.87 | 19.98 | region2 | Jun-2006 | dor84 | *M. dorsalis* sp.2 |
| Mdor1526 | m | *Biorhiza pallida* | Biorhiza | quercus | spring | bud |  | Austria | 5. Ober Pullendorf | 47.52 | 16.51 | region4 | Jun-2006 | dor114 | *M. dorsalis* sp.2 |
| Mdor1556 | m | *Pseudoneuroterus macropterus* | Pseudoneuroterus | cerris | autumn | shoot |  | Hungary | 21. Gyöngyössolymos | 47.82 | 19.93 | region2 | Apr-2006 | dor120 | *M. dorsalis* sp.2 |
| Mdor1557 | m | *Pseudoneuroterus macropterus* | Pseudoneuroterus | cerris | autumn | shoot |  | Hungary | 1. Fertörákos | 47.72 | 16.65 | region4 | Jan-2006 | dor118 | *M. dorsalis* sp.2 |
| Mdor1559 | f | *Pseudoneuroterus macropterus* | Pseudoneuroterus | cerris | autumn | shoot |  | Hungary | 1. Fertörákos | 47.72 | 16.65 | region4 | Feb-2006 | dor117 | *M. dorsalis* sp.2 |
| Mdor1563 | f | *Andricus lucidus* | lucidus | quercus | autumn | bud |  | Hungary | 13. Pécsvárad | 46.15 | 18.42 |  | Feb-2006 | dor133 | *M. dorsalis* sp.1 |
| Mdor1567 | f | *Andricus lucidus* | lucidus | quercus | autumn | bud |  | Hungary | 23. Sástó | 47.84 | 19.96 | region2 | Feb-2006 | dor2 | *M. dorsalis* sp.1 |
| Mdor1569 | f | *Pseudoneuroterus macropterus* | Pseudoneuroterus | cerris | autumn | shoot |  | Hungary | 22. Mátrafüred | 47.83 | 19.97 | region2 | Jan-2006 | dor125 | *M. dorsalis* sp.2 |
| Mdor1587 | m | *Aphelonyx cerricola* | Aphelonyx | cerris | autumn | bud |  | Hungary | 3. Sopron | 47.68 | 16.6 | region4 | Sep-2005 | dor44 | *M. dorsalis* sp.1 |
| Mdor1588 | f | *Andricus inflator* | inflator | quercus | spring | shoot |  | Hungary | 18. Isaszeg | 47.53 | 19.4 | region1 | Jun-2006 | dor83 | *M. dorsalis* sp.2 |
| Mdor1589 | f | *Andricus inflator* | inflator | quercus | spring | shoot |  | Hungary | 18. Isaszeg | 47.53 | 19.4 | region1 | Jun-2006 | dor65 | *M. dorsalis* sp.2 |
| Mdor1590 | f | *Andricus inflator* | inflator | quercus | spring | shoot |  | Hungary | 18. Isaszeg | 47.53 | 19.4 | region1 | Jun-2006 | dor98 | *M. dorsalis* sp.2 |
| Mdor1591 | f | *Andricus multiplicatus* | multiplicatus | cerris | spring | leaf |  | Hungary | 11. Várpalota | 47.2 | 18.21 | region3 | Jun-2006 | dor94 | *M. dorsalis* sp.1 |
| Mdor1593 | m | *Andricus multiplicatus* | multiplicatus | cerris | spring | leaf |  | Hungary | 11. Várpalota | 47.2 | 18.21 | region3 | Jun-2006 | dor95 | *M. dorsalis* sp.1 |
| Mdor1594 | f | *Andricus multiplicatus* | multiplicatus | cerris | spring | leaf |  | Hungary | 11. Várpalota | 47.2 | 18.21 | region3 | Jun-2006 | dor97 | *M. dorsalis* sp.1 |
| Mdor1595 | f | *Biorhiza pallida* | Biorhiza | quercus | spring | bud |  | Austria | 5. Ober Pullendorf | 47.52 | 16.51 | region4 | Jun-2006 | dor84 | *M. dorsalis* sp.2 |
| Mdor1596 | m | *Biorhiza pallida* | Biorhiza | quercus | spring | bud |  | Austria | 5. Ober Pullendorf | 47.52 | 16.51 | region4 | Jun-2006 | dor65 | *M. dorsalis* sp.2 |
| Mdor1597 | f | *Biorhiza pallida* | Biorhiza | quercus | spring | bud |  | Austria | 5. Ober Pullendorf | 47.52 | 16.51 | region4 | Jun-2006 | dor65 | *M. dorsalis* sp.2 |
| Mdor1598 | f | *Biorhiza pallida* | Biorhiza | quercus | spring | bud |  | Austria | 5. Ober Pullendorf | 47.52 | 16.51 | region4 | Jun-2006 | dor106 | *M. dorsalis* sp.2 |
| Mdor1599 | f | *Biorhiza pallida* | Biorhiza | quercus | spring | bud |  | Austria | 5. Ober Pullendorf | 47.52 | 16.51 | region4 | Jun-2006 | dor110 | *M. dorsalis* sp.2 |
| Mdor1600 | m | *Biorhiza pallida* | Biorhiza | quercus | spring | bud |  | Austria | 5. Ober Pullendorf | 47.52 | 16.51 | region4 | Jun-2006 | dor109 | *M. dorsalis* sp.2 |
| Mdor1601 | f | *Biorhiza pallida* | Biorhiza | quercus | spring | bud |  | Austria | 5. Ober Pullendorf | 47.52 | 16.51 | region4 | Jun-2006 | dor106 | *M. dorsalis* sp.2 |
| Mdor1602 | m | *Biorhiza pallida* | Biorhiza | quercus | spring | bud |  | Hungary | 22. Mátrafüred | 47.83 | 19.97 | region2 | Jun-2006 | dor113 | *M. dorsalis* sp.2 |
| Mdor1604 | f | *Biorhiza pallida* | Biorhiza | quercus | spring | bud |  | Hungary | 22. Mátrafüred | 47.83 | 19.97 | region2 | Jun-2006 | dor112 | *M. dorsalis* sp.2 |
| Mdor1605 | f | *Biorhiza pallida* | Biorhiza | quercus | spring | bud |  | Hungary | 22. Mátrafüred | 47.83 | 19.97 | region2 | Jun-2006 | dor113 | *M. dorsalis* sp.2 |
| Mdor1606 | m | *Andricus multiplicatus* | multiplicatus | cerris | spring | leaf |  | Hungary | 4. Harka | 47.65 | 16.6 | region4 | Jun-2006 | dor2 | *M. dorsalis* sp.1 |
| Mdor1607 | m | *Andricus multiplicatus* | multiplicatus | cerris | spring | leaf |  | Hungary | 4. Harka | 47.65 | 16.6 | region4 | Jun-2006 | dor2 | *M. dorsalis* sp.1 |
| Mdor1608 | f | *Andricus multiplicatus* | multiplicatus | cerris | spring | leaf |  | Hungary | 4. Harka | 47.65 | 16.6 | region4 | Jun-2006 | dor21 | *M. dorsalis* sp.1 |
| Mdor1609 | f | *Andricus grossulariae* | lucidus | cerris | spring | catkin |  | Hungary | 4. Harka | 47.65 | 16.6 | region4 | Jun-2006 | dor120 | *M. dorsalis* sp.2 |
| Mdor1610 | m | *Andricus inflator* | inflator | quercus | spring | shoot |  | Austria | 6. Unterlois | 47.45 | 16.54 | region4 | Jun-2006 | dor92 | *M. dorsalis* sp.2 |
| Mdor1611 | f | *Pseudoneuroterus macropterus* | Pseudoneuroterus | cerris | autumn | shoot |  | Hungary | 21. Gyöngyössolymos | 47.82 | 19.93 | region2 | Apr-2006 | dor116 | *M. dorsalis* sp.2 |
| Mdor1612 | f | *Pseudoneuroterus macropterus* | Pseudoneuroterus | cerris | autumn | shoot |  | Hungary | 22. Mátrafüred | 47.83 | 19.97 | region2 | Jan-2006 | dor120 | *M. dorsalis* sp.2 |
| Mdor1619 | m | *Andricus kollari* | kollari | quercus | autumn | bud |  | Hungary | 18. Isaszeg | 47.53 | 19.4 | region1 | Sep-2005 | dor2 | *M. dorsalis* sp.1 |
| Mdor1637 | m | *Andricus grossulariae* | lucidus | cerris | spring | catkin |  | Hungary | 17. Gödöllö | 47.6 | 19.33 | region1 | Jun-2006 | dor129 | *M. dorsalis* sp.2 |
| Mdor1645 | m | *Andricus grossulariae* | lucidus | cerris | spring | catkin |  | Hungary | 17. Gödöllö | 47.6 | 19.33 | region1 | Jun-2006 | dor128 | *M. dorsalis* sp.2 |
| Mdor1655 | f | *Andricus grossulariae* | lucidus | cerris | spring | catkin |  | Hungary | 17. Gödöllö | 47.6 | 19.33 | region1 | Jun-2006 | dor130 | *M. dorsalis* sp.2 |
| Mdor1657 | f | *Andricus multiplicatus* | multiplicatus | cerris | spring | leaf |  | Hungary | 11. Várpalota | 47.2 | 18.21 | region3 | Jun-2006 | dor94 | *M. dorsalis* sp.1 |
| Mdor1660 | f | *Andricus multiplicatus* | multiplicatus | cerris | spring | leaf |  | Hungary | 11. Várpalota | 47.2 | 18.21 | region3 | Jun-2006 | dor134 | *M. dorsalis* sp.2 |
| Mdor1664 | f | *Andricus multiplicatus* | multiplicatus | cerris | spring | leaf |  | Hungary | 11. Várpalota | 47.2 | 18.21 | region3 | Jun-2006 | dor134 | *M. dorsalis* sp.2 |
| Mdor1667 | f | *Andricus grossulariae* | lucidus | cerris | spring | catkin |  | Hungary | 17. Gödöllö | 47.6 | 19.33 | region1 | Jun-2006 | dor131 | *M. dorsalis* sp.2 |
| Mdor1675 | f | *Andricus grossulariae* | lucidus | cerris | spring | catkin |  | Austria | 6. Unterlois | 47.45 | 16.54 | region4 | Jun-2006 | dor128 | *M. dorsalis* sp.2 |
| Mdor1678 | f | *Andricus multiplicatus* | multiplicatus | cerris | spring | leaf |  | Hungary | 11. Várpalota | 47.2 | 18.21 | region3 | Jun-2006 | dor134 | *M. dorsalis* sp.2 |
| Mdor1680 | f | *Andricus multiplicatus* | multiplicatus | cerris | spring | leaf |  | Hungary | 11. Várpalota | 47.2 | 18.21 | region3 | Jun-2006 | dor96 | *M. dorsalis* sp.1 |
| Mdor1706 | m | *Andricus infectorius* | kollari | quercus | autumn | bud |  | Hungary | 12. Orfü | 46.14 | 18.16 |  | Apr-2006 | dor135 | *M. dorsalis* sp.2 |
| Mdor1707 | f | *Biorhiza pallida* | Biorhiza | quercus | spring | bud |  | Hungary | 23. Sástó | 47.84 | 19.96 | region2 | Jul-2006 | dor84 | *M. dorsalis* sp.2 |
| Mdor1708 | m | *Biorhiza pallida* | Biorhiza | quercus | spring | bud |  | Hungary | 23. Sástó | 47.84 | 19.96 | region2 | Jul-2006 | dor2 | *M. dorsalis* sp.1 |
| Mdor1711 | f | *Biorhiza pallida* | Biorhiza | quercus | spring | bud |  | Hungary | 23. Sástó | 47.84 | 19.96 | region2 | Jun-2006 | dor110 | *M. dorsalis* sp.2 |
| Mdor1712 | f | *Andricus multiplicatus* | multiplicatus | cerris | spring | leaf |  | Hungary | 23. Sástó | 47.84 | 19.96 | region2 | Jun-2006 | dor21 | *M. dorsalis* sp.1 |
| Mdor1713 | f | *Andricus multiplicatus* | multiplicatus | cerris | spring | leaf |  | Hungary | 23. Sástó | 47.84 | 19.96 | region2 | Jun-2006 | dor21 | *M. dorsalis* sp.1 |
| Mdor1714 | f | *Andricus infectorius* | kollari | quercus | autumn | bud |  | Hungary | 12. Orfü | 46.14 | 18.16 |  | Apr-2006 | dor65 | *M. dorsalis* sp.2 |
| Mdor1715 | m | *Andricus multiplicatus* | multiplicatus | cerris | spring | leaf |  | Hungary | 23. Sástó | 47.84 | 19.96 | region2 | Jun-2006 | dor21 | *M. dorsalis* sp.1 |
| Mdor1716 | f | *Andricus glutinosus* | quercuscalicis | quercus | autumn | bud |  | Hungary | 23. Sástó | 47.84 | 19.96 | region2 | Feb-2006 | dor2 | *M. dorsalis* sp.1 |
| Mdor1717 | f | *Andricus glutinosus* | quercuscalicis | quercus | autumn | bud |  | Hungary | 23. Sástó | 47.84 | 19.96 | region2 | Feb-2006 | dor2 | *M. dorsalis* sp.1 |
| Mdor1718 | m | *Andricus glutinosus* | quercuscalicis | quercus | autumn | bud |  | Hungary | 23. Sástó | 47.84 | 19.96 | region2 | Feb-2006 | dor2 | *M. dorsalis* sp.1 |
| Mdor1721 | f | *Biorhiza pallida* | Biorhiza | quercus | spring | bud |  | Hungary | 23. Sástó | 47.84 | 19.96 | region2 | Jul-2005 | dor2 | *M. dorsalis* sp.1 |
| Mdor1724 | m | *Andricus multiplicatus* | multiplicatus | cerris | spring | leaf |  | Hungary | 15. Mariahalom | 47.63 | 18.71 |  | May-2002 | dor48 | *M. dorsalis* sp.1 |
| Mdor1725 | f | *Pseudoneuroterus macropterus* | Pseudoneuroterus | cerris | autumn | shoot |  | Hungary | 15. Mariahalom | 47.63 | 18.71 |  | Apr-2002 | dor2 | *M. dorsalis* sp.1 |
| Mdor1726 | m | *Andricus multiplicatus* | multiplicatus | cerris | spring | leaf |  | Hungary | 15. Mariahalom | 47.63 | 18.71 |  | Apr-2002 | dor4 | *M. dorsalis* sp.1 |
| Mdor1728 | m | *Pseudoneuroterus macropterus* | Pseudoneuroterus | cerris | autumn | shoot |  | Hungary | 7. Köszeg | 47.39 | 16.57 | region4 | Apr-2002 | dor90 | *M. dorsalis* sp.1 |
| Mdor1735 | f | *Pseudoneuroterus macropterus* | Pseudoneuroterus | cerris | autumn | shoot |  | Hungary | 2. Szarhalmi-forst | 47.7 | 16.64 | region4 | Nov-2005 | dor91 | *M. dorsalis* sp.2 |
| Mdor1736 | m | *Pseudoneuroterus macropterus* | Pseudoneuroterus | cerris | autumn | shoot |  | Hungary | 15. Mariahalom | 47.63 | 18.71 |  | Apr-2002 | dor2 | *M. dorsalis* sp.1 |
| Mdor1737 | m | *Aphelonyx cerricola* | Aphelonyx | cerris | autumn | bud |  | Hungary | 15. Mariahalom | 47.63 | 18.71 |  | Apr-2001 | dor2 | *M. dorsalis* sp.1 |
| Mdor1738 | f | *Aphelonyx cerricola* | Aphelonyx | cerris | autumn | bud |  | Hungary | 15. Mariahalom | 47.63 | 18.71 |  | Apr-2002 | dor4 | *M. dorsalis* sp.1 |
| Mdor1739 | m | *Aphelonyx cerricola* | Aphelonyx | cerris | autumn | bud |  | Hungary | 15. Mariahalom | 47.63 | 18.71 |  | Apr-2002 | dor4 | *M. dorsalis* sp.1 |
| Mdor1740 | f | *Aphelonyx cerricola* | Aphelonyx | cerris | autumn | bud |  | Hungary | 15. Mariahalom | 47.63 | 18.71 |  | Apr-2002 | dor4 | *M. dorsalis* sp.1 |
| Mdor1741 | m | *Andricus multiplicatus* | multiplicatus | cerris | spring | leaf |  | Hungary | 15. Mariahalom | 47.63 | 18.71 |  | Apr-2002 | dor4 | *M. dorsalis* sp.1 |
| Mdor1748 | f | *Andricus infectorius* | kollari | quercus | autumn | bud |  | Hungary | 19. Jászbereny | 47.5 | 19.93 |  | Oct-2004 | dor121 | *M. dorsalis* sp.2 |
| Mdor1805 | m | *Andricus inflator* | inflator | quercus | spring | shoot |  | Hungary | 18. Isaszeg | 47.53 | 19.4 | region1 | Jun-2006 | dor121 | *M. dorsalis* sp.2 |
| Mdor1806 | f | *Biorhiza pallida* | Biorhiza | quercus | spring | bud |  | Austria | 5. Ober Pullendorf | 47.52 | 16.51 | region4 | Jun-2006 | dor109 | *M. dorsalis* sp.2 |
| Mdor1808 | m | *Biorhiza pallida* | Biorhiza | quercus | spring | bud |  | Austria | 5. Ober Pullendorf | 47.52 | 16.51 | region4 | Jun-2006 | dor110 | *M. dorsalis* sp.2 |
| Mdor1809 | f | *Andricus inflator* | inflator | quercus | spring | shoot |  | Hungary | 18. Isaszeg | 47.53 | 19.4 | region1 | Jun-2006 | dor84 | *M. dorsalis* sp.2 |
| Mdor1815 | f | *Andricus grossulariae* | lucidus | cerris | spring | catkin |  | Hungary | 4. Harka | 47.65 | 16.6 | region4 | Jun-2006 | dor120 | *M. dorsalis* sp.2 |
| Mdor1817 | m | *Biorhiza pallida* | Biorhiza | quercus | spring | bud |  | Hungary | 24. Mátrahaza | 47.87 | 19.98 | region2 | Jun-2006 | dor123 | *M. dorsalis* sp.2 |
| Mdor1820 | m | *Andricus multiplicatus* | multiplicatus | cerris | spring | leaf |  | Hungary | 4. Harka | 47.65 | 16.6 | region4 | Jun-2006 | dor2 | *M. dorsalis* sp.1 |
| Mdor1821 | f | *Andricus infectorius* | kollari | quercus | autumn | bud |  | Hungary | 22. Mátrafüred | 47.83 | 19.97 | region2 | Sep-2005 | dor121 | *M. dorsalis* sp.2 |
| Mdor1823 | f | *Andricus grossulariae* | lucidus | cerris | spring | catkin |  | Hungary | 4. Harka | 47.65 | 16.6 | region4 | Jun-2006 | dor120 | *M. dorsalis* sp.2 |
| Mdor1824 | m | *Andricus grossulariae* | lucidus | cerris | spring | catkin |  | Hungary | 4. Harka | 47.65 | 16.6 | region4 | Jun-2006 | dor120 | *M. dorsalis* sp.2 |
| Mdor1825 | f | *Andricus multiplicatus* | multiplicatus | cerris | spring | leaf |  | Hungary | 4. Harka | 47.65 | 16.6 | region4 | Jun-2006 | dor2 | *M. dorsalis* sp.1 |
| Mdor1826 | f | *Biorhiza pallida* | Biorhiza | quercus | spring | bud |  | Hungary | 22. Mátrafüred | 47.83 | 19.97 | region2 | Jun-2006 | dor113 | *M. dorsalis* sp.2 |
| Mdor1827 | m | *Andricus multiplicatus* | multiplicatus | cerris | spring | leaf |  | Hungary | 4. Harka | 47.65 | 16.6 | region4 | Jun-2006 | dor2 | *M. dorsalis* sp.1 |
| Mdor1829 | m | *Andricus multiplicatus* | multiplicatus | cerris | spring | leaf |  | Hungary | 4. Harka | 47.65 | 16.6 | region4 | Jun-2006 | dor2 | *M. dorsalis* sp.1 |
| Mdor1830 | f | *Andricus multiplicatus* | multiplicatus | cerris | spring | leaf |  | Hungary | 4. Harka | 47.65 | 16.6 | region4 | Jun-2006 | dor21 | *M. dorsalis* sp.1 |
| Mdor1832 | m | *Biorhiza pallida* | Biorhiza | quercus | spring | bud |  | Austria | 5. Ober Pullendorf | 47.52 | 16.51 | region4 | Jun-2006 | dor106 | *M. dorsalis* sp.2 |
| Mdor1837 | m | *Andricus glutinosus* | quercuscalicis | quercus | autumn | bud |  | Hungary | 17. Gödöllö | 47.6 | 19.33 | region1 | Nov-1999 | dor2 | *M. dorsalis* sp.1 |
| Mdor1839 | f | *Andricus hungaricus* | quercuscalicis | quercus | autumn | bud |  | Hungary | 9. Devecser | 47.06 | 17.38 |  | Nov-1999 | dor136 | *M. dorsalis* sp.2 |
| Mdor1840 | f | *Andricus hungaricus* | quercuscalicis | quercus | autumn | bud |  | Hungary | 17. Gödöllö | 47.6 | 19.33 | region1 | Nov-1999 | dor2 | *M. dorsalis* sp.1 |
| Mdor1862 | m | *Aphelonyx cerricola* | Aphelonyx | cerris | autumn | bud |  | Hungary | 3. Sopron | 47.68 | 16.6 | region4 | Sep-2005 | dor2 | *M. dorsalis* sp.1 |
| Mdor1863 | m | *Aphelonyx cerricola* | Aphelonyx | cerris | autumn | bud |  | Hungary | 18. Isaszeg | 47.53 | 19.4 | region1 | Sep-2005 | dor122 | *M. dorsalis* sp.1 |
| Mdor1864 | m | *Aphelonyx cerricola* | Aphelonyx | cerris | autumn | bud |  | Hungary | 15. Mariahalom | 47.63 | 18.71 |  | Apr-2002 | dor2 | *M. dorsalis* sp.1 |
| Mdor1865 | m | *Aphelonyx cerricola* | Aphelonyx | cerris | autumn | bud |  | Hungary | 15. Mariahalom | 47.63 | 18.71 |  | Apr-2002 | dor2 | *M. dorsalis* sp.1 |
| Mdor1866 | f | *Aphelonyx cerricola* | Aphelonyx | cerris | autumn | bud |  | Hungary | 18. Isaszeg | 47.53 | 19.4 | region1 | Sep-2005 | dor2 | *M. dorsalis* sp.1 |
| Mdor1867 | f | *Biorhiza pallida* | Biorhiza | quercus | spring | bud |  | Austria | 5. Ober Pullendorf | 47.52 | 16.51 | region4 | Jun-2006 | dor84 | *M. dorsalis* sp.2 |
| Mdor1868 | f | *Biorhiza pallida* | Biorhiza | quercus | spring | bud |  | Hungary | 2. Szarhalmi-forst | 47.7 | 16.64 | region4 | Jun-1999 | dor2 | *M. dorsalis* sp.1 |
| Mdor1869 | m | *Biorhiza pallida* | Biorhiza | quercus | spring | bud |  | Hungary | 2. Szarhalmi-forst | 47.7 | 16.64 | region4 | Jun-1999 | dor2 | *M. dorsalis* sp.1 |
| Mdor1870 | m | *Biorhiza pallida* | Biorhiza | quercus | spring | bud |  | Hungary | 22. Mátrafüred | 47.83 | 19.97 | region2 | Jun-2006 | dor113 | *M. dorsalis* sp.2 |
| Mdor1871 | f | *Callirhytis glandium* | Callirhytis | cerris | autumn | acorn |  | Hungary | 22. Mátrafüred | 47.83 | 19.97 | region2 | Oct-2001 | dor124 | *M. dorsalis* sp.1 |
| Mdor1872 | m | *Pseudoneuroterus macropterus* | Pseudoneuroterus | cerris | autumn | shoot |  | Hungary | 16. Kemence | 48.02 | 18.89 |  | Nov-1999 | dor108 | *M. dorsalis* sp.1 |
| Mdor1873 | m | *Pseudoneuroterus macropterus* | Pseudoneuroterus | cerris | autumn | shoot |  | Hungary | 13. Pécsvárad | 46.15 | 18.42 |  | Dec-2005 | dor87 | *M. dorsalis* sp.2 |
| Mdor1874 | m | *Pseudoneuroterus macropterus* | Pseudoneuroterus | cerris | autumn | shoot |  | Hungary | 1. Fertörákos | 47.72 | 16.65 | region4 | Feb-2006 | dor117 | *M. dorsalis* sp.2 |
| Mdor1875 | m | *Pseudoneuroterus macropterus* | Pseudoneuroterus | cerris | autumn | shoot |  | Hungary | 21. Gyöngyössolymos | 47.82 | 19.93 | region2 | Apr-2006 | dor126 | *M. dorsalis* sp.2 |
| Mdor1876 | f | *Pseudoneuroterus macropterus* | Pseudoneuroterus | cerris | autumn | shoot |  | Hungary | 22. Mátrafüred | 47.83 | 19.97 | region2 | Jan-2006 | dor118 | *M. dorsalis* sp.2 |
| Mdor1877 | m | *Andricus glutinosus* | quercuscalicis | quercus | autumn | bud |  | Hungary | 17. Gödöllö | 47.6 | 19.33 | region1 | Nov-1999 | dor21 | *M. dorsalis* sp.1 |
| Mdor1878 | f | *Andricus hungaricus* | quercuscalicis | quercus | autumn | bud |  | Hungary | 9. Devecser | 47.06 | 17.38 |  | Nov-1999 | dor106 | *M. dorsalis* sp.2 |
| Mdor1879 | f | *Andricus hungaricus* | quercuscalicis | quercus | autumn | bud |  | Hungary | 17. Gödöllö | 47.6 | 19.33 | region1 | Nov-1999 | dor2 | *M. dorsalis* sp.1 |
| Mdor1880 | m | *Andricus hungaricus* | quercuscalicis | quercus | autumn | bud |  | Hungary | 17. Gödöllö | 47.6 | 19.33 | region1 | Nov-1999 | dor2 | *M. dorsalis* sp.1 |
| Mdor1883 | f | *Andricus glutinosus* | quercuscalicis | quercus | autumn | bud |  | Hungary | 22. Mátrafüred | 47.83 | 19.97 | region2 | Oct-2004 | dor2 | *M. dorsalis* sp.1 |
| Mdor1889 | f | *Biorhiza pallida* | Biorhiza | quercus | spring | bud |  | Hungary | 20. Jászapáti | 47.51 | 20.14 |  | May-2001 | dor2 | *M. dorsalis* sp.1 |
| Mdor1890 | f | *Biorhiza pallida* | Biorhiza | quercus | spring | bud |  | Hungary | 20. Jászapáti | 47.51 | 20.14 |  | May-2001 | dor10 | *M. dorsalis* sp.1 |
| Mdor2005 | m | *Biorhiza pallida* | Biorhiza | quercus | spring | bud |  | Austria | 5. Ober Pullendorf | 47.52 | 16.51 | region4 | Jun-2006 | dor110 | *M. dorsalis* sp.2 |
| Mdor2010 | f | *Biorhiza pallida* | Biorhiza | quercus | spring | bud |  | Austria | 5. Ober Pullendorf | 47.52 | 16.51 | region4 | Jun-2006 | dor83 | *M. dorsalis* sp.2 |
| Mdor2011 | m | *Andricus kollari* | kollari | quercus | autumn | bud |  | Hungary | 3. Sopron | 47.68 | 16.6 | region4 | Jun-2006 | dor137 | *M. dorsalis* sp.1 |
| Mdor2032 | f | *Andricus multiplicatus* | multiplicatus | cerris | spring | leaf |  | Hungary | 11. Várpalota | 47.2 | 18.21 | region3 | Jun-2006 | dor97 | *M. dorsalis* sp.1 |
| Mdor2039 | m | *Aphelonyx cerricola* | Aphelonyx | cerris | autumn | bud |  | Hungary | 18. Isaszeg | 47.53 | 19.4 | region1 | Sep-2005 | dor2 | *M. dorsalis* sp.1 |
| Mdor2050 | f | *Aphelonyx cerricola* | Aphelonyx | cerris | autumn | bud |  | Hungary | 18. Isaszeg | 47.53 | 19.4 | region1 | Sep-2005 | dor2 | *M. dorsalis* sp.1 |
| Mdor2053 | m | *Aphelonyx cerricola* | Aphelonyx | cerris | autumn | bud |  | Hungary | 18. Isaszeg | 47.53 | 19.4 | region1 | Sep-2005 | dor122 | *M. dorsalis* sp.1 |
| Mdor2054 | m | *Andricus multiplicatus* | multiplicatus | cerris | spring | leaf |  | Hungary | 11. Várpalota | 47.2 | 18.21 | region3 | Jun-2006 | dor138 | *M. dorsalis* sp.1 |
| Mdor2163 | f | *Andricus quercustozae* | quercuscalicis | quercus | autumn | bud |  | Hungary | 11. Várpalota | 47.2 | 18.21 | region3 | Jun-2006 | dor2 | *M. dorsalis* sp.1 |
| Mdor2187 | f | *Andricus grossulariae* | lucidus | cerris | spring | catkin |  | Hungary | 4. Harka | 47.65 | 16.6 | region4 | Jun-2006 | dor120 | *M. dorsalis* sp.2 |
| Mdor2188 | f | *Andricus grossulariae* | lucidus | cerris | spring | catkin |  | Hungary | 4. Harka | 47.65 | 16.6 | region4 | Jun-2006 | dor120 | *M. dorsalis* sp.2 |
| Mdor2189 | f | *Pseudoneuroterus macropterus* | Pseudoneuroterus | cerris | autumn | shoot |  | Hungary | 4. Harka | 47.65 | 16.6 | region4 | Jun-2006 | dor120 | *M. dorsalis* sp.2 |
| Mdor2190 | f | *Andricus grossulariae* | lucidus | cerris | spring | catkin |  | Hungary | 4. Harka | 47.65 | 16.6 | region4 | Jun-2006 | dor120 | *M. dorsalis* sp.2 |
| Mdor2192 | f | *Andricus grossulariae* | lucidus | cerris | spring | catkin |  | Hungary | 4. Harka | 47.65 | 16.6 | region4 | Jun-2006 | dor120 | *M. dorsalis* sp.2 |
| Mdor2193 | m | *Andricus grossulariae* | lucidus | cerris | spring | catkin |  | Hungary | 4. Harka | 47.65 | 16.6 | region4 | Jun-2006 | dor120 | *M. dorsalis* sp.2 |
| Mdor2196 | m | *Andricus grossulariae* | lucidus | cerris | spring | catkin |  | Hungary | 4. Harka | 47.65 | 16.6 | region4 | Jun-2006 | dor120 | *M. dorsalis* sp.2 |
| Mdor2198 | f | *Andricus grossulariae* | lucidus | cerris | spring | catkin |  | Austria | 6. Unterlois | 47.45 | 16.54 | region4 | Jun-2006 | dor144 | *M. dorsalis* sp.2 |
| Mdor2201 | f | *Biorhiza pallida* | Biorhiza | quercus | spring | bud |  | Austria | 5. Ober Pullendorf | 47.52 | 16.51 | region4 | Jun-2006 | dor110 | *M. dorsalis* sp.2 |
| Mdor2202 | m | *Biorhiza pallida* | Biorhiza | quercus | spring | bud |  | Austria | 5. Ober Pullendorf | 47.52 | 16.51 | region4 | Jun-2006 | dor110 | *M. dorsalis* sp.2 |
| Mdor2203 | f | *Biorhiza pallida* | Biorhiza | quercus | spring | bud |  | Austria | 5. Ober Pullendorf | 47.52 | 16.51 | region4 | Jun-2006 | dor106 | *M. dorsalis* sp.2 |
| Mdor2204 | m | *Biorhiza pallida* | Biorhiza | quercus | spring | bud |  | Austria | 5. Ober Pullendorf | 47.52 | 16.51 | region4 | Jun-2006 | dor139 | *M. dorsalis* sp.2 |
| Mdor2298 | f | *Aphelonyx cerricola* | Aphelonyx | cerris | autumn | bud |  | Hungary | 3. Sopron | 47.68 | 16.6 | region4 | Sep-2005 | dor2 | *M. dorsalis* sp.1 |
| Mdor2304 | f | *Andricus multiplicatus* | multiplicatus | cerris | spring | leaf |  | Hungary | 11. Várpalota | 47.2 | 18.21 | region3 | Jun-2006 | dor97 | *M. dorsalis* sp.1 |
| Mdor2307 | f | *Andricus grossulariae* | lucidus | cerris | spring | catkin |  | Hungary | 17. Gödöllö | 47.6 | 19.33 | region1 | Jun-2006 | dor149 | *M. dorsalis* sp.2 |
| Mdor2312 | m | *Andricus multiplicatus* | multiplicatus | cerris | spring | leaf |  | Hungary | 11. Várpalota | 47.2 | 18.21 | region3 | Jun-2006 | dor138 | *M. dorsalis* sp.1 |
| Mdor2313 | f | *Andricus grossulariae* | lucidus | cerris | spring | catkin |  | Austria | 6. Unterlois | 47.45 | 16.54 | region4 | Jun-2006 | dor140 | *M. dorsalis* sp.2 |
| Mdor2317 | f | *Andricus multiplicatus* | multiplicatus | cerris | spring | leaf |  | Hungary | 11. Várpalota | 47.2 | 18.21 | region3 | Jun-2006 | dor141 | *M. dorsalis* sp.2 |
| Mdor2321 | f | *Andricus multiplicatus* | multiplicatus | cerris | spring | leaf |  | Hungary | 11. Várpalota | 47.2 | 18.21 | region3 | Jun-2006 | dor2 | *M. dorsalis* sp.1 |
| Mdor2324 | m | *Pseudoneuroterus macropterus* | Pseudoneuroterus | cerris | autumn | shoot |  | Austria | 6. Unterlois | 47.45 | 16.54 | region4 | Jun-2006 | dor2 | *M. dorsalis* sp.1 |
| Mdor2332 | f | *Andricus hungaricus* | quercuscalicis | quercus | autumn | bud |  | Hungary | 18. Isaszeg | 47.53 | 19.4 | region1 | Sep-2005 | dor2 | *M. dorsalis* sp.1 |
| Mdor2337 | m | *Biorhiza pallida* | Biorhiza | quercus | spring | bud |  | Austria | 5. Ober Pullendorf | 47.52 | 16.51 | region4 | Jun-2006 | dor121 | *M. dorsalis* sp.2 |
| Mdor2338 | m | *Biorhiza pallida* | Biorhiza | quercus | spring | bud |  | Austria | 5. Ober Pullendorf | 47.52 | 16.51 | region4 | Jun-2006 | dor142 | *M. dorsalis* sp.2 |
| Mdor2346 | m | *Biorhiza pallida* | Biorhiza | quercus | spring | bud |  | Austria | 5. Ober Pullendorf | 47.52 | 16.51 | region4 | Jun-2006 | dor114 | *M. dorsalis* sp.2 |
| Mdor2349 | m | *Andricus multiplicatus* | multiplicatus | cerris | spring | leaf |  | Hungary | 4. Harka | 47.65 | 16.6 | region4 | Jun-2006 | dor2 | *M. dorsalis* sp.1 |
| Mdor2353 | m | *Andricus multiplicatus* | multiplicatus | cerris | spring | leaf |  | Hungary | 11. Várpalota | 47.2 | 18.21 | region3 | Jun-2006 | dor134 | *M. dorsalis* sp.2 |
| Mdor2356 | f | *Andricus infectorius* | kollari | quercus | autumn | bud |  | Hungary | 22. Mátrafüred | 47.83 | 19.97 | region2 | Sep-2005 | dor143 | *M. dorsalis* sp.2 |
| Mdor2357 | f | *Andricus grossulariae* | lucidus | cerris | spring | catkin |  | Hungary | 3. Sopron | 47.68 | 16.6 | region4 | Jun-2006 | dor2 | *M. dorsalis* sp.1 |
| Mdor2364 | f | *Andricus multiplicatus* | multiplicatus | cerris | spring | leaf |  | Hungary | 11. Várpalota | 47.2 | 18.21 | region3 | Jun-2006 | dor96 | *M. dorsalis* sp.1 |
| Mdor2366 | m | *Andricus multiplicatus* | multiplicatus | cerris | spring | leaf |  | Hungary | 11. Várpalota | 47.2 | 18.21 | region3 | Jun-2006 | dor2 | *M. dorsalis* sp.1 |
| Mdor2368 | m | *Andricus grossulariae* | lucidus | cerris | spring | catkin |  | Hungary | 17. Gödöllö | 47.6 | 19.33 | region1 | Jun-2006 | dor106 | *M. dorsalis* sp.2 |
| Mdor2388 | f | *Andricus grossulariae* | lucidus | cerris | spring | catkin |  | Austria | 6. Unterlois | 47.45 | 16.54 | region4 | Jun-2006 | dor144 | *M. dorsalis* sp.2 |
| Mdor2390 | f | *Andricus grossulariae* | lucidus | cerris | spring | catkin |  | Hungary | 17. Gödöllö | 47.6 | 19.33 | region1 | Jun-2006 | dor130 | *M. dorsalis* sp.2 |
| Mdor2391 | f | *Andricus grossulariae* | lucidus | cerris | spring | catkin |  | Hungary | 17. Gödöllö | 47.6 | 19.33 | region1 | Jun-2006 | dor145 | *M. dorsalis* sp.2 |
| Mdor2392 | f | *Andricus grossulariae* | lucidus | cerris | spring | catkin |  | Hungary | 17. Gödöllö | 47.6 | 19.33 | region1 | Jun-2006 | dor120 | *M. dorsalis* sp.2 |
| Mdor2393 | f | *Biorhiza pallida* | Biorhiza | quercus | spring | bud |  | Austria | 5. Ober Pullendorf | 47.52 | 16.51 | region4 | Jun-2006 | dor84 | *M. dorsalis* sp.2 |
| Mdor2394 | f | *Biorhiza pallida* | Biorhiza | quercus | spring | bud |  | Austria | 5. Ober Pullendorf | 47.52 | 16.51 | region4 | Jun-2006 | dor65 | *M. dorsalis* sp.2 |
| Mdor2395 | f | *Biorhiza pallida* | Biorhiza | quercus | spring | bud |  | Hungary | 22. Mátrafüred | 47.83 | 19.97 | region2 | Jun-2006 | dor112 | *M. dorsalis* sp.2 |
| Mdor2396 | f | *Biorhiza pallida* | Biorhiza | quercus | spring | bud |  | Austria | 5. Ober Pullendorf | 47.52 | 16.51 | region4 | Jun-2006 | dor106 | *M. dorsalis* sp.2 |
| Mdor2397 | m | *Andricus hungaricus* | quercuscalicis | quercus | autumn | bud |  | Hungary | 18. Isaszeg | 47.53 | 19.4 | region1 | Sep-2005 | dor2 | *M. dorsalis* sp.1 |
| Mdor2400 | f | *Andricus quercustozae* | quercuscalicis | quercus | autumn | bud |  | Hungary | 11. Várpalota | 47.2 | 18.21 | region3 | Jun-2006 | dor2 | *M. dorsalis* sp.1 |
| Mdor2401 | f | *Andricus quercustozae* | quercuscalicis | quercus | autumn | bud |  | Hungary | 11. Várpalota | 47.2 | 18.21 | region3 | Jun-2006 | dor2 | *M. dorsalis* sp.1 |
| Mdor2402 | m | *Andricus multiplicatus* | multiplicatus | cerris | spring | leaf |  | Hungary | 15. Mariahalom | 47.63 | 18.71 |  | Apr-2002 | dor45 | *M. dorsalis* sp.1 |
| Mdor2516 | m | *Pseudoneuroterus macropterus* | Pseudoneuroterus | cerris | autumn | shoot |  | Hungary | 15. Mariahalom | 47.63 | 18.71 |  | Apr-2002 | dor2 | *M. dorsalis* sp.1 |
| Mdor2517 | m | *Pseudoneuroterus macropterus* | Pseudoneuroterus | cerris | autumn | shoot |  | Hungary | 15. Mariahalom | 47.63 | 18.71 |  | Apr-2002 | dor2 | *M. dorsalis* sp.1 |
| Mdor2518 | m | *Aphelonyx cerricola* | Aphelonyx | cerris | autumn | bud |  | Hungary | 15. Mariahalom | 47.63 | 18.71 |  | Apr-2002 | dor2 | *M. dorsalis* sp.1 |
| Mdor2519 | m | *Aphelonyx cerricola* | Aphelonyx | cerris | autumn | bud |  | Hungary | 15. Mariahalom | 47.63 | 18.71 |  | Apr-2002 | dor2 | *M. dorsalis* sp.1 |
| Mdor2520 | m | *Aphelonyx cerricola* | Aphelonyx | cerris | autumn | bud |  | Hungary | 15. Mariahalom | 47.63 | 18.71 |  | Apr-2002 | dor2 | *M. dorsalis* sp.1 |
| Mdor2521 | m | *Aphelonyx cerricola* | Aphelonyx | cerris | autumn | bud |  | Hungary | 15. Mariahalom | 47.63 | 18.71 |  | Apr-2002 | dor4 | *M. dorsalis* sp.1 |
| Mdor2522 | f | *Aphelonyx cerricola* | Aphelonyx | cerris | autumn | bud |  | Hungary | 15. Mariahalom | 47.63 | 18.71 |  | Apr-2002 | dor4 | *M. dorsalis* sp.1 |
| Mdor2523 | f | *Aphelonyx cerricola* | Aphelonyx | cerris | autumn | bud |  | Hungary | 15. Mariahalom | 47.63 | 18.71 |  | Apr-2002 | dor2 | *M. dorsalis* sp.1 |
| Mdor2524 | f | *Andricus lignicolus* | kollari | quercus | autumn | bud |  | Hungary | 11. Várpalota | 47.2 | 18.21 | region3 | Nov-1999 | dor46 | *M. dorsalis* sp.1 |
| Mdor2525 | f | *Andricus lignicolus* | kollari | quercus | autumn | bud |  | Hungary | 11. Várpalota | 47.2 | 18.21 | region3 | Nov-1999 | dor2 | *M. dorsalis* sp.1 |
| Mdor2526 | f | *Andricus lignicolus* | kollari | quercus | autumn | bud |  | Hungary | 11. Várpalota | 47.2 | 18.21 | region3 | Nov-1999 | dor48 | *M. dorsalis* sp.1 |
| Mdor2527 | m | *Andricus lignicolus* | kollari | quercus | autumn | bud |  | Hungary | 11. Várpalota | 47.2 | 18.21 | region3 | Nov-1999 | dor21 | *M. dorsalis* sp.1 |
| Mdor2529 | f | *Andricus quercuscalicis* | quercuscalicis | quercus | autumn | acorn |  | Hungary | 28. Borsod | 48.26 | 20.77 |  | Oct-2004 | dor146 | *M. dorsalis* sp.2 |
| Mdor2530 | m | *Andricus quercuscalicis* | quercuscalicis | quercus | autumn | acorn |  | Hungary | 28. Borsod | 48.26 | 20.77 |  | Oct-2004 | dor84 | *M. dorsalis* sp.2 |
| Mdor2531 | m | *Pseudoneuroterus macropterus* | Pseudoneuroterus | cerris | autumn | shoot |  | Hungary | 22. Mátrafüred | 47.83 | 19.97 | region2 | Jan-2006 | dor120 | *M. dorsalis* sp.2 |
| Mdor2532 | f | *Pseudoneuroterus macropterus* | Pseudoneuroterus | cerris | autumn | shoot |  | Hungary | 22. Mátrafüred | 47.83 | 19.97 | region2 | Jan-2006 | dor120 | *M. dorsalis* sp.2 |
| Mdor2533 | f | *Pseudoneuroterus macropterus* | Pseudoneuroterus | cerris | autumn | shoot |  | Hungary | 21. Gyöngyössolymos | 47.82 | 19.93 | region2 | Apr-2006 | dor147 | *M. dorsalis* sp.2 |
| Mdor2534 | m | *Pseudoneuroterus macropterus* | Pseudoneuroterus | cerris | autumn | shoot |  | Hungary | 3. Sopron | 47.68 | 16.6 | region4 | Nov-1999 | dor2 | *M. dorsalis* sp.1 |
| Mdor2535 | m | *Pseudoneuroterus macropterus* | Pseudoneuroterus | cerris | autumn | shoot |  | Hungary | 3. Sopron | 47.68 | 16.6 | region4 | Nov-1999 | dor148 | *M. dorsalis* sp.1 |
| Mdor2536 | m | *Pseudoneuroterus macropterus* | Pseudoneuroterus | cerris | autumn | shoot |  | Hungary | 3. Sopron | 47.68 | 16.6 | region4 | Nov-1999 | dor2 | *M. dorsalis* sp.1 |
| Mdor2537 | m | *Pseudoneuroterus macropterus* | Pseudoneuroterus | cerris | autumn | shoot |  | Hungary | 3. Sopron | 47.68 | 16.6 | region4 | Nov-1999 | dor2 | *M. dorsalis* sp.1 |
| Mdor2538 | m | *Pseudoneuroterus macropterus* | Pseudoneuroterus | cerris | autumn | shoot |  | Hungary | 3. Sopron | 47.68 | 16.6 | region4 | Nov-1999 | dor2 | *M. dorsalis* sp.1 |
| Mdor2539 | f | *Pseudoneuroterus macropterus* | Pseudoneuroterus | cerris | autumn | shoot |  | Hungary | 3. Sopron | 47.68 | 16.6 | region4 | Nov-1999 | dor2 | *M. dorsalis* sp.1 |
| Mdor2540 | f | *Pseudoneuroterus macropterus* | Pseudoneuroterus | cerris | autumn | shoot |  | Hungary | 3. Sopron | 47.68 | 16.6 | region4 | Nov-1999 | dor2 | *M. dorsalis* sp.1 |
| Mdor2541 | f | *Pseudoneuroterus macropterus* | Pseudoneuroterus | cerris | autumn | shoot |  | Hungary | 3. Sopron | 47.68 | 16.6 | region4 | Nov-1999 | dor2 | *M. dorsalis* sp.1 |
| Mdor2542 | f | *Pseudoneuroterus macropterus* | Pseudoneuroterus | cerris | autumn | shoot |  | Hungary | 3. Sopron | 47.68 | 16.6 | region4 | Nov-1999 | dor2 | *M. dorsalis* sp.1 |
| Mdor2543 | f | *Aphelonyx cerricola* | Aphelonyx | cerris | autumn | bud |  | Hungary | 17. Gödöllö | 47.6 | 19.33 | region1 | Aug-2001 | dor2 | *M. dorsalis* sp.1 |
| Mdor2544 | m | *Aphelonyx cerricola* | Aphelonyx | cerris | autumn | bud |  | Hungary | 17. Gödöllö | 47.6 | 19.33 | region1 | Aug-2001 | dor2 | *M. dorsalis* sp.1 |
| Mdor2545 | m | *Andricus coriarius* | kollari | quercus | autumn | bud |  | Hungary | 11. Várpalota | 47.2 | 18.21 | region3 | Nov-1999 | dor2 | *M. dorsalis* sp.1 |
| Mdor2546 | m | *Pseudoneuroterus macropterus* | Pseudoneuroterus | cerris | autumn | shoot |  | Hungary | 3. Sopron | 47.68 | 16.6 | region4 | Nov-1999 | dor2 | *M. dorsalis* sp.1 |
| Mdor2547 | m | *Pseudoneuroterus macropterus* | Pseudoneuroterus | cerris | autumn | shoot |  | Hungary | 3. Sopron | 47.68 | 16.6 | region4 | Nov-1999 | dor2 | *M. dorsalis* sp.1 |
| Mdor2604 | f | *Andricus quercuscalicis* | quercuscalicis | quercus | autumn | acorn |  | Hungary | 28. Borsod | 48.26 | 20.77 |  | Oct-2004 | dor115 | *M. dorsalis* sp.2 |
| Mdor2605 | f | *Andricus quercuscalicis* | quercuscalicis | quercus | autumn | acorn |  | Hungary | 28. Borsod | 48.26 | 20.77 |  | Oct-2004 | dor106 | *M. dorsalis* sp.2 |
| Mdor2730 | f | *Synophrus politus* | Synophrus | cerris | autumn | shoot |  | Hungary | 29. Jósvafö | 48.48 | 20.55 |  | Apr-2006 | dor44 | *M. dorsalis* sp.1 |
| d2 |  | *Andricus kollari* | kollari | quercus | autumn |  | invader | UK | Teignmouth | 50.55 | -3.47 |  | Oct-2005 | dor13 | *M. dorsalis* sp.1 |
| d19 |  | *Andricus kollari* | kollari | quercus | autumn |  | invader | UK | Teignmouth | 50.55 | -3.47 |  | Oct-2005 | dor2 | *M. dorsalis* sp.1 |
| d30 |  | *Andricus kollari* | kollari | quercus | autumn |  | invader | UK | Teignmouth | 50.55 | -3.47 |  | Oct-2005 | dor2 | *M. dorsalis* sp.1 |
| d31 |  | *Andricus kollari* | kollari | quercus | autumn |  | invader | UK | Teignmouth | 50.55 | -3.47 |  | Oct-2005 | dor2 | *M. dorsalis* sp.1 |
| d32 |  | *Andricus kollari* | kollari | quercus | autumn |  | invader | UK | Teignmouth | 50.55 | -3.47 |  | Oct-2005 | dor2 | *M. dorsalis* sp.1 |
| d33 |  | *Andricus kollari* | kollari | quercus | autumn |  | invader | UK | Teignmouth | 50.55 | -3.47 |  | Oct-2005 | dor2 | *M. dorsalis* sp.1 |
| d36 |  | *Andricus kollari* | kollari | quercus | autumn |  | invader | UK | Teignmouth | 50.55 | -3.47 |  | Oct-2005 | dor2 | *M. dorsalis* sp.1 |
| d46 |  | *Andricus kollari* | kollari | quercus | autumn |  | invader | UK | Teignmouth | 50.55 | -3.47 |  | Oct-2005 | dor2 | *M. dorsalis* sp.1 |
| Mdor0585 |  | *Biorhiza pallida* | Biorhiza | quercus | spring |  | native | UK | Knighton Park | 52.63 | -1.13 |  | Jun-2004 | dor2 | *M. dorsalis* sp.1 |
| Mdor0586 |  | *Biorhiza pallida* | Biorhiza | quercus | spring |  | native | UK | Knighton Park | 52.63 | -1.13 |  | Jun-2004 | dor2 | *M. dorsalis* sp.1 |
| Mdor1331 |  | *Andricus grossulariae* | lucidus | cerris | spring |  | invader | UK | Silwood Park | 51.41 | -0.64 |  | Aug-2005 | dor32 | *M. dorsalis* sp.1 |
| Mdor1332 |  | *Andricus grossulariae* | lucidus | cerris | spring |  | invader | UK | Puttenham Common | 51.82 | -0.72 |  | Jul-2005 | dor2 | *M. dorsalis* sp.1 |
| Mdor1333 |  | *Andricus grossulariae* | lucidus | cerris | spring |  | invader | UK | Silwood Park | 51.41 | -0.64 |  | Jul-2005 | dor5 | *M. dorsalis* sp.1 |
| Mdor1334 |  | *Andricus grossulariae* | lucidus | cerris | spring |  | invader | UK | Puttenham Common | 51.82 | -0.72 |  | Jul-2005 | dor2 | *M. dorsalis* sp.1 |
| Mdor1335 |  | *Andricus grossulariae* | lucidus | cerris | spring |  | invader | UK | Silwood Park | 51.41 | -0.64 |  | Jul-2005 | dor2 | *M. dorsalis* sp.1 |
| Mdor1336 |  | *Andricus grossulariae* | lucidus | cerris | spring |  | invader | UK | Puttenham Common | 51.82 | -0.72 |  | Jul-2005 | dor2 | *M. dorsalis* sp.1 |
| Mdor1353 |  | *Andricus foecundatrix* | foecundatrix | quercus | autumn |  | native | UK | Windsor Great Park | 51.43 | -0.62 |  | Sep-2005 | dor37 | *M. dorsalis* sp.1 |
| Mdor1356 |  | *Andricus foecundatrix* | foecundatrix | quercus | autumn |  | native | UK | Faggs Wood | 51.07 | 0.84 |  | Sep-2005 | dor2 | *M. dorsalis* sp.1 |
| Mdor1358 |  | *Andricus foecundatrix* | foecundatrix | quercus | autumn |  | native | UK | Faggs Wood | 51.07 | 0.84 |  | Sep-2005 | dor38 | *M. dorsalis* sp.1 |
| Mdor1359 |  | *Andricus foecundatrix* | foecundatrix | quercus | autumn |  | native | UK | Faggs Wood | 51.07 | 0.84 |  | Sep-2005 | dor38 | *M. dorsalis* sp.1 |
| Mdor1456 |  | *Andricus quercuscalicis* | quercuscalicis | cerris | spring |  | invader | UK | Lancaster | 54.05 | -2.78 |  | Nov-2000 | dor5 | *M. dorsalis* sp.1 |
| Mdor1527 |  | *Biorhiza pallida* | Biorhiza | quercus | spring |  | native | UK | Silwood Park | 51.41 | -0.64 |  | Jun-2006 | dor2 | *M. dorsalis* sp.1 |
| Mdor1529 |  | *Biorhiza pallida* | Biorhiza | quercus | spring |  | native | UK | Silwood Park | 51.41 | -0.64 |  | Jun-2006 | dor2 | *M. dorsalis* sp.1 |
| Mdor1531 |  | *Biorhiza pallida* | Biorhiza | quercus | spring |  | native | UK | Silwood Park | 51.41 | -0.64 |  | Jun-2006 | dor2 | *M. dorsalis* sp.1 |
| Mdor1535 |  | *Biorhiza pallida* | Biorhiza | quercus | spring |  | native | UK | Silwood Park | 51.41 | -0.64 |  | Jun-2006 | dor2 | *M. dorsalis* sp.1 |
| Mdor1536 |  | *Biorhiza pallida* | Biorhiza | quercus | spring |  | native | UK | Silwood Park | 51.41 | -0.64 |  | Jun-2006 | dor2 | *M. dorsalis* sp.1 |
| Mdor1539 |  | *Biorhiza pallida* | Biorhiza | quercus | spring |  | native | UK | Silwood Park | 51.41 | -0.64 |  | Jun-2006 | dor2 | *M. dorsalis* sp.1 |
| Mdor1540 |  | *Biorhiza pallida* | Biorhiza | quercus | spring |  | native | UK | Silwood Park | 51.41 | -0.64 |  | Jun-2006 | dor5 | *M. dorsalis* sp.1 |
| Mdor1541 |  | *Biorhiza pallida* | Biorhiza | quercus | spring |  | native | UK | Silwood Park | 51.41 | -0.64 |  | Jun-2006 | dor2 | *M. dorsalis* sp.1 |
| Mdor1542 |  | *Biorhiza pallida* | Biorhiza | quercus | spring |  | native | UK | Silwood Park | 51.41 | -0.64 |  | Jun-2006 | dor2 | *M. dorsalis* sp.1 |
| Mdor1544 |  | *Biorhiza pallida* | Biorhiza | quercus | spring |  | native | UK | Puttenham Common | 51.82 | -0.72 |  | Jun-2006 | dor2 | *M. dorsalis* sp.1 |
| Mdor1545 |  | *Biorhiza pallida* | Biorhiza | quercus | spring |  | native | UK | Puttenham Common | 51.82 | -0.72 |  | Jun-2006 | dor2 | *M. dorsalis* sp.1 |
| Mdor1546 |  | *Biorhiza pallida* | Biorhiza | quercus | spring |  | native | UK | Puttenham Common | 51.82 | -0.72 |  | Jun-2006 | dor2 | *M. dorsalis* sp.1 |
| Mdor1548 |  | *Biorhiza pallida* | Biorhiza | quercus | spring |  | native | UK | Silwood Park | 51.41 | -0.64 |  | Jun-2006 | dor2 | *M. dorsalis* sp.1 |
| Mdor1816 |  | *Andricus quercuscalicis* | quercuscalicis | quercus | autumn |  | invader | UK | Bovey Tracey | 50.59 | -3.68 |  | Oct-2005 | dor2 | *M. dorsalis* sp.1 |
| Mdor2019 |  | *Andricus curvator* | foecundatrix | quercus | spring |  | native | UK | Dawlish Sands | 50.6 | -3.45 |  | Jun-2006 | dor2 | *M. dorsalis* sp.1 |
| Mdor2607 |  | *Aphelonyx cerricola* | Aphelonyx | cerris | autumn |  | invader | UK | Windsor Great Park | 51.43 | -0.62 |  | Sep-2006 | dor2 | *M. dorsalis* sp.1 |
| Mdor2739 |  | *Andricus kollari* | kollari | quercus | autumn |  | invader | UK | Windsor Great Park | 51.43 | -0.62 |  | Sep-2005 | dor2 | *M. dorsalis* sp.1 |
| Mdor2767 |  | *Andricus foecundatrix* | foecundatrix | quercus | autumn |  | native | UK | Farnham Park | 51.54 | -0.61 |  | Sep-2006 | dor2 | *M. dorsalis* sp.1 |
| Mdor2776 |  | *Andricus foecundatrix* | foecundatrix | quercus | autumn |  | native | UK | Farnham Park | 51.54 | -0.61 |  | Sep-2006 | dor2 | *M. dorsalis* sp.1 |
| Mdor2778 |  | *Andricus foecundatrix* | foecundatrix | quercus | autumn |  | native | UK | Farnham Park | 51.54 | -0.61 |  | Sep-2006 | dor2 | *M. dorsalis* sp.1 |
| Mdor2779 |  | *Andricus foecundatrix* | foecundatrix | quercus | autumn |  | native | UK | Farnham Park | 51.54 | -0.61 |  | Sep-2006 | dor2 | *M. dorsalis* sp.1 |
| Mdor2783 |  | *Andricus foecundatrix* | foecundatrix | quercus | autumn |  | native | UK | Farnham Park | 51.54 | -0.61 |  | Sep-2006 | dor2 | *M. dorsalis* sp.1 |
| Mdor2789 |  | *Aphelonyx cerricola* | Aphelonyx | cerris | autumn |  | invader | UK | Maidenhead Thicket | 51.52 | -0.72 |  | Sep-2006 | dor2 | *M. dorsalis* sp.1 |
| Mdor2793 |  | *Aphelonyx cerricola* | Aphelonyx | cerris | autumn |  | invader | UK | Maidenhead Thicket | 51.52 | -0.72 |  | Sep-2006 | dor5 | *M. dorsalis* sp.1 |
| Mdor2835 |  | *Andricus foecundatrix* | foecundatrix | quercus | autumn |  | native | UK | Farnham Park | 51.54 | -0.61 |  | Sep-2006 | dor2 | *M. dorsalis* sp.1 |
| MdorT5546 |  | *Andricus aries* | kollari | quercus | autumn |  | invader | UK | Ascot | 51.41 | -0.67 |  | Sep-2005 | dor2 | *M. dorsalis* sp.1 |
| MdorT5653 |  | *Andricus aries* | kollari | quercus | autumn |  | invader | UK | Ascot | 51.41 | -0.67 |  | Sep-2005 | dor2 | *M. dorsalis* sp.1 |
| MdorT5660 |  | *Andricus aries* | kollari | quercus | autumn |  | invader | UK | Ascot | 51.41 | -0.67 |  | Sep-2005 | dor2 | *M. dorsalis* sp.1 |
| MdorT5680 |  | *Andricus aries* | kollari | quercus | autumn |  | invader | UK | Ascot | 51.41 | -0.67 |  | Sep-2005 | dor2 | *M. dorsalis* sp.1 |
| MdorT5710 |  | *Andricus aries* | kollari | quercus | autumn |  | invader | UK | Ascot | 51.41 | -0.67 |  | Sep-2005 | dor2 | *M. dorsalis* sp.1 |
| MdorT5740 |  | *Andricus aries* | kollari | quercus | autumn |  | invader | UK | Ascot | 51.41 | -0.67 |  | Sep-2005 | dor2 | *M. dorsalis* sp.1 |
| Mdumicola |  |  |  | cerris |  |  |  | Spain |  |  |  |  |  | dum1 | *M. dumicola* |
| Mstigmatizans |  |  |  | quercus |  |  |  | Iran | Shena | 33.55 | 48.08 |  |  | sti2 | *M. stigmatizans* |
| Msynophri |  |  |  | cerris |  |  |  | Lebanon | Fraidah | 34.58 | 36.3 |  |  | syn1 | *M. synophri* |
|  |  |  |  |  |  |  |  |  |  |  |  |  |  |  |  |
| d1 |  | *Andricus kollari* |  | quercus |  |  |  | France |  |  |  |  |  | dor2 | *M. dorsalis* sp.1 |
| d3 |  | *Andricus coriarius* |  | quercus |  |  |  | Italy |  |  |  |  |  | dor14 | *M. dorsalis* sp.1 |
| d4 |  | *Andricus coriarius* |  | quercus |  |  |  | Turkey |  |  |  |  |  | dor2 | *M. dorsalis* sp.1 |
| d5 |  | *Andricus lucidus* |  | quercus |  |  |  | Iran |  |  |  |  |  | dor15 | *M. dorsalis* sp.1 |
| d6 |  | *Andricus lucidus* |  | quercus |  |  |  | Iran |  |  |  |  |  | dor16 | *M. dorsalis* sp.1 |
| d7 |  | *Andricus grossulariae* |  | quercus |  |  |  | Iran |  |  |  |  |  | dor17 | *M. dorsalis* sp.1 |
| d14 |  | *Andricus lignicolus* |  | quercus |  |  |  | Germany |  |  |  |  |  | dor2 | *M. dorsalis* sp.1 |
| d15 |  | *Andricus lucidus* |  | quercus |  |  |  | Turkey |  |  |  |  |  | dor9 | *M. dorsalis* sp.1 |
| d16 |  | *Andricus coriarius* |  | quercus |  |  |  | Greece |  |  |  |  |  | dor10 | *M. dorsalis* sp.1 |
| d17 |  | *Andricus kollari* |  | quercus |  |  |  | France |  |  |  |  |  | dor2 | *M. dorsalis* sp.1 |
| d18 |  | *Andricus kollari* |  | quercus |  |  |  | France |  |  |  |  |  | dor2 | *M. dorsalis* sp.1 |
| d20 |  | *Andricus grossulariae* |  | quercus |  |  |  | Turkey |  |  |  |  |  | dor11 | *M. dorsalis* sp.1 |
| d21 |  | *Andricus coriarius* |  | quercus |  |  |  | Turkey |  |  |  |  |  | dor3 | *M. dorsalis* sp.1 |
| d22 |  | *Andricus grossulariae* |  | quercus |  |  |  | Italy |  |  |  |  |  | dor2 | *M. dorsalis* sp.1 |
| d24.5 |  | *Andricus coriarius* |  | quercus |  |  |  | Italy |  |  |  |  |  | dor3 | *M. dorsalis* sp.1 |
| d25 |  | *Andricus coriarius* |  | quercus |  |  |  | Italy |  |  |  |  |  | dor3 | *M. dorsalis* sp.1 |
| d26 |  | *Andricus coriarius* |  | quercus |  |  |  | Italy |  |  |  |  |  | dor3 | *M. dorsalis* sp.1 |
| d28 |  | *Andricus coriarius* |  | quercus |  |  |  | Italy |  |  |  |  |  | dor12 | *M. dorsalis* sp.1 |
| d29 |  | *Andricus coriarius* |  | quercus |  |  |  | Italy |  |  |  |  |  | dor12 | *M. dorsalis* sp.1 |
| d34 |  | *Andricus kollari* |  | quercus |  |  |  | France |  |  |  |  |  | dor5 | *M. dorsalis* sp.1 |
| d35 |  | *Andricus kollari* |  | quercus |  |  |  | France |  |  |  |  |  | dor2 | *M. dorsalis* sp.1 |
| d38 |  | *Andricus coriarius* |  | quercus |  |  |  | Spain |  |  |  |  |  | dor2 | *M. dorsalis* sp.1 |
| d39 |  | *Andricus coriarius* |  | quercus |  |  |  | Spain |  |  |  |  |  | dor6 | *M. dorsalis* sp.1 |
| d41 |  | *Andricus grossulariae* |  | quercus |  |  |  | Turkey |  |  |  |  |  | dor7 | *M. dorsalis* sp.1 |
| d42 |  | *Andricus lucidus* |  | quercus |  |  |  | Italy |  |  |  |  |  | dor8 | *M. dorsalis* sp.1 |
| d43 |  | *Andricus coriarius* |  | quercus |  |  |  | Greece |  |  |  |  |  | dor2 | *M. dorsalis* sp.1 |
| d44 |  | *Andricus coriarius* |  | quercus |  |  |  | Greece |  |  |  |  |  | dor2 | *M. dorsalis* sp.1 |
| d47 |  | *Synophrus politus* |  | cerris |  |  |  | Greece |  |  |  |  |  | dor2 | *M. dorsalis* sp.1 |
| d48 |  | *Synophrus politus* |  | cerris |  |  |  | Greece |  |  |  |  |  | dor2 | *M. dorsalis* sp.1 |
| d49 |  | *Andricus coriarius* |  | quercus |  |  |  | Spain |  |  |  |  |  | dor6 | *M. dorsalis* sp.1 |
| Mdor0038 | f | *Andricus grossulariae* |  | cerris |  |  |  | Iran |  |  |  |  |  | dor28 | *M. dorsalis* sp.1 |
| Mdor0039 | f | *Andricus grossulariae* |  | cerris |  |  |  | Iran |  |  |  |  |  | dor40 | *M. dorsalis* sp.1 |
| Mdor0040 | f | *Andricus grossulariae* |  | cerris |  |  |  | Iran |  |  |  |  |  | dor41 | *M. dorsalis* sp.1 |
| Mdor0141 | f | *Andricus kollari* |  | quercus |  |  |  | France |  |  |  |  |  | dor2 | *M. dorsalis* sp.1 |
| Mdor0173 | f | *Andricus kollari* |  | quercus |  |  |  | UK |  |  |  |  |  | dor18 | *M. dorsalis* sp.2 |
| Mdor0174 | f | *Andricus grossulariae* |  | quercus |  |  |  | Turkey |  |  |  |  |  | dor19 | *M. dorsalis* sp.1 |
| Mdor0280 | f | *Andricus coriarius* |  | quercus |  |  |  | Greece |  |  |  |  |  | dor42 | *M. dorsalis* sp.1 |
| Mdor0300 | f | *Andricus coriarius* |  | quercus |  |  |  | Greece |  |  |  |  |  | dor2 | *M. dorsalis* sp.1 |
| Mdor0309 | f | *Andricus coriarius* |  | quercus |  |  |  | Greece |  |  |  |  |  | dor2 | *M. dorsalis* sp.1 |
| Mdor0311 | f | *Andricus kollari* |  | quercus |  |  |  | Spain |  |  |  |  |  | dor43 | *M. dorsalis* sp.2 |
| Mdor0312 | f | *Andricus kollari* |  | quercus |  |  |  | Spain |  |  |  |  |  | dor43 | *M. dorsalis* sp.2 |
| Mdor0322 | f | *Andricus coriarius* |  | quercus |  |  |  | Greece |  |  |  |  |  | dor20 | *M. dorsalis* sp.1 |
| Mdor0324 | f | *Andricus coriarius* |  | quercus |  |  |  | Greece |  |  |  |  |  | dor21 | *M. dorsalis* sp.1 |
| Mdor0331 | f | *Andricus coriarius* |  | quercus |  |  |  | Greece |  |  |  |  |  | dor2 | *M. dorsalis* sp.1 |
| Mdor0334 | f | *Andricus coriarius* |  | quercus |  |  |  | Greece |  |  |  |  |  | dor2 | *M. dorsalis* sp.1 |
| Mdor0339 | f | *Andricus kollari* |  | quercus |  |  |  | Portugal |  |  |  |  |  | dor2 | *M. dorsalis* sp.1 |
| Mdor0344 | f | *Andricus quercustozae* |  | quercus |  |  |  | Italy |  |  |  |  |  | dor44 | *M. dorsalis* sp.1 |
| Mdor0348 | f | *Andricus quercustozae* |  | quercus |  |  |  | Italy |  |  |  |  |  | dor2 | *M. dorsalis* sp.1 |
| Mdor0350 | f | *Andricus quercustozae* |  | quercus |  |  |  | Italy |  |  |  |  |  | dor2 | *M. dorsalis* sp.1 |
| Mdor0580 | f | *Andricus coriarius* |  | quercus |  |  |  | Turkey |  |  |  |  |  | dor16 | *M. dorsalis* sp.1 |
| Mdor0583 | m | *Biorhiza pallida* |  | quercus |  |  |  | France |  |  |  |  |  | dor2 | *M. dorsalis* sp.1 |
| Mdor0584 | f | *Biorhiza pallida* |  | quercus |  |  |  | France |  |  |  |  |  | dor2 | *M. dorsalis* sp.1 |
| Mdor0595 | m | *Andricus lucidus* |  | quercus |  |  |  | France |  |  |  |  |  | dor2 | *M. dorsalis* sp.1 |
| Mdor0609 | f | *Andricus kollari* |  | quercus |  |  |  | Italy |  |  |  |  |  | dor44 | *M. dorsalis* sp.1 |
| Mdor0611 | f | *Andricus kollari* |  | quercus |  |  |  | Portugal |  |  |  |  |  | dor2 | *M. dorsalis* sp.1 |
| Mdor0686 | f | *Andricus dentrimitratus* |  | quercus |  |  |  | Italy |  |  |  |  |  | dor44 | *M. dorsalis* sp.1 |
| Mdor0687 | m | *Andricus dentrimitratus* |  | quercus |  |  |  | Italy |  |  |  |  |  | dor50 | *M. dorsalis* sp.1 |
| Mdor0716 | f | *Andricus coriarius* |  | quercus |  |  |  | Italy |  |  |  |  |  | dor44 | *M. dorsalis* sp.1 |
| Mdor0720 | f | *Andricus quercustozae* |  | quercus |  |  |  | Italy |  |  |  |  |  | dor2 | *M. dorsalis* sp.1 |
| Mdor0724 | f | *Andricus grossulariae* |  | cerris |  |  |  | Iran |  |  |  |  |  | dor51 | *M. dorsalis* sp.1 |
| Mdor0725 | f | *Andricus grossulariae* |  | cerris |  |  |  | Iran |  |  |  |  |  | dor52 | *M. dorsalis* sp.2 |
| Mdor0752 | f | *Andricus grossulariae* |  | cerris |  |  |  | Iran |  |  |  |  |  | dor17 | *M. dorsalis* sp.1 |
| Mdor0753 | f | *Andricus grossulariae* |  | cerris |  |  |  | Iran |  |  |  |  |  | dor53 | *M. dorsalis* sp.2 |
| Mdor0803 | f | *Andricus chodjaii* |  | quercus |  |  |  | Iran |  |  |  |  |  | dor16 | *M. dorsalis* sp.1 |
| Mdor0804 | f | *Andricus chodjaii* |  | quercus |  |  |  | Iran |  |  |  |  |  | dor54 | *M. dorsalis* sp.1 |
| Mdor0805 | f | *Andricus chodjaii* |  | quercus |  |  |  | Iran |  |  |  |  |  | dor55 | *M. dorsalis* sp.1 |
| Mdor0806 | f | *Andricus chodjaii* |  | quercus |  |  |  | Iran |  |  |  |  |  | dor56 | *M. dorsalis* sp.1 |
| Mdor0826 | f | *Andricus lucidus* |  | quercus |  |  |  | Iran |  |  |  |  |  | dor57 | *M. dorsalis* sp.1 |
| Mdor0827 | f | *Andricus lucidus* |  | quercus |  |  |  | Iran |  |  |  |  |  | dor58 | *M. dorsalis* sp.1 |
| Mdor0828 | f | *Andricus lucidus* |  | quercus |  |  |  | Iran |  |  |  |  |  | dor58 | *M. dorsalis* sp.1 |
| Mdor0862 | f | *Andricus lucidus* |  | quercus |  |  |  | Iran |  |  |  |  |  | dor16 | *M. dorsalis* sp.1 |
| Mdor0863 | f | *Andricus lucidus* |  | quercus |  |  |  | Iran |  |  |  |  |  | dor59 | *M. dorsalis* sp.1 |
| Mdor0864 | f | *Andricus lucidus* |  | quercus |  |  |  | Iran |  |  |  |  |  | dor60 | *M. dorsalis* sp.1 |
| Mdor0896 | f | *Andricus grossulariae* |  | quercus |  |  |  | Iran |  |  |  |  |  | dor61 | *M. dorsalis* sp.1 |
| Mdor0897 | f | *Andricus grossulariae* |  | quercus |  |  |  | Iran |  |  |  |  |  | dor62 | *M. dorsalis* sp.1 |
| Mdor0906 | f | *Andricus quercustozae* |  | quercus |  |  |  | Iran |  |  |  |  |  | dor63 | *M. dorsalis* sp.2 |
| Mdor0907 | f | *Andricus quercustozae* |  | quercus |  |  |  | Iran |  |  |  |  |  | dor64 | *M. dorsalis* sp.1 |
| Mdor0934 | f | *Andricus coriarius* |  | quercus |  |  |  | Italy |  |  |  |  |  | dor2 | *M. dorsalis* sp.1 |
| Mdor0950 | f | *Andricus coriarius* |  | quercus |  |  |  | Italy |  |  |  |  |  | dor65 | *M. dorsalis* sp.2 |
| Mdor0960 | f | *Andricus coriarius* |  | quercus |  |  |  | Italy |  |  |  |  |  | dor66 | *M. dorsalis* sp.1 |
| Mdor1019 | m | *Andricus grossulariae* |  | quercus |  |  |  | Turkey |  |  |  |  |  | dor67 | *M. dorsalis* sp.1 |
| Mdor1021 | m | *Andricus grossulariae* |  | quercus |  |  |  | Turkey |  |  |  |  |  | dor68 | *M. dorsalis* sp.1 |
| Mdor1031 | m | *Andricus grossulariae* |  | quercus |  |  |  | Turkey |  |  |  |  |  | dor69 | *M. dorsalis* sp.1 |
| Mdor1034 | m | *Andricus grossulariae* |  | quercus |  |  |  | Turkey |  |  |  |  |  | dor70 | *M. dorsalis* sp.1 |
| Mdor1038 | m | *Andricus grossulariae* |  | quercus |  |  |  | Turkey |  |  |  |  |  | dor70 | *M. dorsalis* sp.1 |
| Mdor1045 | f | *Andricus coriarius* |  | quercus |  |  |  | Turkey |  |  |  |  |  | dor71 | *M. dorsalis* sp.1 |
| Mdor1046 | f | *Andricus coriarius* |  | quercus |  |  |  | Turkey |  |  |  |  |  | dor19 | *M. dorsalis* sp.1 |
| Mdor1047 | m | *Andricus coriarius* |  | quercus |  |  |  | Turkey |  |  |  |  |  | dor72 | *M. dorsalis* sp.1 |
| Mdor1051 | f | *Andricus lucidus* |  | quercus |  |  |  | Turkey |  |  |  |  |  | dor40 | *M. dorsalis* sp.1 |
| Mdor1052 | f | *Andricus lucidus* |  | quercus |  |  |  | Turkey |  |  |  |  |  | dor73 | *M. dorsalis* sp.1 |
| Mdor1053 | f | *Andricus lucidus* |  | quercus |  |  |  | Turkey |  |  |  |  |  | dor73 | *M. dorsalis* sp.1 |
| Mdor1067 | m | *Andricus lucidus* |  | quercus |  |  |  | Turkey |  |  |  |  |  | dor74 | *M. dorsalis* sp.1 |
| Mdor1073 | f | *Andricus coriarius* |  | quercus |  |  |  | Greece |  |  |  |  |  | dor39 | *M. dorsalis* sp.1 |
| Mdor1074 | f | *Andricus coriarius* |  | quercus |  |  |  | Greece |  |  |  |  |  | dor2 | *M. dorsalis* sp.1 |
| Mdor1075 | f | *Andricus coriarius* |  | quercus |  |  |  | Greece |  |  |  |  |  | dor75 | *M. dorsalis* sp.1 |
| Mdor1076 | f | *Andricus coriarius* |  | quercus |  |  |  | Greece |  |  |  |  |  | dor2 | *M. dorsalis* sp.1 |
| Mdor1077 | f | *Andricus coriarius* |  | quercus |  |  |  | Greece |  |  |  |  |  | dor2 | *M. dorsalis* sp.1 |
| Mdor1080 | m | *Andricus coronatus* |  | quercus |  |  |  | Greece |  |  |  |  |  | dor22 | *M. dorsalis* sp.1 |
| Mdor1134 | f | *Andricus lucidus* |  | quercus |  |  |  | Turkey |  |  |  |  |  | dor2 | *M. dorsalis* sp.1 |
| Mdor1135 | f | *Andricus lucidus* |  | quercus |  |  |  | Turkey |  |  |  |  |  | dor48 | *M. dorsalis* sp.1 |
| Mdor1136 | f | *Andricus tomentosus* |  | quercus |  |  |  | Greece |  |  |  |  |  | dor2 | *M. dorsalis* sp.1 |
| Mdor1138 | f | *Andricus lignicolus* |  | quercus |  |  |  | France |  |  |  |  |  | dor23 | *M. dorsalis* sp.1 |
| Mdor1139 | f | *Andricus lignicolus* |  | quercus |  |  |  | France |  |  |  |  |  | dor2 | *M. dorsalis* sp.1 |
| Mdor1140 | f | *Andricus lignicolus* |  | quercus |  |  |  | France |  |  |  |  |  | dor5 | *M. dorsalis* sp.1 |
| Mdor1141 | f | *Andricus lignicolus* |  | quercus |  |  |  | France |  |  |  |  |  | dor5 | *M. dorsalis* sp.1 |
| Mdor1147 | f | *Andricus lignicolus* |  | quercus |  |  |  | France |  |  |  |  |  | dor2 | *M. dorsalis* sp.1 |
| Mdor1148 | f | *Andricus lignicolus* |  | quercus |  |  |  | France |  |  |  |  |  | dor5 | *M. dorsalis* sp.1 |
| Mdor1149 | f | *Andricus lignicolus* |  | quercus |  |  |  | France |  |  |  |  |  | dor2 | *M. dorsalis* sp.1 |
| Mdor1150 | f | *Andricus lignicolus* |  | quercus |  |  |  | France |  |  |  |  |  | dor5 | *M. dorsalis* sp.1 |
| Mdor1173 | f | *Andricus lignicolus* |  | quercus |  |  |  | France |  |  |  |  |  | dor2 | *M. dorsalis* sp.1 |
| Mdor1174 | f | *Andricus lignicolus* |  | quercus |  |  |  | France |  |  |  |  |  | dor5 | *M. dorsalis* sp.1 |
| Mdor1185 | f | *Andricus kollari* |  | quercus |  |  |  | France |  |  |  |  |  | dor5 | *M. dorsalis* sp.1 |
| Mdor1186 | f | *Andricus kollari* |  | quercus |  |  |  | France |  |  |  |  |  | dor2 | *M. dorsalis* sp.1 |
| Mdor1205 | f | *Andricus coriarius* |  | quercus |  |  |  | Turkey |  |  |  |  |  | dor24 | *M. dorsalis* sp.1 |
| Mdor1207 | f | *Andricus lucidus* |  | quercus |  |  |  | Italy |  |  |  |  |  | dor21 | *M. dorsalis* sp.1 |
| Mdor1209 | f | *Andricus lucidus* |  | quercus |  |  |  | Italy |  |  |  |  |  | dor2 | *M. dorsalis* sp.1 |
| Mdor1215 | f | *Andricus lucidus* |  | quercus |  |  |  | Italy |  |  |  |  |  | dor76 | *M. dorsalis* sp.1 |
| Mdor1217 | f | *Andricus lucidus* |  | quercus |  |  |  | Italy |  |  |  |  |  | dor77 | *M. dorsalis* sp.1 |
| Mdor1220 | f | *Andricus lucidus* |  | quercus |  |  |  | Italy |  |  |  |  |  | dor78 | *M. dorsalis* sp.1 |
| Mdor1222 | f | *Andricus lucidus* |  | quercus |  |  |  | Italy |  |  |  |  |  | dor44 | *M. dorsalis* sp.1 |
| Mdor1224 | f | *Andricus lucidus* |  | quercus |  |  |  | Italy |  |  |  |  |  | dor79 | *M. dorsalis* sp.1 |
| Mdor1226 | f | *Andricus lucidus* |  | quercus |  |  |  | Turkey |  |  |  |  |  | dor80 | *M. dorsalis* sp.1 |
| Mdor1233 | f | *Andricus quercustozae* |  | quercus |  |  |  | Italy |  |  |  |  |  | dor65 | *M. dorsalis* sp.2 |
| Mdor1234 | f | *Andricus quercustozae* |  | quercus |  |  |  | Italy |  |  |  |  |  | dor65 | *M. dorsalis* sp.2 |
| Mdor1241 | f | *Andricus dentrimitratus* |  | quercus |  |  |  | Italy |  |  |  |  |  | dor44 | *M. dorsalis* sp.1 |
| Mdor1242 | f | *Andricus kollari* |  | quercus |  |  |  | Italy |  |  |  |  |  | dor44 | *M. dorsalis* sp.1 |
| Mdor1243 | f | *Andricus kollari* |  | quercus |  |  |  | Italy |  |  |  |  |  | dor44 | *M. dorsalis* sp.1 |
| Mdor1244 | f | *Andricus kollari* |  | quercus |  |  |  | Italy |  |  |  |  |  | dor44 | *M. dorsalis* sp.1 |
| Mdor1245 | f | *Andricus kollari* |  | quercus |  |  |  | Italy |  |  |  |  |  | dor44 | *M. dorsalis* sp.1 |
| Mdor1246 | f | *Andricus kollari* |  | quercus |  |  |  | Italy |  |  |  |  |  | dor81 | *M. dorsalis* sp.1 |
| Mdor1247 | f | *Andricus kollari* |  | quercus |  |  |  | Italy |  |  |  |  |  | dor81 | *M. dorsalis* sp.1 |
| Mdor1248 | m | *Andricus dentrimitratus* |  | quercus |  |  |  | Italy |  |  |  |  |  | dor44 | *M. dorsalis* sp.1 |
| Mdor1268 | f | *Andricus inflator* |  | quercus |  |  |  | Eire |  |  |  |  |  | dor25 | *M. dorsalis* sp.2 |
| Mdor1269 | f | *Andricus caliciformis* |  | quercus |  |  |  | Greece |  |  |  |  |  | dor26 | *M. dorsalis* sp.1 |
| Mdor1270 | f | *Andricus caliciformis* |  | quercus |  |  |  | Greece |  |  |  |  |  | dor26 | *M. dorsalis* sp.1 |
| Mdor1271 | m | *Andricus caliciformis* |  | quercus |  |  |  | Greece |  |  |  |  |  | dor26 | *M. dorsalis* sp.1 |
| Mdor1272 | m | *Andricus caliciformis* |  | quercus |  |  |  | Greece |  |  |  |  |  | dor2 | *M. dorsalis* sp.1 |
| Mdor1298 | m | *Andricus kollari* |  | quercus |  |  |  | France |  |  |  |  |  | dor2 | *M. dorsalis* sp.1 |
| Mdor1299 | f | *Andricus coriarius* |  | quercus |  |  |  | Spain |  |  |  |  |  | dor2 | *M. dorsalis* sp.1 |
| Mdor1300 | f | *Andricus coriarius* |  | quercus |  |  |  | Spain |  |  |  |  |  | dor6 | *M. dorsalis* sp.1 |
| Mdor1301 | f | *Andricus coriarius* |  | quercus |  |  |  | Spain |  |  |  |  |  | dor2 | *M. dorsalis* sp.1 |
| Mdor1302 | f | *Andricus coriarius* |  | quercus |  |  |  | Spain |  |  |  |  |  | dor2 | *M. dorsalis* sp.1 |
| Mdor1303 | f | *Andricus coriarius* |  | quercus |  |  |  | Spain |  |  |  |  |  | dor2 | *M. dorsalis* sp.1 |
| Mdor1304 | f | *Andricus coriarius* |  | quercus |  |  |  | Spain |  |  |  |  |  | dor2 | *M. dorsalis* sp.1 |
| Mdor1305 | f | *Andricus coriarius* |  | quercus |  |  |  | Spain |  |  |  |  |  | dor27 | *M. dorsalis* sp.1 |
| Mdor1306 | f | *Andricus coriarius* |  | quercus |  |  |  | Spain |  |  |  |  |  | dor2 | *M. dorsalis* sp.1 |
| Mdor1307 | f | *Andricus coriarius* |  | quercus |  |  |  | Spain |  |  |  |  |  | dor2 | *M. dorsalis* sp.1 |
| Mdor1308 | f | *Andricus coriarius* |  | quercus |  |  |  | Spain |  |  |  |  |  | dor6 | *M. dorsalis* sp.1 |
| Mdor1309 | f | *Andricus coriarius* |  | quercus |  |  |  | Spain |  |  |  |  |  | dor6 | *M. dorsalis* sp.1 |
| Mdor1310 | f | *Andricus coriarius* |  | quercus |  |  |  | Spain |  |  |  |  |  | dor6 | *M. dorsalis* sp.1 |
| Mdor1311 | f | *Andricus coriarius* |  | quercus |  |  |  | Spain |  |  |  |  |  | dor2 | *M. dorsalis* sp.1 |
| Mdor1312 | f | *Andricus coriarius* |  | quercus |  |  |  | Spain |  |  |  |  |  | dor6 | *M. dorsalis* sp.1 |
| Mdor1313 | f | *Andricus coriarius* |  | quercus |  |  |  | Spain |  |  |  |  |  | dor6 | *M. dorsalis* sp.1 |
| Mdor1314 | f | *Andricus coriarius* |  | quercus |  |  |  | Spain |  |  |  |  |  | dor6 | *M. dorsalis* sp.1 |
| Mdor1317 | f | *Aphelonyx* sp. |  | cerris |  |  |  | Lebanon |  |  |  |  |  | dor28 | *M. dorsalis* sp.1 |
| Mdor1318 | m | *Aphelonyx* sp. |  | cerris |  |  |  | Lebanon |  |  |  |  |  | dor28 | *M. dorsalis* sp.1 |
| Mdor1320 | m | *Aphelonyx* sp. |  | cerris |  |  |  | Lebanon |  |  |  |  |  | dor28 | *M. dorsalis* sp.1 |
| Mdor1321 | f | *Aphelonyx* sp. |  | cerris |  |  |  | Lebanon |  |  |  |  |  | dor28 | *M. dorsalis* sp.1 |
| Mdor1322 | f | *Aphelonyx* sp. |  | cerris |  |  |  | Lebanon |  |  |  |  |  | dor28 | *M. dorsalis* sp.1 |
| Mdor1325 | f | *Andricus cecconii* |  | cerris |  |  |  | Lebanon |  |  |  |  |  | dor29 | *M. dorsalis* sp.1 |
| Mdor1326 | m | *Andricus cecconii* |  | cerris |  |  |  | Lebanon |  |  |  |  |  | dor29 | *M. dorsalis* sp.1 |
| Mdor1327 | m | *Aphelonyx* sp. |  | cerris |  |  |  | Lebanon |  |  |  |  |  | dor30 | *M. dorsalis* sp.2 |
| Mdor1328 | f | *Aphelonyx* sp. |  | cerris |  |  |  | Lebanon |  |  |  |  |  | dor31 | *M. dorsalis* sp.2 |
| Mdor1329 | f | *Aphelonyx* sp. |  | cerris |  |  |  | Lebanon |  |  |  |  |  | dor31 | *M. dorsalis* sp.2 |
| Mdor1330 | f | *Neuroterus quercusbaccarum* |  | quercus |  |  |  | UK |  |  |  |  |  | dor18 | *M. dorsalis* sp.2 |
| Mdor1337 | m | *Aphelonyx* sp. |  | cerris |  |  |  | Lebanon |  |  |  |  |  | dor28 | *M. dorsalis* sp.1 |
| Mdor1338 | f | *Aphelonyx* sp. |  | cerris |  |  |  | Lebanon |  |  |  |  |  | dor33 | *M. dorsalis* sp.2 |
| Mdor1339 | f | *Aphelonyx* sp. |  | cerris |  |  |  | Lebanon |  |  |  |  |  | dor33 | *M. dorsalis* sp.2 |
| Mdor1340 | f | *Aphelonyx* sp. |  | cerris |  |  |  | Lebanon |  |  |  |  |  | dor34 | *M. dorsalis* sp.2 |
| Mdor1341 | f | *Aphelonyx* sp. |  | cerris |  |  |  | Lebanon |  |  |  |  |  | dor35 | *M. dorsalis* sp.2 |
| Mdor1342 | f | *Aphelonyx* sp. |  | cerris |  |  |  | Lebanon |  |  |  |  |  | dor35 | *M. dorsalis* sp.2 |
| Mdor1343 | m | *Aphelonyx* sp. |  | cerris |  |  |  | Lebanon |  |  |  |  |  | dor34 | *M. dorsalis* sp.2 |
| Mdor1344 | f | *Aphelonyx* sp. |  | cerris |  |  |  | Lebanon |  |  |  |  |  | dor35 | *M. dorsalis* sp.2 |
| Mdor1345 | m | *Aphelonyx* sp. |  | cerris |  |  |  | Lebanon |  |  |  |  |  | dor36 | *M. dorsalis* sp.1 |
| Mdor1348 | m | *Andricus kollari* |  | quercus |  |  |  | Spain |  |  |  |  |  | dor2 | *M. dorsalis* sp.1 |
| Mdor1360 | f | *Andricus cecconii* |  | cerris |  |  |  | Lebanon |  |  |  |  |  | dor29 | *M. dorsalis* sp.1 |
| Mdor1361 | f | *Andricus cecconii* |  | cerris |  |  |  | Lebanon |  |  |  |  |  | dor29 | *M. dorsalis* sp.1 |
| Mdor1362 | f | *Andricus cecconii* |  | cerris |  |  |  | Lebanon |  |  |  |  |  | dor29 | *M. dorsalis* sp.1 |
| Mdor1413 | f | *Synophrus politus* |  | cerris |  |  |  | Italy |  |  |  |  |  | dor44 | *M. dorsalis* sp.1 |
| Mdor1414 | f | *Synophrus politus* |  | cerris |  |  |  | Italy |  |  |  |  |  | dor44 | *M. dorsalis* sp.1 |
| Mdor1447 | f | *Aphelonyx* sp. |  | cerris |  |  |  | Lebanon |  |  |  |  |  | dor82 | *M. dorsalis* sp.2 |
| Mdor1532 | f | *Biorhiza pallida* |  | quercus |  |  |  | UK |  |  |  |  |  | dor84 | *M. dorsalis* sp.2 |
| Mdor1551 | f | *Aphelonyx persica* |  | cerris |  |  |  | Turkey |  |  |  |  |  | dor85 | *M. dorsalis* sp.2 |
| Mdor1552 | m | *Andricus coriarius* |  | quercus |  |  |  | Lebanon |  |  |  |  |  | dor86 | *M. dorsalis* sp.2 |
| Mdor1709 | f | *Biorhiza pallida* |  | quercus |  |  |  | Romania |  |  |  |  |  | dor39 | *M. dorsalis* sp.1 |
| Mdor2758 | m | unknown gall |  | quercus |  |  |  | Finland |  |  |  |  |  | dor106 | *M. dorsalis* sp.2 |
| Mdor2760 | f | *Andricus inflator* |  | quercus |  |  |  | Finland |  |  |  |  |  | dor106 | *M. dorsalis* sp.2 |
| Mdor2761 | m | *Andricus inflator* |  | quercus |  |  |  | Finland |  |  |  |  |  | dor150 | *M. dorsalis* sp.2 |
