# Supplementary material for: Partitioning of herbivore hosts across time and food plants promotes diversification in the Megastigmus dorsalis oak gall parasitoid complex
Source: Ecol Evol. 2017 Dec 25;8(2):1300–15. doi: 10.1002/ece3.3712 (PMC5773290; doi:10.1002/ece3.3712)
Supplement: Supplementary file 2 [file ECE3-8-1300-s002.doc]

Appendix 2. Allelic data for the 112 *Megastigmus dorsalis* cryptic species 2 individuals genotyped at eight microsatellite loci. Alleles are named using their length in base pairs. Locus names follow Garnier *et al*. 2008. Males are haploid, hence have only one allele per locus. Allelic richness and expected heterozygosity (H_e_) for each locus are provided in the two final rows. The final two columns give assignment probabilities for each individual to each of the two genetic clusters from the *K* = 2 Structure analysis, averaged across the five replicate runs.

| **sample** | **gender** | **Mdo9** | | **Mst14** | | **Mdo12** | | **Mst13** | | **Mst2** | | **Mdo7A** | | **Mdo6** | | **Mdo16** | | **K=2, group1** | **K=2, group2** |
| --- | --- | --- | --- | --- | --- | --- | --- | --- | --- | --- | --- | --- | --- | --- | --- | --- | --- | --- | --- |
| Mdor0374 | female | 123 | 123 | 288 | 288 | 099 | 099 | 150 | 150 | 160 | 186 | 159 | 161 | 230 | 246 | 139 | 146 | 0.000 | 1.000 |
| Mdor0413 | female | 121 | 125 | 288 | 288 | 088 | 099 | 150 | 152 | 186 | 186 | 159 | 163 | 224 | 228 | 137 | 143 | 0.000 | 1.000 |
| Mdor0420 | male | 123 | - | 288 | - | 101 | - | 151 | - | 186 | - | 159 | - | 230 | - | 139 | - | 0.004 | 0.996 |
| Mdor0427 | male | 115 | - | 288 | - | 097 | - | 150 | - | 186 | - | 157 | - | 244 | - | 150 | - | 0.972 | 0.028 |
| Mdor0450 | female | 109 | 109 | 294 | 294 | 083 | 083 | 150 | 150 | 186 | 186 | 187 | 187 | 228 | 228 | 146 | 146 | 1.000 | 0.000 |
| Mdor1346 | female | 123 | 123 | 288 | 288 | 083 | 097 | 150 | 150 | 186 | 186 | 159 | 163 | 228 | 234 | 137 | 157 | 0.000 | 1.000 |
| Mdor1347 | female | 123 | 123 | 288 | 288 | 083 | 097 | 150 | 150 | 186 | 186 | 159 | 163 | 228 | 230 | 146 | 157 | 0.000 | 1.000 |
| Mdor1352 | male | 119 | - | 288 | - | 088 | - | 150 | - | 186 | - | 157 | - | 226 | - | 146 | - | 0.975 | 0.025 |
| Mdor1407 | female | 115 | 117 | 288 | 294 | 085 | 085 | 150 | 152 | 186 | 186 | 157 | 157 | 226 | 244 | 143 | 146 | 1.000 | 0.000 |
| Mdor1409 | female | 109 | 115 | 288 | 294 | 083 | 099 | 150 | 150 | 186 | 186 | 157 | 177 | 244 | 244 | 146 | 146 | 1.000 | 0.000 |
| Mdor1418 | male | 115 | - | 288 | - | 101 | - | 150 | - | 186 | - | 187 | - | 228 | - | 146 | - | 0.962 | 0.038 |
| Mdor1428 | male | 123 | - | 288 | - | 085 | - | 150 | - | 186 | - | 163 | - | 232 | - | 147 | - | 0.000 | 1.000 |
| Mdor1471 | female | 115 | 115 | 288 | 288 | 093 | 097 | 150 | 152 | 186 | 186 | 173 | 179 | 236 | 244 | 146 | 146 | 1.000 | 0.000 |
| Mdor1472 | male | 123 | - | 288 | - | 083 | - | 150 | - | 161 | - | 161 | - | 228 | - | 144 | - | 0.000 | 1.000 |
| Mdor1487 | male | 117 | - | 288 | - | 083 | - | 151 | - | 186 | - | 181 | - | 244 | - | 143 | - | 1.000 | 0.000 |
| Mdor1489 | female | 107 | 113 | 288 | 288 | 083 | 085 | 150 | 150 | 159 | 186 | 159 | 159 | 244 | 244 | 143 | 146 | 0.919 | 0.081 |
| Mdor1499 | female | 115 | 117 | 288 | 288 | 088 | 099 | 150 | 150 | 186 | 186 | 159 | 175 | 236 | 244 | 143 | 146 | 1.000 | 0.000 |
| Mdor1523 | female | 127 | 127 | 288 | 288 | 083 | 085 | 150 | 152 | 186 | 186 | 151 | 151 | 230 | 230 | 143 | 147 | 0.001 | 0.999 |
| Mdor1525 | female | 113 | 115 | 288 | 288 | 083 | 099 | 150 | 152 | 186 | 186 | 157 | 175 | 244 | 244 | 146 | 146 | 1.000 | 0.000 |
| Mdor1526 | male | 127 | - | 294 | - | 083 | - | 150 | - | 186 | - | 173 | - | 226 | - | 146 | - | 1.000 | 0.000 |
| Mdor1556 | male | 121 | - | 288 | - | 083 | - | 150 | - | 159 | - | 159 | - | 224 | - | 146 | - | 0.020 | 0.980 |
| Mdor1557 | male | 123 | - | 288 | - | 083 | - | 150 | - | 186 | - | 163 | - | 230 | - | 146 | - | 0.000 | 1.000 |
| Mdor1559 | female | 123 | 123 | 288 | 288 | 083 | 101 | 150 | 150 | 186 | 186 | 161 | 161 | 228 | 228 | 146 | 146 | 0.000 | 1.000 |
| Mdor1569 | female | 123 | 123 | 288 | 288 | 090 | 090 | 150 | 150 | 165 | 186 | 165 | 165 | 224 | 224 | 137 | 137 | 0.000 | 1.000 |
| Mdor1588 | female | 115 | 115 | 294 | 294 | 097 | 101 | 150 | 150 | 186 | 186 | 159 | 165 | 232 | 246 | 143 | 146 | 0.996 | 0.004 |
| Mdor1589 | female | 115 | 117 | 294 | 294 | 083 | 087 | 150 | 152 | 186 | 186 | 173 | 173 | 244 | 244 | 146 | 146 | 1.000 | 0.000 |
| Mdor1590 | female | 115 | 117 | 288 | 288 | 099 | 101 | 151 | 151 | 186 | 186 | 159 | 159 | 244 | 244 | 146 | 149 | 1.000 | 0.000 |
| Mdor1595 | female | 115 | 117 | 288 | 288 | 099 | 099 | 150 | 152 | 186 | 186 | 157 | 175 | 244 | 244 | 146 | 146 | 1.000 | 0.000 |
| Mdor1596 | male | 115 | - | 288 | - | 083 | - | 152 | - | 186 | - | 173 | - | 244 | - | 146 | - | 0.999 | 0.001 |
| Mdor1597 | female | 115 | 117 | 288 | 294 | 083 | 099 | 150 | 150 | 184 | 184 | 175 | 189 | 234 | 244 | 146 | 146 | 1.000 | 0.000 |
| Mdor1598 | female | 117 | 117 | 288 | 294 | 083 | 085 | 150 | 150 | 166 | 184 | 157 | 173 | 226 | 244 | 146 | 146 | 1.000 | 0.000 |
| Mdor1599 | female | 115 | 127 | 294 | 294 | 083 | 083 | 152 | 152 | 186 | 186 | 181 | 189 | 226 | 244 | 146 | 146 | 1.000 | 0.000 |
| Mdor1600 | male | 117 | - | 288 | - | 101 | - | 150 | - | 186 | - | 175 | - | 246 | - | 146 | - | 1.000 | 0.000 |
| Mdor1601 | female | 117 | 117 | 294 | 294 | 083 | 099 | 150 | 150 | 186 | 186 | 167 | 187 | 246 | 246 | 143 | 146 | 1.000 | 0.000 |
| Mdor1602 | male | 109 | - | 294 | - | 083 | - | 150 | - | 186 | - | 175 | - | 244 | - | 146 | - | 1.000 | 0.000 |
| Mdor1604 | female | 115 | 131 | 294 | 294 | 083 | 083 | 150 | 150 | 186 | 186 | 177 | 177 | 228 | 246 | 146 | 146 | 1.000 | 0.000 |
| Mdor1605 | female | 109 | 121 | 294 | 294 | 083 | 099 | 150 | 152 | 186 | 186 | 175 | 175 | 226 | 228 | 146 | 149 | 1.000 | 0.000 |
| Mdor1609 | female | 115 | 123 | 288 | 288 | 083 | 085 | 150 | 150 | 186 | 186 | 161 | 163 | 228 | 236 | 137 | 146 | 0.000 | 1.000 |
| Mdor1610 | male | 129 | - | 288 | - | 083 | - | 150 | - | 186 | - | 163 | - | 226 | - | 146 | - | 0.023 | 0.977 |
| Mdor1611 | female | 123 | 123 | 288 | 288 | 088 | 101 | 150 | 150 | 186 | 186 | 163 | 163 | 226 | 230 | 140 | 150 | 0.000 | 1.000 |
| Mdor1612 | female | - | - | 288 | 288 | 083 | 088 | 150 | 150 | 186 | 186 | 159 | 159 | 228 | 228 | 143 | 146 | 0.043 | 0.957 |
| Mdor1637 | male | 123 | - | 288 | - | 083 | - | 152 | - | 186 | - | 165 | - | 228 | - | 150 | - | 0.002 | 0.998 |
| Mdor1645 | male | 125 | - | 288 | - | 083 | - | - | - | 186 | - | 159 | - | 230 | - | 147 | - | 0.000 | 1.000 |
| Mdor1655 | female | 123 | 133 | 288 | 288 | 083 | 099 | 150 | 152 | 186 | 186 | 161 | 161 | 228 | 234 | 137 | 143 | 0.000 | 1.000 |
| Mdor1660 | female | 123 | 123 | 288 | 288 | 083 | 085 | 152 | 152 | 186 | 186 | 159 | 159 | 226 | 230 | 146 | 146 | 0.000 | 1.000 |
| Mdor1664 | female | 121 | 123 | 288 | 288 | 083 | 085 | 152 | 152 | 186 | 186 | 159 | 163 | 226 | 230 | 146 | 146 | 0.000 | 1.000 |
| Mdor1667 | female | 115 | 125 | - | - | 083 | 099 | 150 | 150 | 186 | 186 | 161 | 163 | 224 | 234 | 137 | 160 | 0.000 | 1.000 |
| Mdor1675 | female | 123 | 128 | 288 | 288 | 083 | 099 | 150 | 152 | 186 | 186 | 159 | 161 | 228 | 230 | 137 | 137 | 0.000 | 1.000 |
| Mdor1678 | female | 121 | 121 | 288 | 288 | 083 | 085 | 150 | 152 | 159 | 186 | 159 | 185 | 230 | 230 | 137 | 146 | 0.000 | 1.000 |
| Mdor1706 | male | 117 | - | 294 | - | 097 | - | 150 | - | 186 | - | 173 | - | 244 | - | 146 | - | 1.000 | 0.000 |
| Mdor1707 | female | 109 | 117 | 288 | 294 | 083 | 083 | 150 | 152 | 186 | 186 | 173 | 177 | 228 | 236 | 146 | 146 | 1.000 | 0.000 |
| Mdor1711 | female | 109 | 109 | 294 | 294 | 099 | 101 | 150 | 150 | 186 | 186 | 175 | 175 | 236 | 236 | 146 | 146 | 1.000 | 0.000 |
| Mdor1714 | female | 115 | 117 | 288 | 294 | 083 | 083 | 150 | 150 | 186 | 186 | 159 | 173 | 226 | 244 | 146 | 146 | 1.000 | 0.000 |
| Mdor1735 | female | 123 | 123 | 288 | 288 | 083 | 083 | 152 | 152 | 186 | 186 | 159 | 167 | 226 | 232 | 143 | 150 | 0.000 | 1.000 |
| Mdor1748 | female | 117 | 117 | 288 | 294 | 099 | 101 | 150 | 150 | 186 | 186 | 159 | 175 | 226 | 228 | 146 | 146 | 1.000 | 0.000 |
| Mdor1805 | male | 117 | - | 294 | - | 099 | - | 152 | - | 186 | - | 187 | - | 246 | - | 146 | - | 1.000 | 0.000 |
| Mdor1806 | female | 109 | 117 | 288 | 294 | 083 | 101 | 150 | 152 | 186 | 186 | 175 | 177 | 232 | 246 | 146 | 146 | 1.000 | 0.000 |
| Mdor1808 | male | 117 | - | 294 | - | 099 | - | 150 | - | 186 | - | 177 | - | 244 | - | 146 | - | 1.000 | 0.000 |
| Mdor1809 | female | 115 | 117 | 288 | 294 | 083 | 099 | 150 | 150 | 186 | 186 | 159 | 159 | 228 | 244 | 146 | 146 | 1.000 | 0.000 |
| Mdor1815 | female | 115 | 123 | 288 | 288 | 085 | 097 | 150 | 150 | 186 | 186 | 161 | 163 | 228 | 228 | 137 | 146 | 0.000 | 1.000 |
| Mdor1817 | male | 117 | - | 288 | - | 083 | - | 150 | - | 186 | - | 175 | - | 244 | - | 146 | - | 1.000 | 0.000 |
| Mdor1821 | female | 115 | 127 | 294 | 294 | 097 | 099 | 152 | 152 | 186 | 186 | 177 | 189 | 228 | 244 | 146 | 146 | 1.000 | 0.000 |
| Mdor1823 | female | 115 | 123 | 288 | 288 | 085 | 097 | 150 | 150 | 186 | 186 | 161 | 163 | 228 | 228 | 137 | 146 | 0.000 | 1.000 |
| Mdor1824 | male | 123 | - | 288 | - | 097 | - | 150 | - | 186 | - | 161 | - | 236 | - | 146 | - | 0.001 | 0.999 |
| Mdor1826 | female | 109 | 121 | 294 | 294 | 083 | 099 | 150 | 150 | 186 | 186 | 175 | 183 | 226 | 228 | 143 | 146 | 1.000 | 0.000 |
| Mdor1832 | male | 119 | - | 288 | - | 083 | - | 152 | - | 186 | - | 175 | - | 236 | - | 146 | - | 0.994 | 0.006 |
| Mdor1839 | female | 117 | 119 | 288 | 294 | 083 | 085 | 150 | 150 | 186 | 186 | 173 | 181 | 228 | 244 | 146 | 146 | 1.000 | 0.000 |
| Mdor1867 | female | 109 | 109 | 288 | 294 | 099 | 101 | 150 | 152 | 186 | 186 | 175 | 175 | 244 | 244 | 143 | 146 | 1.000 | 0.000 |
| Mdor1870 | male | 121 | - | 294 | - | 083 | - | 150 | - | 186 | - | 175 | - | 226 | - | 146 | - | 1.000 | 0.000 |
| Mdor1873 | male | 115 | - | 288 | - | 099 | - | 150 | - | 186 | - | 163 | - | 230 | - | 146 | - | 0.010 | 0.990 |
| Mdor1874 | male | 115 | - | 288 | - | 083 | - | 150 | - | 186 | - | 159 | - | 224 | - | 146 | - | 0.066 | 0.934 |
| Mdor1875 | male | 124 | - | 288 | - | 083 | - | 152 | - | 186 | - | 159 | - | 226 | - | 153 | - | 0.054 | 0.946 |
| Mdor1876 | female | 123 | 123 | 288 | 288 | 083 | 083 | 150 | 150 | 186 | 186 | 159 | 159 | 230 | 230 | 143 | 146 | 0.000 | 1.000 |
| Mdor1878 | female | 115 | 117 | 288 | 294 | 083 | 083 | 150 | 150 | 186 | 186 | 187 | 187 | 228 | 230 | 146 | 149 | 1.000 | 0.000 |
| Mdor2005 | male | 117 | - | 294 | - | 099 | - | 150 | - | 186 | - | 175 | - | 244 | - | 146 | - | 1.000 | 0.000 |
| Mdor2010 | female | 109 | 121 | 288 | 288 | 083 | 097 | 150 | 150 | - | - | 157 | 179 | 228 | 244 | 146 | 146 | 1.000 | 0.000 |
| Mdor2187 | female | 115 | 123 | 288 | 288 | 083 | 085 | 150 | 150 | 186 | 186 | 161 | 163 | 228 | 236 | 137 | 146 | 0.000 | 1.000 |
| Mdor2188 | female | 115 | 123 | 288 | 288 | 083 | 085 | 150 | 150 | 186 | 186 | 161 | 163 | 228 | 228 | 137 | 146 | 0.000 | 1.000 |
| Mdor2189 | female | 115 | 123 | 288 | 288 | 085 | 097 | 150 | 150 | 186 | 186 | 161 | 163 | 228 | 236 | 137 | 146 | 0.000 | 1.000 |
| Mdor2190 | female | 115 | 123 | 288 | 288 | 085 | 097 | 150 | 150 | 186 | 186 | 161 | 163 | 228 | 236 | 137 | 146 | 0.000 | 1.000 |
| Mdor2192 | female | 115 | 123 | 288 | 288 | 085 | 097 | 150 | 150 | 186 | 186 | 161 | 163 | 230 | 246 | 143 | 146 | 0.000 | 1.000 |
| Mdor2193 | male | 123 | - | 288 | - | 083 | - | 150 | - | 186 | - | 161 | - | 228 | - | 146 | - | 0.001 | 0.999 |
| Mdor2196 | male | 123 | - | 288 | - | 083 | - | 150 | - | 186 | - | 161 | - | 228 | - | 146 | - | 0.001 | 0.999 |
| Mdor2198 | female | 121 | 124 | 288 | 288 | 083 | 101 | 150 | 150 | 186 | 186 | 163 | 163 | 228 | 234 | 141 | 143 | 0.000 | 1.000 |
| Mdor2201 | female | 109 | 115 | 294 | 294 | 085 | 103 | 150 | 150 | 186 | 186 | 175 | 187 | 228 | 246 | 139 | 146 | 1.000 | 0.000 |
| Mdor2202 | male | 117 | - | 294 | - | 083 | - | 152 | - | 186 | - | 175 | - | 244 | - | 146 | - | 1.000 | 0.000 |
| Mdor2203 | female | 115 | 117 | 288 | 288 | 083 | 087 | 150 | 150 | 186 | 186 | 159 | 175 | 228 | 244 | 146 | 146 | 1.000 | 0.000 |
| Mdor2204 | male | 127 | - | 290 | - | 083 | - | 152 | - | 186 | - | 175 | - | 244 | - | 146 | - | 0.999 | 0.001 |
| Mdor2307 | female | 123 | 123 | 290 | 290 | 099 | 101 | 150 | 150 | 186 | 186 | 159 | 159 | 224 | 234 | 146 | 146 | 0.000 | 1.000 |
| Mdor2313 | female | 115 | 123 | 288 | 288 | 083 | 085 | 152 | 152 | 186 | 186 | 159 | 163 | 228 | 228 | 146 | 156 | 0.000 | 1.000 |
| Mdor2317 | female | 127 | 137 | 288 | 288 | 090 | 099 | 150 | 150 | 186 | 186 | 165 | 165 | 224 | 228 | 137 | 137 | 0.000 | 1.000 |
| Mdor2337 | male | 118 | - | 294 | - | 099 | - | 150 | - | 186 | - | 175 | - | 244 | - | 146 | - | 1.000 | 0.000 |
| Mdor2338 | male | 127 | - | 294 | - | 083 | - | 150 | - | 186 | - | 159 | - | 228 | - | 143 | - | 0.945 | 0.055 |
| Mdor2346 | male | 127 | - | 288 | - | 097 | - | 150 | - | 186 | - | 173 | - | 244 | - | 146 | - | 0.999 | 0.001 |
| Mdor2353 | male | 123 | - | 288 | - | 085 | - | 150 | - | 186 | - | 159 | - | 230 | - | 137 | - | 0.000 | 1.000 |
| Mdor2356 | female | 109 | 115 | 288 | 294 | 085 | 099 | 150 | 152 | 186 | 186 | 159 | 159 | 226 | 244 | 146 | 146 | 1.000 | 0.000 |
| Mdor2368 | male | 125 | - | 288 | - | 083 | - | 152 | - | 186 | - | 161 | - | 230 | - | 146 | - | 0.000 | 1.000 |
| Mdor2388 | female | 123 | 123 | 288 | 288 | 083 | 097 | 150 | 150 | 186 | 186 | 163 | 167 | 226 | 230 | 141 | 143 | 0.000 | 1.000 |
| Mdor2390 | female | 123 | 133 | 288 | 288 | 083 | 088 | 150 | 152 | 186 | 186 | 161 | 161 | 230 | 236 | 137 | 143 | 0.000 | 1.000 |
| Mdor2391 | female | 123 | 123 | 288 | 288 | 083 | 099 | 150 | 150 | 186 | 186 | 159 | 159 | 232 | 236 | 137 | 146 | 0.000 | 1.000 |
| Mdor2392 | female | 123 | 123 | 288 | 288 | 083 | 103 | 150 | 152 | 186 | 186 | 159 | 165 | 226 | 230 | 143 | 149 | 0.000 | 1.000 |
| Mdor2393 | female | 115 | 117 | 288 | 294 | 083 | 083 | 150 | 150 | 186 | 186 | 159 | 187 | 232 | 246 | 146 | 146 | 1.000 | 0.000 |
| Mdor2394 | female | 109 | 115 | 288 | 294 | 083 | 083 | 150 | 150 | 186 | 186 | 175 | 183 | 228 | 246 | 143 | 146 | 1.000 | 0.000 |
| Mdor2395 | female | 115 | 131 | 288 | 294 | 083 | 083 | 150 | 150 | 186 | 186 | 177 | 185 | 230 | 248 | 146 | 149 | 0.999 | 0.001 |
| Mdor2396 | female | 115 | 117 | 294 | 294 | 083 | 099 | 150 | 150 | 186 | 186 | 175 | 183 | 228 | 246 | 143 | 146 | 1.000 | 0.000 |
| Mdor2529 | female | 115 | 123 | 288 | 294 | 083 | 088 | 152 | 152 | 186 | 186 | 175 | 179 | 246 | 248 | 146 | 146 | 1.000 | 0.000 |
| Mdor2530 | male | 121 | - | 294 | - | 083 | - | 150 | - | 186 | - | 187 | - | 246 | - | 146 | - | 1.000 | 0.000 |
| Mdor2531 | male | 127 | - | 288 | - | 083 | - | 152 | - | 186 | - | 159 | - | 228 | - | 143 | - | 0.246 | 0.754 |
| Mdor2532 | female | 125 | 125 | 288 | 288 | 083 | 088 | 150 | 152 | 186 | 186 | 159 | 171 | 230 | 230 | 137 | 146 | 0.000 | 1.000 |
| Mdor2533 | female | 121 | 123 | 288 | 288 | 083 | 101 | 150 | 150 | 186 | 186 | 159 | 161 | 230 | 232 | 139 | 139 | 0.000 | 1.000 |
| Mdor2604 | female | 121 | 125 | 288 | 288 | 088 | 099 | 150 | 150 | 186 | 186 | 161 | 163 | 230 | 234 | 137 | 147 | 0.000 | 1.000 |
| Mdor2605 | female | 109 | 127 | 288 | 294 | 083 | 099 | 150 | 152 | 186 | 186 | 159 | 179 | 232 | 248 | 146 | 146 | 1.000 | 0.000 |
| Number of alleles | | 17 | | 3 | | 10 | | 3 | | 7 | | 17 | | 10 | | 14 | |  |  |
| H_e_ | | 0.84 | | 0.44 | | 0.74 | | 0.39 | | 0.11 | | 0.88 | | 0.85 | | 0.60 | |  |  |
